# Identification of an essential regulator controlling the production of raw-starch-digesting glucoamylase in *Penicillium oxalicum*

**DOI:** 10.1186/s13068-018-1345-z

**Published:** 2019-01-04

**Authors:** Mei-Yuan Zhang, Shuai Zhao, Yuan-Ni Ning, Li-Hao Fu, Cheng-Xi Li, Qi Wang, Ran You, Chen-Ying Wang, Han-Nan Xu, Xue-Mei Luo, Jia-Xun Feng

**Affiliations:** State Key Laboratory for Conservation and Utilization of Subtropical Agro-bioresources, Guangxi Research Center for Microbial and Enzyme Engineering Technology, College of Life Science and Technology, Guangxi University, 100 Daxue Road, Nanning, 530004 Guangxi People’s Republic of China

**Keywords:** Transcription regulation, *Penicillium oxalicum*, Raw-starch-digesting glucoamylase, Amylase

## Abstract

**Background:**

Raw-starch-digesting glucoamylases (RSDGs) from filamentous fungi have great commercial values in starch processing; however, the regulatory mechanisms associated with their production in filamentous fungi remain unknown. *Penicillium oxalicum* HP7-1 isolated by our laboratory secretes RSDG with suitable properties but at low production levels. Here, we screened and identified novel regulators of RSDG gene expression in *P*. *oxalicum* through transcriptional profiling and genetic analyses.

**Results:**

*Penicillium oxalicum* HP7-1 transcriptomes in the presence of glucose and starch, respectively, used as the sole carbon source were comparatively analyzed, resulting in screening of 23 candidate genes regulating the expression of RSDG genes. Following deletion of 15 of the candidate genes in the parental *P*. *oxalicum* strain ∆*PoxKu70*, enzymatic assays revealed five mutants exhibiting significant reduction in the production of raw-starch-digesting enzymes (RSDEs). The deleted genes (*POX01907*, *POX03446*, *POX06509*, *POX07078*, and *POX09752*), were the first report to regulate RSDE production of *P*. *oxalicum*. Further analysis revealed that ∆*POX01907* lost the most RSDE production (83.4%), and that *POX01907* regulated the expression of major amylase genes, including the RSDG gene *POX01356/PoxGA15A*, a glucoamylase gene *POX02412*, and the α-amylase gene *POX09352/Amy13A*, during the late-stage growth of *P*. *oxalicum*.

**Conclusion:**

Our results revealed a novel essential regulatory gene *POX01907* encoding a transcription factor in controlling the production of RSDE, regulating the expression of an important RSDG gene *POX01356*/*PoxGA15A*, in *P*. *oxalicum*. These results provide insight into the regulatory mechanism of fungal amylolytic enzyme production.

**Electronic supplementary material:**

The online version of this article (10.1186/s13068-018-1345-z) contains supplementary material, which is available to authorized users.

## Background

Starch is an important renewable carbohydrate biosynthesized in large quantities through plant photosynthesis. Starch biorefinery can provide a variety of useful chemicals, including biofuels. In traditional starch biorefinery, amylases, including α-amylase (EC 3.2.1.1) and glucoamylase (EC 3.2.1.3), are used to hydrolyze starch to glucose [1]. To reduce the energy costs associated with traditional starch biorefinery, raw-starch-digesting enzymes (RSDEs), specifically raw-starch-digesting glucoamylases (RSDGs), represent promising alternatives capable of directly degrading raw starch granules into oligosaccharides or glucose below the gelatinization temperature of starch [2].

Native RSDEs are primarily produced by filamentous fungi, such as *Aspergillus* sp. [3, 4], *Penicillium* sp. [5], and *Aureobasidium pullulans* [6]. Recently, a novel RSDG (PoxGA15A) exhibiting suitable properties was identified in *Penicillium oxalicum* and showed remarkably broad pH stability and substrate specificity. Simultaneous saccharification and fermentation of either raw cassava or corn flour using the recombinant protein rPoxGA15A from *Pichia pastoris* combined with the presence of commercial α-amylase resulted in high fermentation efficiency (> 90%) [5]. However, both native PoxGA15A and rPoxGA15A production, as well as that of other RSDGs from fungi, such as *Rhizopus* sp. A-11 [7], *Aspergillus fumigatus* CFU-01 [8] and *Laceyella sacchari* LP175 [9], are too low, which limit their industrial application. Notably, the expression of fungal RSDG genes is strictly controlled by transcription factors (TFs) at the transcriptional level. Genetic engineering of fungal strains based on constructed TF-specific regulatory networks and targets represents an efficient method to improve RSDG production.

Few studies associated with the regulation of RSDG gene expression in filamentous fungi, including *Aspergillus* sp., *Talaromyces pinophilus*, *P. oxalicum*, and *Neurospora crassa* have been undertaken. Previous studies described two Zn(II)2Cys6 zinc finger proteins (AmyR [10] and COL-26 [11]), a heterotrimeric G protein subunit (PGA3 [12]), and casein kinase CK2 proteins (CK2B1 and CK2B2) as activators of *Amy15A* (a *PoxGA15A* homolog) gene expression [13]. In addition to these activators, an extracellular protease activator, PrtT [14], and an HMG-box protein, PoxHmbB [15], were identified as *Amy15A* repressors in *P. oxalicum*. However, these findings are insufficient to elucidate the regulatory mechanism associated with RSDG gene expression for improving RSDG production.

In this study, we employed RNA-seq and molecular genetic technologies to screen and identify novel regulators of RSDE production and RSDG gene expression in *P*. *oxalicum*. Transcriptomes from *P*. *oxalicum* grown in the presence of glucose or soluble corn starch (SCS) were profiled to identify candidate regulators of RSDE production. Subsequent knockout of candidate genes, measurement of enzyme activity in the resulting mutants, and expression analysis of amylase genes, including the RSDG gene *PoxGA15A*, were performed to identify novel regulators of RSDG gene expression in *P*. *oxalicum*.

## Results

### Transcriptome profiling and screening of candidate regulators of RSDE production in *P*. *oxalicum*

Genome-wide screening of candidate regulators of RSDE production was undertaken through RNA-seq analysis of the transcriptome profiles of *P*. *oxalicum* grown on media containing glucose or SCS as the sole carbon source following a transfer from glucose. In the presence of glucose, carbon catabolite repression is activated and inhibits RSDE production in *P*. *oxalicum*, whereas SCS stimulates the secretion of RSDEs. Total RNAs were extracted from the mycelia of *P*. *oxalicum* grown on glucose or SCS for 4 h and then sequenced. Approximately 24 million clean reads at 100 bp in length (Accession Number SRP116594 in Sequence Read Archive [SRA] database) were generated from each sample, with > 90% mapped into the genome of *P*. *oxalicum* wild-type strain HP7-1 [16] (Additional file 1: Table S1). High Pearson’s correlation coefficients among three biological replicates of *P*. *oxalicum* under each culture condition (*r* ≥ 0.96) (Additional file 2: Figure S1) indicated the reliability of the transcriptome data. Gene-expression levels were quantitatively analyzed according to fragments per kilobase of exon per million mapped reads (FPKM), and the differences were evaluated using NOISeq [17].

**Fig. 1:**
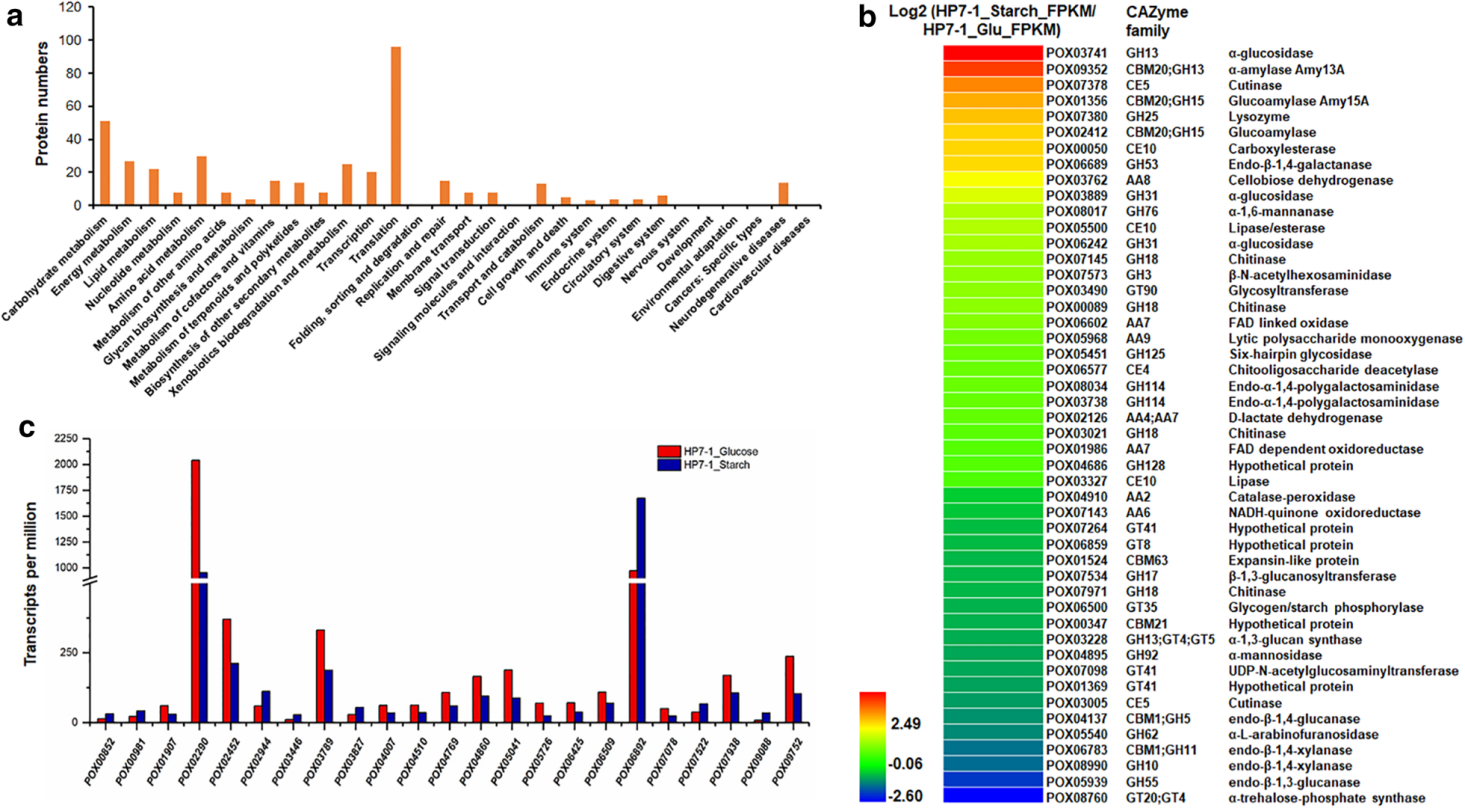
Comparative analysis of transcriptomes from *Penicillium oxalicum* HP7-1 grown in the presence of glucose and soluble corn starch. **a** Kyoto encyclopedia of genes and genomes annotation of proteins encoded by differentially expressed genes (DEGs) between HP7-1 grown in soluble corn starch medium relative to that grown in glucose medium. DEGs were screened using a probability threshold of ≥ 0.6 and a |log_2_ fold change| of > 0.5. **b** Heatmap showing the transcription levels of DEGs encoding carbohydrate-active enzymes (CAZymes). **c** The transcription levels of DEGs encoding putative transcription factors

Comparative transcriptome analyses identified 961 differentially expressed genes (DEGs), with 681 upregulated (0.52 < log_2_ fold change < 10.18) and 280 downregulated (− 10.26 < log_2_ fold change < − 0.54) in *P*. *oxalicum* HP7-1 on SCS as compared with that in HP7-1 on glucose (Additional file 3: Table S2). Kyoto Encyclopedia of Genes and Genomes (KEGG) annotation revealed that these DEGs were mainly involved in metabolic pathways, including carbohydrate, amino acid, and energy metabolism, and translation (Fig. 1a).

Of the DEGs, 48 were annotated to encode carbohydrate-active enzymes (CAZymes), including six auxiliary activity families, 18 glycoside hydrolase families, seven glycosyl transferase families, three carbohydrate esterase families, and four carbohydrate-binding-module families. Among these 48 DEGs, 28 were upregulated, with a log_2_ fold change from 0.72 to 5.03 in HP7-1 on SCS relative to that in HP7-1 on glucose. As expected, these included 60% of the amylase genes in the whole genome of *P*. *oxalicum* HP7-1, including a key RSDG gene (*POX01356*/*PoxGA15A*) and a glucoamylase gene (*POX02412*), a key α-amylase gene (*POX09352*/*Amy13A*), and three α-glucosidase genes (*POX03741*, *POX03889*, and *POX06242*). Surprisingly, the important genes involved in the degradation of chitin and plant-cell walls were also found in the upregulated gene set, including three chitinase genes (*POX00089*/*ChiB1*, *POX03021*, and *POX07145*), a chitooligosaccharide deacetylase gene (*POX06577*), a lytic polysaccharide monooxygenase gene (*POX05968*), and an endo-β-1,4-galactanase gene (*POX06689*). In contrast, among the 20 downregulated DEGs, five encoded plant-cell-wall-degrading enzymes (CWDEs), including an expansin-like protein-encoding gene *POX01524*, an endo-β-1,4-glucanase gene *POX04137*, two endo-β-1,4-xylanase genes (*POX06783/Xyn11A* and *POX08990*), and a xylosidase gene (*POX05540*), and a chitinase gene (*POX07971*), with all exhibiting log_2_ fold changes from − 1.52 to − 0.77. However, no amylase genes were included (Fig. 1b).

Additionally, comparative analyses also revealed 23 DEGs encoding putative TFs as candidate regulators (Additional file 4: Table S3), most of which contained at least one zinc finger domain (C2H2, GATA, Zn2Cys6, or DHHC). Eight candidate DEGs increased target transcript level by 72.2%–316.7% in HP7-1 on SCS relative to that in HP7-1 on glucose, whereas 15 genes showed lower transcript levels (by 36.5–64.7%) (Fig. 1c). POX04510, a protein homolog of AreA in *Aspergillus nidulans*, negatively controls cellulase production [18]. Moreover, *POX02944* and *POX03789* encode protein homologs of OefC and StuA, respectively, that regulate sporulation in filamentous fungi, including *A. nidulans* [19, 20]. Recently, *POX04860* and *POX05726* were found to regulate cellulase production in *P*. *oxalicum* [21].

### Five novel regulators are required for RSDE production in *P*. *oxalicum*

**Fig. 2:**
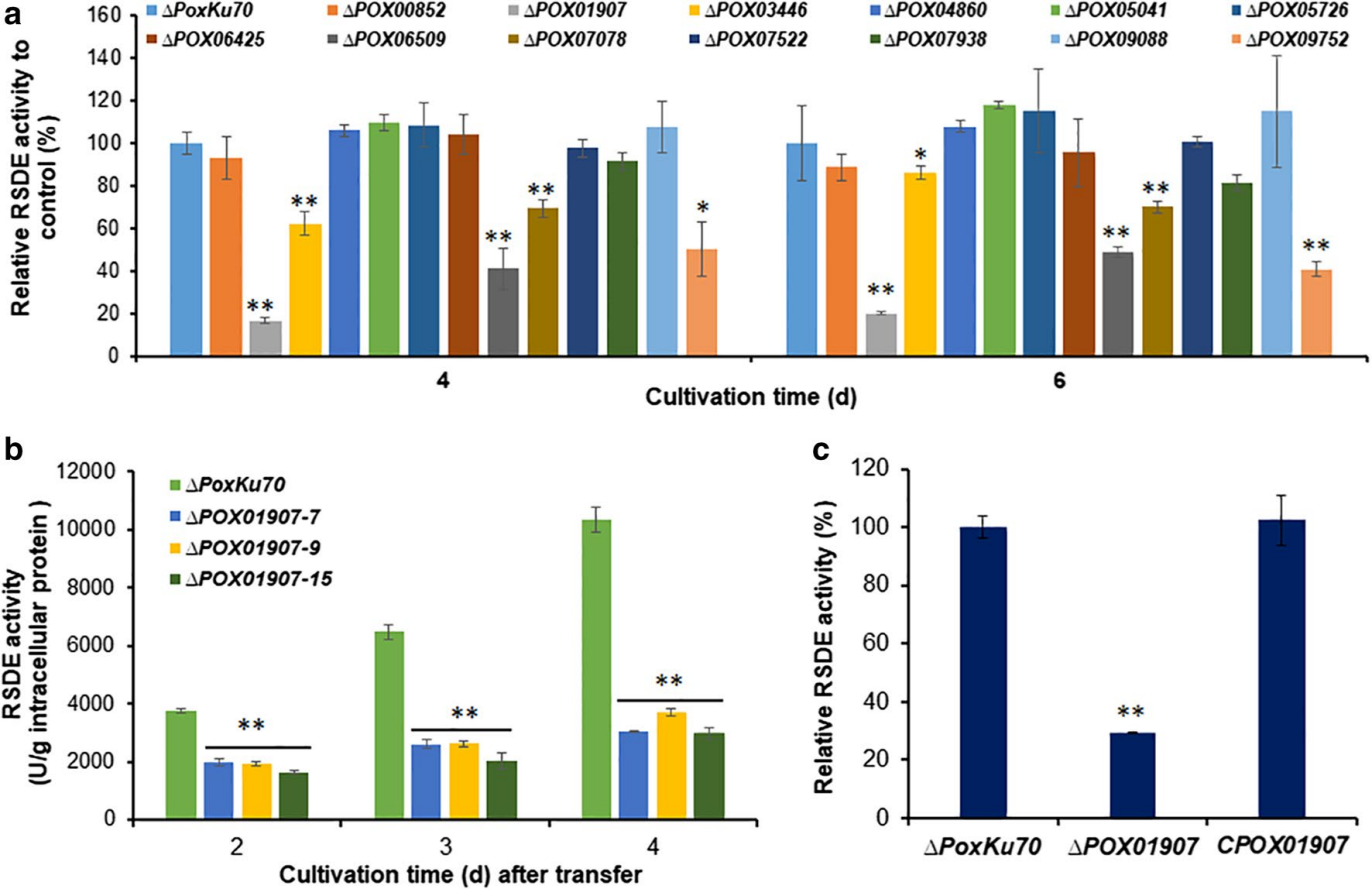
Screening and identification of novel regulatory genes required for the production of raw starch-degrading enzymes (RSDEs) in *Penicillium oxalicum*. **a** RSDE activities in strains with deletion of candidate regulatory genes and grown in medium containing soluble corn starch as the sole carbon source for 4–6 days after direct inoculation. The parental strain ∆*PoxKu70* was used a control. Asterisks indicate significant differences (**P < 0.01; *P < 0.05) between the deletion mutants and the parental strain ∆*PoxKu70* according to Student’s *t* test. **b**, **c** RSDE activities in the deletion mutants ∆*POX01907* and ∆*PoxKu70* and the complementary strain C*POX01907*. *P*. *oxalicum* strains were cultivated in medium containing soluble corn starch for 2–4 days after transfer from glucose. Asterisks indicate significant differences (**P < 0.01) between ∆*POX01907* and ∆*PoxKu70* or C*POX01907*, as assessed by Student’s *t* test

To investigate the regulatory roles of these 23 candidate TF-encoding DEGs in RSDE production in *P*. *oxalicum*, homologous recombinant technology was employed for their deletion from the parental strain ∆*PoxKu70* [16]. Eleven deletion mutants (∆*POX00852*, ∆*POX01907*, ∆*POX03446*, ∆*POX03789*, ∆*POX05041*, ∆*POX06509*, ∆*POX07078*, ∆*POX07522*, ∆*POX07938*, ∆*POX09088*, and ∆*POX09752*) were successfully constructed in this present study, and four deletion mutants (∆*POX02944*, ∆*POX04860*, ∆*POX05726,* and ∆*POX06425*) had been constructed in the previously published work [21]. These 11 newly constructed deletion mutants in this study were verified by polymerase chain reaction (PCR) using specific primers (Additional file 5: Figure S2a and Additional file 6: Table S3). Both ∆*POX03789* and ∆*POX02944* were unable to produce spores (data not shown), which was consistent with previously described results [19, 20]. Assays of RSDE activity undertaken on all of the deletion mutants, except for *∆POX03789* and *∆POX02944,* grown on medium containing SCS as the sole carbon source for 4–6 days after direct inoculation revealed that five deletion mutants (∆*POX01907*, ∆*POX03446*, ∆*POX06509*, ∆*POX07078*, and ∆*POX09752*) showed significant reduction in RSDE production relative to the parental strain *∆PoxKu70*; ranking from 30 to 83.4% (*P* ≤ 0.05, Student’s *t* test) (Fig. 2a). This represents the first report showing that these five DEGs were involved in RSDE production in *P*. *oxalicum* (Table 1). Strikingly, the mutant *∆POX01907* showed higher losses of RSDE activity (83.4% at day 4 and 80.0% at day 6) relative to the other four mutants and was subsequently selected for further study. To exclude the possibility that multiple copies of *POX01907*-deletion cassette were integrated into the *∆PoxKu70* genome, the mutant *∆POX01907* was further confirmed by Southern hybridization analysis (Additional file 5: Figure S2b) using specific probes (Additional file 6: Table S3).

**Table 1:** Novel genes encoding TFs involved in regulating the production of raw-starch-degrading enzymes in *P. oxalicum*

Gene ID	GenBank accession number	InterPro annotation	Conversed domain	Known homologous TFs	Identity (%)	RSDE activity of mutant relative to the parental strain (%)^a^
*POX01907*	MH742968	IPR001005	SANT/Myb DNA binding domain	NA	NA	16.6 ± 1.2
*POX03446*	MH742969	IPR001138 IPR007219	Zinc finger, Zn2Cys6 type Fungal_Trans	*Aspergillus niger* CBS 513.88 AraR (A2QJX5.1)	71%	62.2 ± 5.4
*POX06509*	MH742972	IPR004827	Basic-leucine zipper (bZIP)	NA	NA	40.9 ± 9.5
*POX07078*	MH742970	IPR009057 IPR001025 IPR001965	Homeodomain-like Bromo adjacent homology (BAH) domain Zinc finger, PHD-type	NA	NA	69.4 ± 4.1
*POX09752*	MH742971	IPR001138 IPR001451 IPR024688	Zn2Cys6 fungal-type DNA-binding domain Hexapeptide repeat Maltose/galactoside acetyltransferase	NA	NA	50.1 ± 12.5

RSDE: raw starch-digesting enzyme; SCS: soluble corn starch; TF: transcription factor

^a^RSDE activity was measured from the crude extract of *P*. *oxalicum* grown in medium containing SCS for 4 days after direct inoculation

### *POX01907* regulates RSDE production in *P*. *oxalicum* following SCS induction

To elucidate *POX01907*-specific regulatory roles in RSDE production, *P*. *oxalicum* strains ∆*POX01907* and ∆*PoxKu70* were grown on medium containing SCS as the sole carbon source for 2–4 days after a transfer from glucose, followed by real-time investigation. The results revealed 43.5%–71.3% reduction in RSDE production by the mutant ∆*POX01907* relative to that observed in the parental strain ∆*PoxKu70* (Fig. 2b), which was consistent with our previous analyses.

To confirm the reduction in RSDE production in ∆*POX01907* as being a result of *POX01907* deletion, a complementary strain (C*POX01907*) was constructed and confirmed by PCR (Additional file 5: Figure S2c) using specific primer pairs (Additional file 6: Table S3). Enzyme assays indicated no significant difference in RSDE production between C*POX01907* and ∆*PoxKu70* (Fig. 2c).

### Deletion of *POX01907* promotes *P*. *oxalicum* mycelial growth during the late stage of SCS induction

**Fig. 3:**
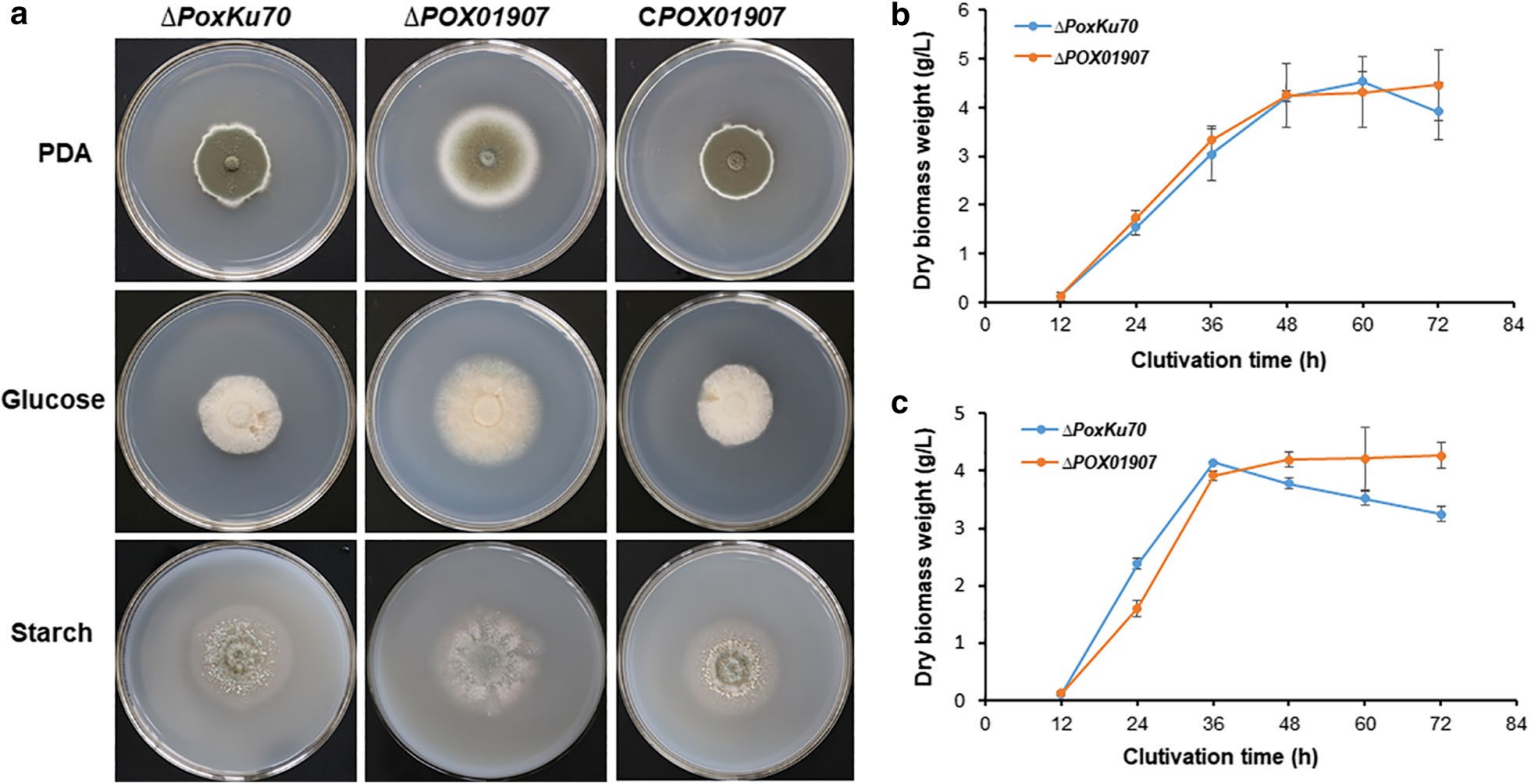
Phenotype and growth comparison of ∆*POX01907*, the complementary strain C*POX01907*, and the parental strain ∆*PoxKu70* on different media. **a** Colonies on solid plates containing PDA, starch, and glucose, respectively, after incubation at 28 °C for 5 days. **b**, **c** Growth profiles of ∆*POX01907* and ∆*PoxKu70* in the presence of glucose and soluble corn starch, respectively. Data represent the means of three biological replicates

Equal amounts of fresh spores collected from *P. oxalicum ∆PoxKu70*, the deletion mutant *∆POX01907*, and the complementary strain C*POX01907* were inoculated on solid-medium plates in the presence of glucose, soluble corn starch as the sole carbon source, and potato dextrose agar (PDA), respectively, and cultured at 28 °C for 5 days. The results indicated that colony diameter of *∆POX01907* on all tested plates was significantly larger than that of *∆PoxKu70* and C*POX01907*, which showed similar colony diameter (Fig. 3a). Moreover, the colony color of *∆POX01907* was lighter than that of *∆PoxKu70* and C*POX01907*, which shared similar colony color (Fig. 3a).

Additionally, we measured mycelial biomass in the three *P*. *oxalicum* strains grown in liquid media containing glucose or SCS. The mycelial weight of *∆POX01907* grown in glucose medium was similar to that of *∆PoxKu70* at 60-h post-inoculation and increased slightly after 60 h (Fig. 3b). The mycelial biomass of *∆POX01907* in SCS medium was slightly lower relative to that of *∆PoxKu70* at 36-h post-inoculation but increased significantly after 36 h (Fig. 3c).

### *POX01907* dynamically regulates the expression of major amylase genes in *P. oxalicum*

**Fig. 4:**
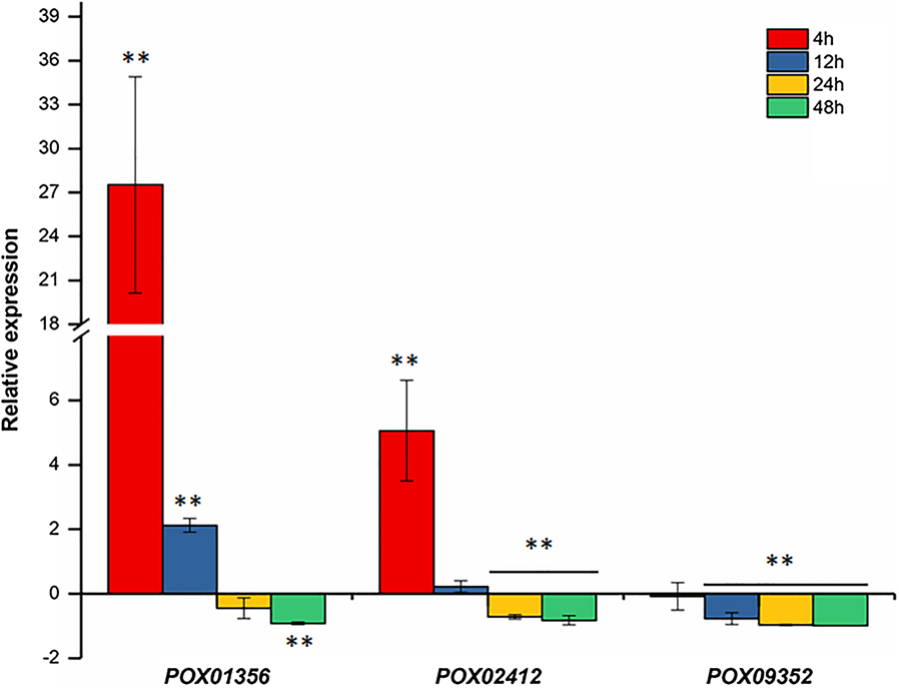
Regulated expression of the major amylase genes by POX01907 in *Penicillium oxalicum* under the induction of soluble corn starch according to real-time quantitative reverse transcription polymerase chain reaction analysis. Transcript levels of amylase genes in the ∆*POX01907* and ∆*PoxKu70* investigated at 4, 12, 24 and 48 h after a shift from glucose to soluble corn starch. Expression levels were normalized against that in the parental strain ∆*PoxKu70*. Asterisks indicate significant differences (**P < 0.01) between ∆*POX01907* and ∆*PoxKu70*, as assessed by Student’s *t* test

To investigate the regulatory roles of *POX01907* in the expression of amylase genes in *P*. *oxalicum*, real-time quantitative reverse transcription PCR (RT-qPCR) was performed using total RNA from ∆*PoxKu70* and ∆*POX01907* grown on SCS medium for 4 h–48 h after a shift from glucose. Three major amylase genes were selected for evaluation, including an α-amylase gene *POX09352/Amy13A*, the RSDG gene *POX01356/PoxGA15A*, and a glucoamylase gene *POX02412*. The results revealed that transcript levels of *POX01356/PoxGA15A* and *POX02412* in ∆*POX01907* increased by 2752.1% and 506.0%, respectively, relative to those in the parental strain *∆PoxKu70* at 4 h post-SCS induction, whereas we did not observe changes in *POX09352/Amy13A* transcript levels. At 12 h, only *POX01356*/*PoxGA15A* continued to show elevation in transcript level in *∆POX01907* (by 212.3%), whereas *POX09352/Amy13A* transcript levels began to decrease (by 77.3%). The expression of all three genes was reduced by 71.3%–98.8% in *∆POX01907* after 12 h of SCS induction, although *POX01356/PoxGA15A* transcript level showed no significant difference from that in the ∆*PoxKu70* at 24 h (Fig. 4).

### RNA-seq analyses reveal broad regulation of *POX01907* in *P. oxalicum*

To analyze genome-wide regulation of *POX01907* under SCS induction in *P*. *oxalicum*, RNA-seq was employed using total RNA collected upon a transfer of the deletion mutants ∆*POX01907* and ∆*PoxKu70* from glucose to SCS medium, followed by a 4-h incubation. In total, approximately 22 million of clean reads (length: 100 bp) for each sample were generated (Accession Number SRP116594), with > 90% of the clean reads mapped onto the genome of *P*. *oxalicum* HP7-1 wild-type strain (Additional file 1: Table S1). To assess the reliability of the generated transcriptome data, Pearson’s correlation coefficients (*r*) were calculated among three biological replicates for each sample, with results indicating high correlation (*r* > 0.95) (Additional file 7: Figure S3) and confirming their accuracy.

**Fig. 5:**
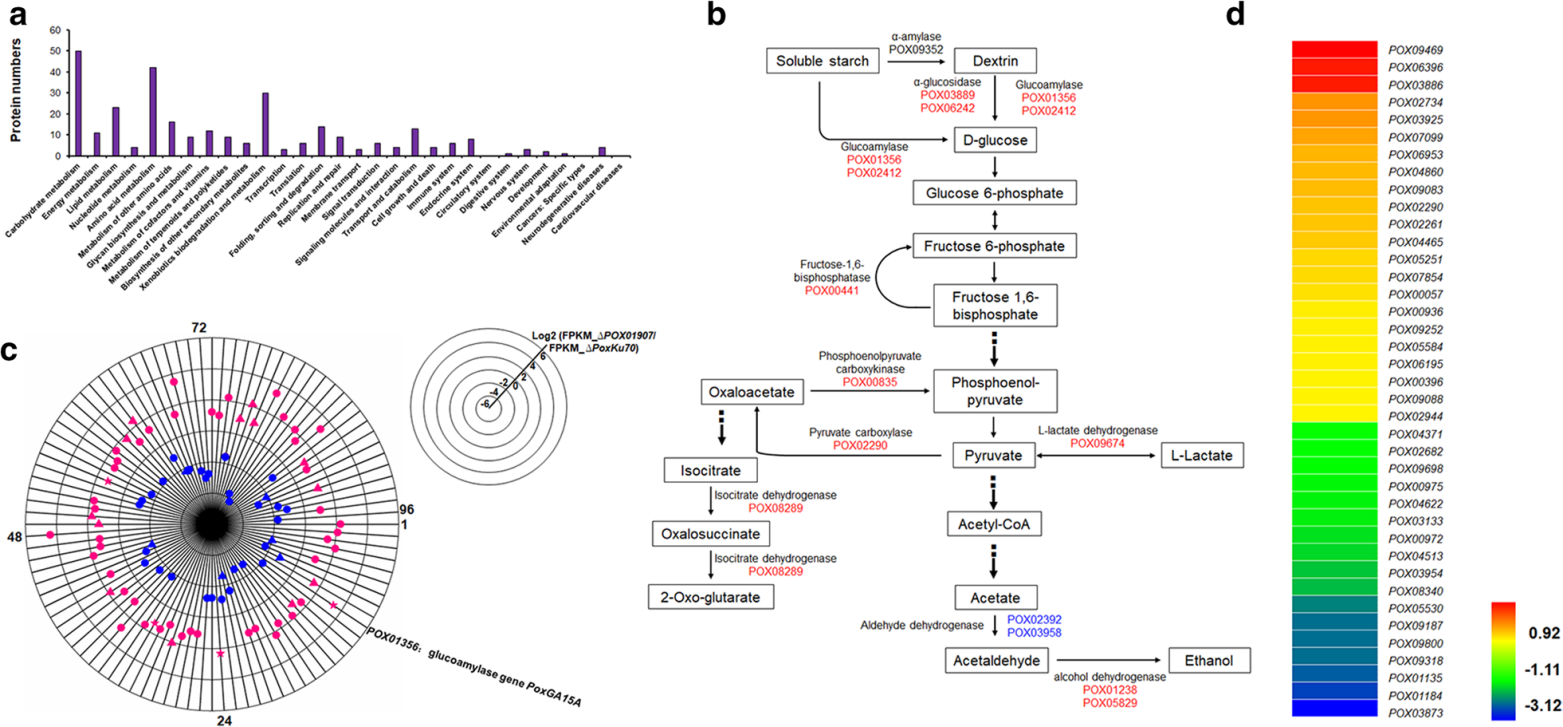
Transcriptome profiling of ∆*POX01907* and the parental strain ∆*PoxKu70* during growth in the presence of soluble corn starch as the carbon source. **a** Kyoto encyclopedia of genes and genomes annotation of proteins encoded by differentially expressed genes (DEGs) in ∆*POX01907* relative to ∆*PoxKu70*. DEGs were identified according to a |log_2_ fold change| ≥ 1 and a *P* ≤ 0.05 as thresholds. **b** DEGs involved in glycolysis/gluconeogenesis and the TCA cycle. Upregulated DEGs are labeled in red, and downregulated DEGs are labeled in blue. **c** POX01907-specific regulation of DEGs encoding carbohydrate-active enzymes (CAZymes). Upregulated DEGs are labeled in pink, and downregulated DEGs are labeled in blue. Solid triangles represent DEGs encoding plant-cell-wall-degrading enzymes, and solid pentagrams represent DEGs encoding amylases. **d** Heatmap showing transcription levels of DEGs encoding putative transcription factors

Gene expression was evaluated according to FPKM values calculated with the software package RSEM [22], and DEGs were screened with DESeq2 [23]. Comparative analysis of transcriptomes between ∆*PoxKu70* and ∆*POX01907* revealed 1003 DEGs, including 459 downregulated (–7.3 < log_2_ fold change < − 1.0) and 544 upregulated (1.0 < log_2_ fold change < 7.2) genes within the *POX01907* regulon (Additional file 8: Table S5). KEGG annotation indicated that these DEGs were primarily involved in metabolism (72.9%), specifically carbohydrate metabolism (17.5%), amino acid metabolism (14.0%) and xenobiotic biodegradation and metabolism (10.0%) (Fig. 5a).

Nutrients and energy required by *P*. *oxalicum* are derived from the substrate SCS. Of the 10 DEGs involved in starch and sucrose metabolism, five were involved in starch degradation. Among these, two glucoamylase genes (*POX01356*/*PoxGA15A* and *POX02412*) and two α-glucosidase genes (*POX03889* and *POX06242*) showed increased expression (by 136.0–936.0%) in ∆*POX01907* relative to the parental strain ∆*PoxKu70*, whereas expression of a gene encoding a 1,4-α-glucan-branching enzyme (*POX04938*) decreased by 50.7%. Surprisingly, seven genes were involved in cellulose degradation, including a cellobiohydrolase gene (*POX05587*/*Cel7A*-*2*), four endo-β-1,4-glucanase genes (*POX05571*/*Cel7B*, *POX01206*, *POX07535*/*Cel12A*, and *POX06983*), a β-glucosidase gene (*POX07963*), and a lytic polysaccharide monooxygenase gene (*POX08897*), with all displaying log_2_ fold changes ranging from −2.1 to 2.4. Additionally, seven genes were involved in glycolysis/gluconeogenesis and included a phosphoenolpyruvate carboxykinase gene (*POX00835*), a fructose-1,6-bisphosphatase gene (*POX00441*), two alcohol dehydrogenase genes (*POX01238* and *POX05829*), two aldehyde dehydrogenase genes (*POX02392* and *POX03958*), and an l-lactate dehydrogenase gene (*POX09674*), as well as two genes [pyruvate carboxylase (*POX02290*) and isocitrate dehydrogenase (*POX08289*)] involved in the citric acid cycle (TCA cycle). The transcript levels of these DEGs, except for *POX02392* and *POX03958*, were upregulated in ∆*POX01907* by 105.6%–255.3% (Fig. 5b).

In the regulon of *POX01907*, 96 DEGs were identified as encoding CAZymes, including 35 from the glycoside hydrolase family, 11 from the glycosyl transferase family, seven from the carbohydrate esterase family, eight from families exhibiting auxiliary activity, one polysaccharide lyase, and 11 from carbohydrate-binding-module families. Among these, 35 were downregulated (− 4.2 < log_2_ fold change < − 1.0) and 61 were upregulated (1.0 < log_2_ fold change < 4.5) (Fig. 5c). The regulon mainly contained five genes encoding starch-degrading enzymes previously described in this study, 20 genes encoding CWDEs, and seven genes predicted to encode enzymes degrading chitin. Notably, ∆*POX01907* showed significant upregulation of genes encoding most of the CWDEs and amylase, and downregulation of chitin-degrading genes under SCS induction, suggesting multiple-regulation of genes involved in degrading different carbohydrates, including starch, cellulose, hemicellulose, and chitin.

Additionally, in the *POX01907* regulon, 39 genes predicted as encoding TFs were detected, including 22 upregulated with a log_2_ fold change from 1.0 to 3.0 and 17 downregulated (− 3.2 < log_2_ fold change < − 1.0) (Fig. 5d). Functional annotation indicated that > 50% these contained zinc-related structures (Zn2Cys6 and C2H2), with two regulatory genes (*POX01184* and *POX04860*/*PDE_07199*) reported to regulate cellulase gene expression in *P*. *oxalicum* [21]. However, no report was published about the regulation of these predicted TFs towards amylase genes expression.

### POX01907 contains a pair of SANT/Myb domains

**Fig. 6:**
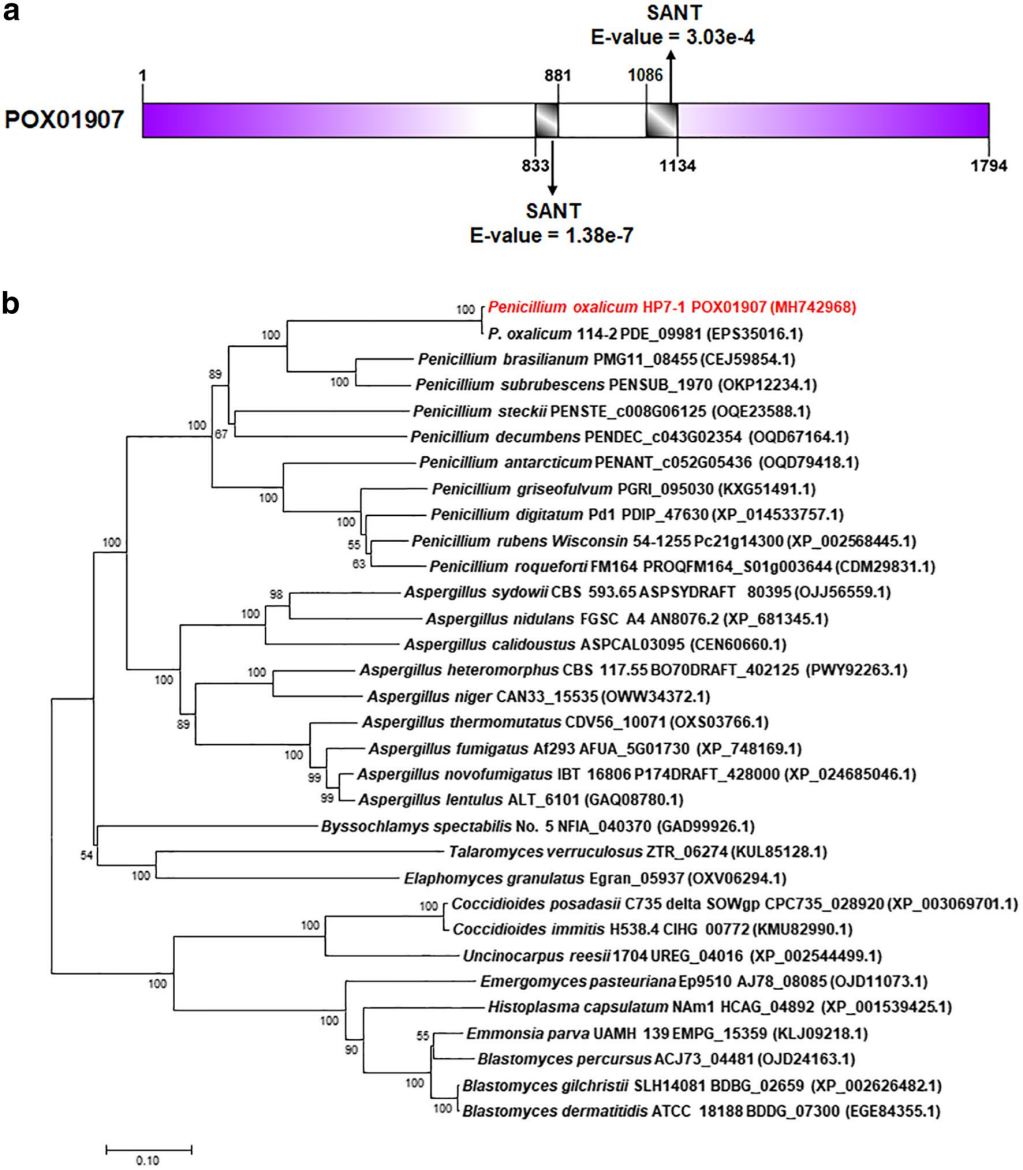
Characterization of POX01907 from *Penicillium oxalicum* HP7-1. **a** Modular architecture of POX01907. **b** Unrooted phylogenetic tree for POX01907 and its putative homologs. The phylogenetic tree was constructed using MEGA 7.0 software with the neighbor-joining method and a Poisson model. Bootstrap values shown were calculated with 1000 replicates

The POX01907 protein contains 1794 amino acids and two SANT [switching-defective protein 3 (*S*wi3), adenosine deaminase 2 (*A*da2), nuclear receptor co-repressor (*N*-CoR), and *T*FIIIB)]/Myb domains [SANT/Myb_833–881_ (IPR001005; *E*-value: 1.38e−7) and SANT/Myb_1086–1134_ (IPR001005; *E*-value 3.03e−4)] (Fig. 6a). Additionally, BlastP analysis revealed that POX01907 shares 99% and 43% of identities with PDE_09981 in *P*. *oxalicum* 114-2 and AN8076.2 in *A. nidulans* FGSC A4 (XP_681345.1), respectively. The functions of PDE_09981 in *P*. *oxalicum* 114-2 and AN8076.2 in *A. nidulans* FGSC A4 (XP_681345.1) are unknown.

A phylogenetic tree for POX01907 and its homologs was constructed, revealing specificity to filamentous fungi, especially *Penicillium* spp. and *Aspergillus* spp. (Fig. 6b).

## Discussion

In this study, we identified five novel regulatory genes (*POX01907*, *POX03446*, *POX06509*, *POX07078*, and *POX09752*) involved in mediating RSDE production in *P*. *oxalicum* through transcriptome profiling and genetic analysis. Further analyses confirmed that POX01907 regulated the expression of major amylase genes, including the RSDG gene *PoxGA15A*, as well as *P*. *oxalicum* mycelium growth in the presence of SCS. This represents the first report of POX01907 involvement in regulation of RSDG gene expression.

The SANT domain comprises an approximately 50-amino acid motif located in the subunits of many members of chromatin-remodeling complexes, such as Swi3, N-CoR, Ada2, and chromodomain-helicase-DNA binding protein 1 (Chd1), and consists of three α-helices arranged in a helix–turn–elix motif, with each α-helix containing a bulky aromatic residue and is similar to the Myb DNA-binding domain (DBD) [24]. However, the functions of SANT domains might be divergent from those of canonical Myb DBDs.

The SANT domain functions as a unique histone-interaction module that couples histone binding to enzyme catalysis and plays a central role in chromatin remodeling by regulating the activities of histone acetyltransferases and deacetylases to synergistically promote and maintain histone deacetylation [24]. SANT domains are capable of interacting with DNA (i.e., *Saccharomyces cerevisiae* Chd1 and *Arabidopsis* sp. Swc4 bind specific AT-rich DNA sequences in a non-canonical manner) [25, 26], histones [24], and other proteins (i.e., Chd1 interacts with the transcription-elongation factors Rtf1, Spt4–Spt5, and Spt6–Pob3) [27]. Additionally, the SANT protein Ada2 from *Trichoderma reesei* is required for mycelial growth, sporulation, and the expression of cellulase genes [28]. In filamentous fungi, including *P. oxalicum*, the transcription of cellulase and amylase genes is often co-regulated by several TFs, such as PoxNsdD [29], PoxAmyR [30], and PoxHmbB [15]. In the present study, comparative analysis of transcriptomes indicated that *POX01907* also regulated the transcription of several cellulase genes in *P. oxalicum* under the induction of SCS. Therefore, we speculated that POX01907 might play an essential role in the expression of amylase genes by interacting with chromatin-remodeling complexes, although this requires further confirmation.

POX01907 dynamically regulated the expression of major RSDG and α-amylase genes similar to other known TFs identified previously in *P. oxalicum* [15, 21, 29, 30] and was dependent upon the nutrient and energy needs of fungal cells. Transcriptome profiling indicated that POX01907 had minimal influence on the expression of genes involved in the glycolysis pathway. During the early period of *P*. *oxalicum* cultivation, fungal cells require trace amounts of glucose for development and growth, and POX01907 inhibited the expression of glucoamylase genes *POX01356*/*PoxGA15A* and *POX02402*, thereby avoiding carbon catabolite repression. Along with glucose consumption and cell proliferation, POX01907 initiated the transcription of genes encoding glucoamylases and α-amylases, resulting in sufficient enzyme secretion to promote the degradation of starch into glucose.

## Conclusions

Collectively, our results identified *POX01907*, a novel transcription factor gene responsible for regulating the production of RSDE through controlling the expression of the major RSDG gene *POX01356/PoxGA15A*, a glucoamylase gene (*POX02412*), and the α-amylase gene *POX09352/Amy13A*. These findings provide novel insights into the regulatory mechanism associated with fungal amylolytic enzymes production and their genes expression.

## Methods

### *P. oxalicum* strains and cultivation conditions

All *P*. *oxalicum* strains (Table 2) were cultured on PDA plates at 28 °C for 6 days to obtain fungal spores, resuspended using 0.1% Tween 80, and the concentration of fungal spores adjusted to 1.0 × 10^8^/mL. *P*. *oxalicum* strains HP7-1 and Δ*PoxKu70* were deposited in the China General Microbiological Culture Collection (Beijing, China) with Accession Numbers 10781 and 3.15650, respectively.

**Table 2:** *Penicillium oxalicum* strains used in this work

Strains	Genotypes^a^	References
HP7-1	Wild-type	[16]
∆*PoxKu70*	*PoxKu70*^−^; *Hph*^R+^	[16]
∆*POX00852*	*PoxKu70*^−^; *Hph*^R+^; *POX00852*^−^; *G418*^R+^	This study
∆*POX01907*	*PoxKu70*^−^; *Hph*^R+^; *POX01907*^−^; *G418*^R+^	This study
∆*POX02944*	*PoxKu70*^−^; *Hph*^R+^; *POX02944*^−^; *G418*^R+^	[21]
∆*POX03446*	*PoxKu70*^−^; *Hph*^R+^; *POX03446*^−^; *G418*^R+^	This study
∆*POX03789*	*PoxKu70*^−^; *Hph*^R+^; *POX03789*^−^; *G418*^R+^	This study
∆*POX04860*	*PoxKu70*^−^; *Hph*^R+^; *POX04860*^−^; *G418*^R+^	[21]
∆*POX05041*	*PoxKu70*^−^; *Hph*^R+^; *POX05041*^−^; *G418*^R+^	This study
∆*POX05726*	*PoxKu70*^−^; *Hph*^R+^; *POX05726*^−^; *G418*^R+^	[21]
∆*POX06425*	*PoxKu70*^−^; *Hph*^R+^; *POX06425*^−^; *G418*^R+^	[21]
∆*POX06509*	*PoxKu70*^−^; *Hph*^R+^; *POX06509*^−^; *G418*^R+^	This study
∆*POX07078*	*PoxKu70*^−^; *Hph*^R+^; *POX07078*^−^; *G418*^R+^	This study
∆*POX07522*	*PoxKu70*^−^; *Hph*^R+^; *POX07522*^−^; *G418*^R+^	This study
∆*POX07938*	*PoxKu70*^−^; *Hph*^R+^; *POX07938*^−^; *G418*^R+^	This study
∆*POX09088*	*PoxKu70*^−^; *Hph*^R+^; *POX09088*^−^; *G418*^R+^	This study
∆*POX09752*	*PoxKu70*^−^; *Hph*^R+^; *POX09752*^−^; *G418*^R+^	This study
C*POX01907*	*PoxKu70*^−^; *Hph*^R+^; *G418*^R+^; *Ble*^R+^	This study

*Hph*^R+^: hygromycin B resistant gene; *G418*^R+^: geneticin resistant gene; *Ble*^R+^: bleomycin resistant gene

For measurement of RSDE production, *P*. *oxalicum* strains were cultivated according to previously described methods [5], with some modifications. Under non-transferring conditions, fresh spores (1.0 × 10^8^/mL) of *P*. *oxalicum* strains were directly inoculated into minimal medium containing SCS as the sole carbon source at 28 °C for 4–6 days. Under transferring conditions, *P*. *oxalicum* spores (1.0 × 10^8^/mL) were first inoculated into minimal medium containing glucose as the carbon source for 24 h, followed by transfer of an equal amount of mycelia from each *P*. *oxalicum* strain into minimal medium containing SCS as the carbon source for incubation at 28 °C for 2–4 days. For RNA-seq and RT-qPCR analyses, *P. oxalicum* strains were cultured for 4 h–48 h according to the methods described for transferring conditions.

### Extraction of total DNA and RNA from *P*. *oxalicum*

Extraction of total DNA and RNA from *P*. *oxalicum* was performed as described previously [16]. Briefly, collected *P*. *oxalicum* mycelia were ground with liquid nitrogen, and lysate reagent [20 mM sodium acetate trihydrate, 10 mM ethylenediaminetetraacetic acid, 40 mM Tris–HCl, and 1% sodium dodecyl sulfate (pH 8.0)] was added and mixed. Phenol–chloroform was used to remove proteins. Total DNA was separated and collected by centrifugation at 11,300*g* for 10 min a Trizol RNA kit (Life Technologies, Carlsbad, CA, USA) was used to extract total RNA according to manufacturer instructions.

### Construction of *P*. *oxalicum* gene-deletion mutants

Deletion of candidate genes from *P*. *oxalicum* was performed according to methods reported by Zhao et al. [16]. The knockout cassette for each candidate gene was constructed by fusion PCR and comprised a 1.9-kb G418-resistance gene and approximately 2 kb of the upstream and downstream DNA fragments of the target gene, which were amplified by PCR using the corresponding primer pairs (Additional file 6: Table S4). The generated knockout cassette was introduced into parental strain ∆*PoxKu70* protoplasts, and selected transformants were further confirmed by PCR and/or Southern blot using specific primer pairs and/or probes (Additional file 6: Table S4).

### Mutant complementation

A complementary strain of the deletion mutant ∆*POX01907* was generated as described previously [21]. The complementary cassette comprised approximately 2 kb of the upstream- and downstream-flanking sequences of an aspartic protease gene (*POX05007*) used as the integrative locus in the genome, 1.2 kb of a DNA fragment encoding the bleomycin-resistance gene, and 7.8 kb of the complementary gene containing the promoter, coding region, and terminator. These four DNA fragments were amplified by PCR using specific primer pairs (Additional file 6: Table S4) and subsequently ligated together using a pEASY-ui seamless cloning and assembly kit (TransGen Biotech, Beijing, China). The generated complementary cassette was introduced into fresh ∆*POX01907* protoplasts, and the resulting complementary strains were further confirmed by PCR.

### Phenotypic investigation of *P. oxalicum* strains

Equal amounts of fresh spores from *P*. *oxalicum* strains, including the deletion mutant ∆*POX01907*, the complementary strain C*POX01907*, and the parental strain ∆*PoxKu70*, were inoculated on solid plates containing glucose or SCS as the sole carbon source or PDA and incubated at 28 °C for 5 days. Colonies were photographed using a Canon EOS 6D digital camera (Canon, Beijing, China).

### Biomass determination for *P. oxalicum* strains

Fresh 1.0 × 10^8^ spores from *P*. *oxalicum* strains, including the parental strain *∆PoxKu70* and the deletion mutant *∆POX01907*, were inoculated into 100 mL of glucose or starch liquid medium, respectively, and cultured at 28 °C for 12 h–72 h. The hypha was harvested using a vacuum filter every 12 h and dried to a constant weight at 50 °C.

### RNA-seq analysis

RNA-seq analysis was performed according to methods described by Zhao et al. [16]. Total RNA extracted from *P*. *oxalicum* strains was used to construct cDNA libraries, with each cDNA having an average length of 100 bp. The constructed cDNA libraries were subjected to evaluation using an Agilent 2100 Bioanalyzer (Agilent Technologies, Santa Clara, CA, USA) and an ABI StepOnePlus real-time PCR system (Applied Biosystems, Forster City, CA, USA), and subsequently sequenced using an IIIumina HiSeq 4000 system (Illumina, San Diego, CA, USA). After quality control, the generated clean reads were mapped onto the *P*. *oxalicum* HP7-1 genome and functionally annotated using BWA v0.7.10-r789 (http://sourceforge.net/projects/bio-bwa/files/) and Bowtie2 v2.1.0 [31]. Gene-expression levels (FPKM) were calculated using RSEM v1.2.12 [22], and DEGs were screened and identified using NOISeq or DESeq2 [23]. Pearson’s correlation coefficient was used to evaluate transcriptome reliability among three biological replicates of each sample.

### Southern hybridization analysis

Southern hybridization analysis of the deletion mutant *∆POX01907* and the parental strain *∆PoxKu70* was performed as previously described [16]. Briefly, total DNA of each strain was extracted and digested with *Pst*I (TaKaRa, Dalian, China). After separation on an 0.8% agarose gel, the generated DNA fragments were transferred to a Hybond-N^+^ nylon membrane (GE Healthcare, Little Chalfont, UK). The probe used for Southern hybridization was amplified with the primers *POX01907*-probe-F and *POX01907*-probe-R (Additional file 4: Table S3). A DIG-High prime DNA labeling and detection starter kit (Life Technologies, Carlsbad, CA, USA) was used to investigate the hybridized bands.

### RT-qPCR analysis

RT-qPCR was used to analyze differences in the expression levels of amylase genes between the deletion mutant ∆*POX01907* and the parental strain ∆*PoxKu70* according to a previously described method [16]. Total RNA from both ∆*POX01907* and ∆*PoxKu70* was extracted and used as a template to generate first-strand cDNA for RT-PCR using the PrimeScript RT regent kit with gDNA Eraser (TaKaRa). Each qPCR comprised a 20-μL volume, including 2.0 μL of the template for first-stand cDNA, 10 μL of SYBR Premix ExTaq II, 0.8 μL of 10 μM primer (either forward or reverse), and 6.4 μL of sterile water, subjected to initial denaturation for 3 min at 98 °C, followed by 40 cycles of 10 s at 98 °C and 30 s at 58 °C. Fluorescent signals were investigated at the end of each extension step at 80 °C according to the method described by Zhang et al. [10].

### Enzyme activity and concentration

RSDE activity and concentration were measured as described previously [5]. Briefly, crude extract from *P*. *oxalicum* strains, including the deletion mutant ∆*POX01907*, the complementary strain C*POX01907*, and the parental strain ∆*PoxKu70*, was added to 0.1 M citric acid/disodium hydrogen phosphate buffer (pH 4.5) containing 1.0% (w/v) raw cassava flour as the substrate, and the mixture was incubated at 65 °C for 30 min. Inactivated crude enzyme extract was used as the blank control. The generated reducing sugars were measured using the 3,5-dinitrosalicylic acid method [32] at 540 nm. One unit of enzymatic activity (U) was defined as the amount of enzyme required to produce 1 μmol of reducing sugars per min from the reaction substrate.

Intracellular enzyme concentration in *P*. *oxalicum* strains was measured using a Bradford assay kit (Pierce Biotechnology, Rockford, IL, USA) according to manufacturer instructions.

### Phylogenetic analysis

POXO1907 homologs were downloaded from the NCBI (https://www.ncbi.nlm.nih.gov/) website following BlastP analyses (https://blast.ncbi.nlm.nih.gov/Blast.cgi?PAGE=Proteins). The phylogenetic tree was constructed using MEGA 7.0 software [33] with a neighbor-joining method and a Poisson correction model.

### Statistical analysis

Microsoft Excel (Office 2016; Microsoft, Redmond, WA, USA) was used for the statistical analysis of all experimental data associated with enzyme production and gene transcription. Significance (*P* < 0.05 or *P* < 0.01) among samples was calculated using Student’s *t* test.

### Accession numbers

All transcriptomic data are available from the SRA database (Accession Number SRP116594). DNA sequences of *POX01907*, *POX03446*, *POX06509*, *POX07078* and *POX09752* are available from the GenBank database (Accession Numbers MH742968-MH742972).

## Additional files

**Additional file 1: Table S1.** Summary of RNA-seq reads obtained for *Penicillium oxalicum* HP7-1.

**Additional file 2: Figure S1.** Pearson’s correlation coefficient of the transcriptomes of *Penicillium oxalicum* HP7-1 among three biological replicates. *P*. *oxalicum* HP7-1 was cultivated in media containing glucose or soluble corn starch for 4 h after a shift from glucose.

**Additional file 3: Table S2.** List of 916 differentially expressed genes in *Penicillium oxalicum* HP7-1 grown in the presence of starch as compared with that in the presence of glucose.

**Additional file 4: Table S3.** List of 23 candidate regulatory genes determined in this study that regulate raw starch-degrading enzymes production in *P. oxalicum* HP7-1.

**Additional file 5: Figure S2.** Confirmation of deletion of 11 candidate genes derived from ∆*PoxKu70* and the complementary strain. **a** PCR analysis. M, 1-kb DNA marker; lanes 1–3, three transformants for each candidate gene; lane 4, ∆*PoxKu70*; and lane 5, ddH_2_O. The top panel shows amplification of the region to the left of the target gene, the middle panel shows amplification of the region to the right of the target gene, and the bottom panel shows amplification of the region of the target gene. **b** Southern hybridization analysis of the deletion mutant ∆*POX01907*. M, 1-kb DNA marker; lane 1, ∆*PoxKu70*; lane 2, ∆*POX01907*-7; lane 3, ∆*POX01907*-9; and lane 4, ∆*POX01907*-15. **c** PCR confirmation of the complementary strain C*POX01907*. M, 1-kb DNA marker; lane 1, C*POX01907*; lane 2, ∆*PoxKu70*; and lane 3, ddH_2_O. The top panel shows amplification of the bleomycin-resistance gene, and the bottom panel shows amplification of complementary cassette.

**Additional file 6: Table S4.** Primers used in this study.

**Additional file 7: Figure S3.** Pearson’s correlation analysis of the transcriptomes of *Penicillium oxalicum* strains ∆*POX01907* and ∆*PoxKu70* grown in medium containing soluble corn starch as the carbon source.

**Additional file 8: Table S5.** List of 1003 genes differentially expressed in ∆*POX01907* as compared with the parental strain ∆*PoxKu70* grown on soluble corn starch as the sole carbon source.

## Abbreviations

CAZymes: carbohydrate-active enzymes
CWDEs: plant cell wall-degrading enzymes
DBD: DNA-binding domain
DEGs: differentially expressed genes
FPKM: fragments per kilobase of exon per million mapped fragments
PDA: potato-dextrose agar
RSDE: raw-starch-digesting enzyme
RSDG: raw-starch-digesting glucoamylase
SCS: soluble corn starch
RT-qPCR: real-time quantitative reverse transcription-PCR
TFs: transcription factors

## References

[1] Sun X, Liu Z, Qu Y, Li X (2008). The effects of wheat bran composition on the production of biomass-hydrolyzing enzymes by *Penicillium decumbens*. Appl Biochem Biotechnol.

[2] Robertson GH, Wong DW, Lee CC, Wagschal K, Smith MR, Orts WJ (2006). Native or raw starch digestion: a key step in energy efficient biorefining of grain. J Agric Food Chem.

[3] Rajoka MI, Yasmeen A (2005). Induction, and production studies of a novel glucoamylase of *Aspergillus niger*. World J Microbiol Biotechnol.

[4] Hata Y, Ishida H, Kojima Y, Ichikawa E, Kawato A, Suginami K, Imayasu S (1997). Comparison of two glucoamylases produced by *Aspergillus oryzae* in solid-state culture (koji) and in submerged culture. J Ferment Bioeng.

[5] Xu QS, Yan YS, Feng JX (2016). Efficient hydrolysis of raw starch and ethanol fermentation: a novel raw starch-digesting glucoamylase from *Penicillium oxalicum*. Biotechnol Biofuels.

[6] Li HF, Chi ZM, Duan XH, Wang L, Sheng J, Wu LF (2007). Glucoamylase production by the marine yeast *Aureobasidium pullulans* N13d and hydrolysis of potato starch granules by the enzyme. Process Biochem.

[7] Fujio Y, Morita H (1996). Improved glucoamylase production by *Rhizopus* sp. A-11 using metal-ion supplemented liquid medium. J Ferment Bioeng.

[8] Ayodeji AO, Ogundolie FA, Bamidele OS, Kolawole AO, Ajele JO (2017). Raw starch degrading, acidic-thermostable glucoamylase from *Aspergillus fumigatus* CFU-01: purification and characterization for biotechnological application. J Microbiol Biotechnol.

[9] Lomthong T, Chotineeranat S, Kitpreechavanich V (2015). Production and characterization of raw starch degrading enzyme from a newly isolated thermophilic filamentous bacterium, *Laceyella sacchari* LP175. Starch-Stärke.

[10] Zhang T, Zhao S, Liao LS, Li CX, Liao GY, Feng JX (2017). Deletion of *TpKu70* facilitates gene targeting in *Talaromyces pinophilus* and identification of *TpAmyR* involvement in amylase production. World J Microbiol Biotechnol.

[11] Xiong Y, Wu VW, Lubbe A, Qin L, Deng SW, Kennedy M, Bauer D, Singan VR, Barry K, Northen TR, Grigoriev IV, Glass NL (2017). A fungal transcription factor essential for starch degradation affects integration of carbon and nitrogen metabolism. PLoS Genet.

[12] Hu YB, Liu GD, Li ZH, Qin YQ, Qu YB, Song X (2013). G protein-cAMP signaling pathway mediated by PGA3 plays different roles in regulating the expressions of amylases and cellulases in *Penicillium decumbens*. Fungal Genet Biol.

[13] Lei YF, Liu GD, Li ZH, Gao LW, Qin YQ, Qu YB (2014). Functional characterization of protein kinase CK2 regulatory subunits regulating *Penicillium oxalicum* asexual development and hydrolytic enzyme production. Fungal Genet Biol.

[14] Chen L, Zou G, Zhang L, de Vries RP, Yan X, Zhang J, Liu R, Wang CS, Qu YB, Zhou ZH (2014). The distinctive regulatory roles of PrtT in the cell metabolism of *Penicillium oxalicum*. Fungal Genet Biol.

[15] Xiong YR, Zhao S, Fu LH, Liao XZ, Li CX, Yan YS, Liao LS, Feng JX (2018). Characterization of novel roles of a HMG-box protein PoxHmbB in biomass-degrading enzyme production by *Penicillium oxalicum*. Appl Microbiol Biotechnol.

[16] Zhao S, Yan YS, He QP, Yang L, Yin X, Li CX, Mao LC, Liao LS, Huang JQ, Xie SB, Nong QD, Zhang Z, Jing L, Xiong YR, Duan CJ, Liu JL, Feng JX (2016). Comparative genomic, transcriptomic and secretomic profiling of *Penicillium oxalicum* HP7-1 and its cellulase and xylanase hyper-producing mutant EU2106, and identification of two novel regulatory genes of cellulase and xylanase gene expression. Biotechnol Biofuels.

[17] Huber W, Carey VJ, Gentleman R, Anders S, Carlson M, Carvalho BS, Bravo HC, Davis S, Gatto L, Girke T, Gottardo R, Hahne F, Hansen KD, Irizarry RA, Lawrence M, Love MI, MacDonald J, Obenchain V, Oleś AK, Pagès H, Reyes A, Shannon P, Smyth GK, Tenenbaum D, Waldron L, Morgan M (2015). Orchestrating high-throughput genomic analysis with bioconductor. Nat Methods.

[18] Lockington RA, Rodbourn L, Barnett S, Carter CJ, Kelly JM (2002). Regulation by carbon and nitrogen sources of a family of cellulases in *Aspergillus nidulans*. Fungal Genet Biol.

[19] Breakspear A, Momany M (2007). *Aspergillus nidulans* conidiation genes *dewA*, *fluG*, and *stuA* are differentially regulated in early vegetative growth. Eukaryot Cell.

[20] Lee BY, Han SY, Choi HG, Kim JH, Han KH, Han DM (2005). Screening of growth- or development-related genes by using genomic library with inducible promoter in *Aspergillus nidulans*. J Microbiol.

[21] Yan YS, Zhao S, Liao LS, He QP, Xiong YR, Wang L, Li CX, Feng JX (2017). Transcriptomic profiling and genetic analyses reveal novel key regulators of cellulase and xylanase gene expression in *Penicillium oxalicum*. Biotechnol Biofuels.

[22] Li B, Dewey CN (2011). RSEM: accurate transcript quantification from RNA-Seq data with or without a reference genome. BMC Bioinform.

[23] Love MI, Huber W, Anders S (2014). Moderated estimation of fold change and dispersion for RNA seq data with DESeq2. Genome Biol.

[24] Boyer LA, Latek RR, Peterson CL (2004). The SANT domain: a unique histone-tail-binding module?. Nat Rev Mol Cell Biol.

[25] Gómez-Zambrano Á, Crevillén P, Franco-Zorrilla JM, López JA, Moreno-Romero J, Roszak P, Santos-González J, Jurado S, Vázquez J, Köhler C, Solano R, Piñeiro M, Jarillo JA (2018). *Arabidopsis* SWC4 binds DNA and recruits the SWR1 complex to modulate histone H2A.Z. Mol Plant.

[26] Ryan DP, Sundaramoorthy R, Martin D, Singh V, Owenughes T (2011). The DNA-binding domain of the Chd1 chromatin-remodelling enzyme contains SANT and SLIDE domains. EMBO J.

[27] Simic R, Lindstrom D, Tran HG, Roinick KL, Costa PJ, Johnson AD, Hartzog GA, Arndt KM (2003). Chromatin remodeling protein Chd1 interacts with transcription elongation factors and localizes to transcribed genes. EMBO J.

[28] Zhang FL, Cao YL, Lv XX, Wang L, Li CY, Zhang WX, Chen GJ, Liu WF (2017). A copper-responsive promoter replacement system to investigate gene functions in *Trichoderma reesei*: a case study in characterizing SAGA genes. Appl Microbiol Biotechnol.

[29] He QP, Zhao S, Wang JX, Li CX, Yan YS, Wang L, Liao LS, Feng JX (2018). Transcription factor PoxNsdD regulates the expression of genes involved in plant biomass-degrading enzymes, conidiation and pigment biosynthesis in *Penicillium oxalicum*. Appl Environ Microbiol.

[30] Li ZH, Yao G, Wu R, Gao L, Kan Q, Liu M, Yang P, Liu G, Qin Y, Song X, Zhong Y, Fang X, Qu Y (2015). Synergistic and dose-controlled regulation of cellulase gene expression in *Penicillium oxalicum*. PLoS Genet.

[31] Langmead B, Salzberg SL (2012). Fast gapped-read alignment with Bowtie 2. Nat Methods.

[32] Miller GL (1959). Use of dinitrosalicylic acid reagent for determination of reducing sugar. Anal Chem.

[33] Kumar S, Stecher G, Tamura K (2016). MEGA7: molecular evolutionary genetics analysis version 7.0 for bigger datasets. Mol Biol Evol..

