# Supplementary material for: Identification of an essential regulator controlling the production of raw-starch-digesting glucoamylase in Penicillium oxalicum
Source: Biotechnol Biofuels. 2019 Jan 4;12:7. doi: 10.1186/s13068-018-1345-z (PMC6318894; doi:10.1186/s13068-018-1345-z)
Supplement: Supplementary file 1 — Additional file 1: Table S1. Summary of RNA-seq reads obtained for Penicillium oxalicum HP7-1. [file 13068_2018_1345_MOESM1_ESM.pdf]

Additional file 1: Table S1. Summary of RNA-sequencing reads obtained for *Penicillium oxalicum* strain HP7-1.

Samples	Clean Reads	Number of nucleotides (bp)	Overall read alignment rate (%)	Concordant pair alignment rate (%)	Number of expressed genes
HP7-1_Glu_1	24678562	2467856200	93.05	78.2	8445
HP7-1_Glu_2	23757736	2375773600	96.35	83.45	7494
HP7-1_Glu_3	23781372	2378137200	96.07	84.15	7660
HP7-1_Starch_1	23609450	2360945000	93.74	80.01	8355
HP7-1_Starch_2	23728260	2372826000	94.19	81.16	8029
HP7-1_Starch_3	23717656	2371765600	94.12	80.18	7895
Δ*PoxKu70*_1	21757564	2175756400	93.09	84.98	8387
Δ*PoxKu70*_2	22081378	2208137800	91.40	86.33	8260
Δ*PoxKu70*_3	22088384	2208838400	93.66	88.53	8382
Δ*POX01907*_1	21640452	2164045200	90.89	83.99	8443
Δ*POX01907*_2	22131274	2213127400	91.40	86.39	8260
Δ*POX01907*_3	22599718	2259971800	93.66	88.67	8382
