# Supplementary figures and images for: Identification of an essential regulator controlling the production of raw-starch-digesting glucoamylase in Penicillium oxalicum
Source: Biotechnol Biofuels. 2019 Jan 4;12:7. doi: 10.1186/s13068-018-1345-z (PMC6318894; doi:10.1186/s13068-018-1345-z)

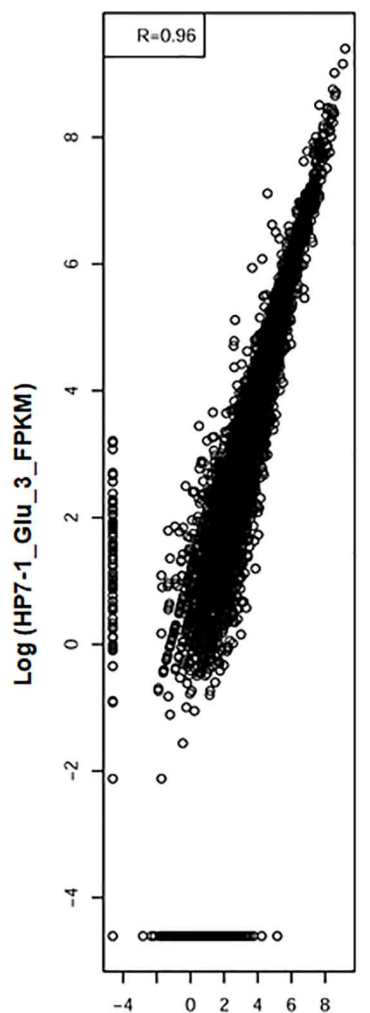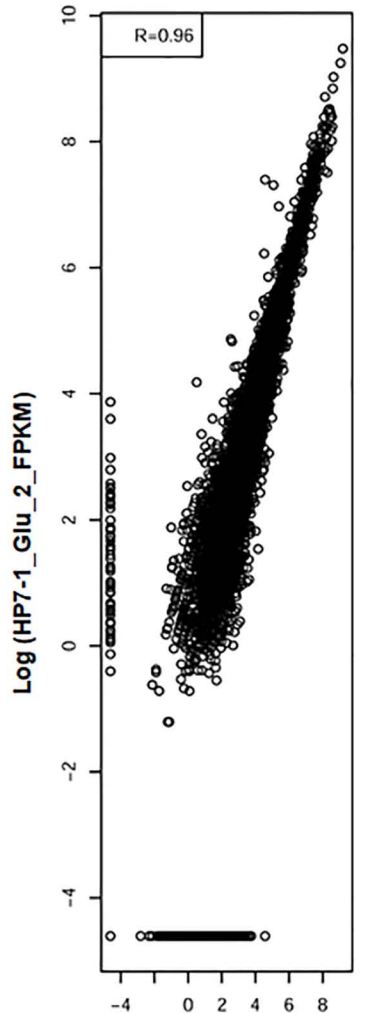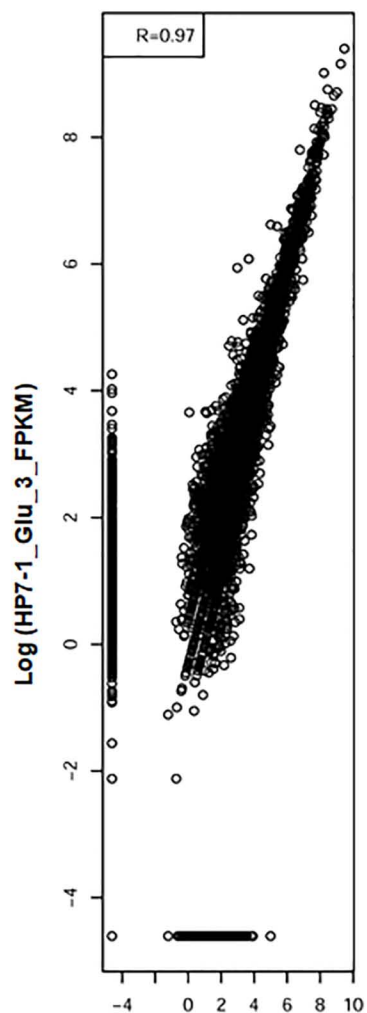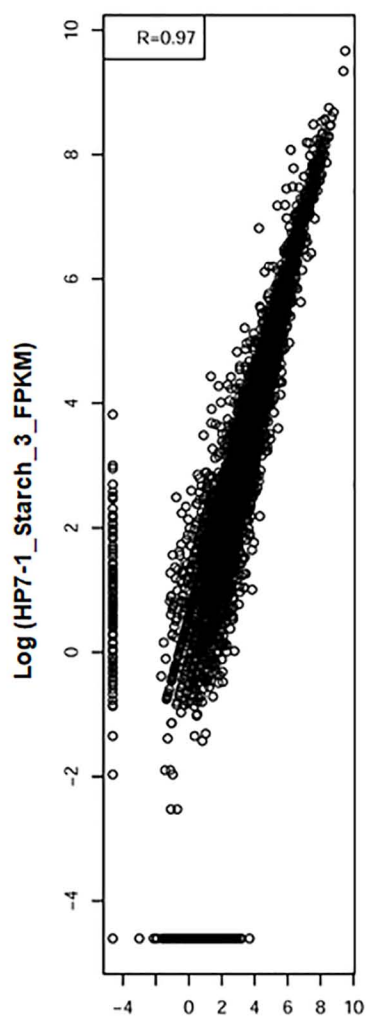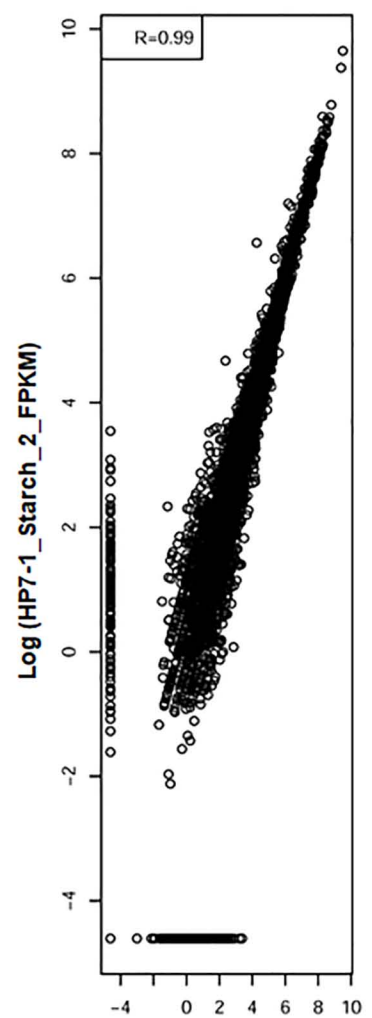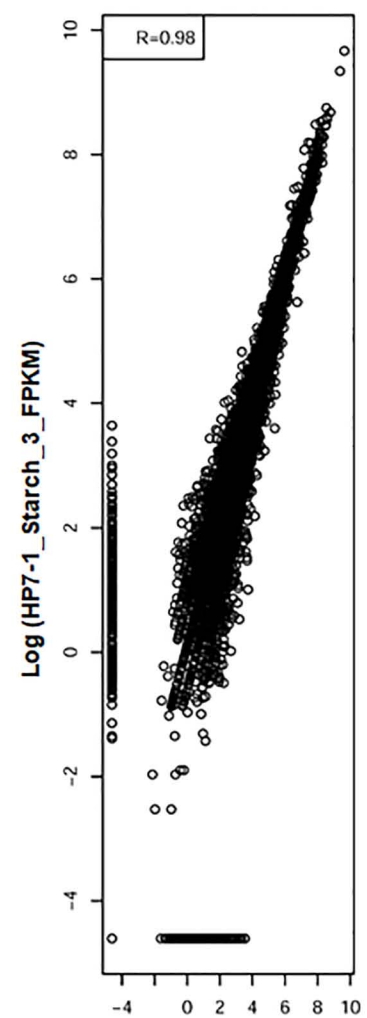

Supplement: Supplementary file 2 — Additional file 2: Figure S1. Pearson’s correlation coefficient of the transcriptomes of Penicillium oxalicum HP7-1 among three biological replicates. P. oxalicum HP7-1 was cultivated in media containing glucose or soluble corn starch for 4 h after a shift from glucose. [file 13068_2018_1345_MOESM2_ESM.pdf]

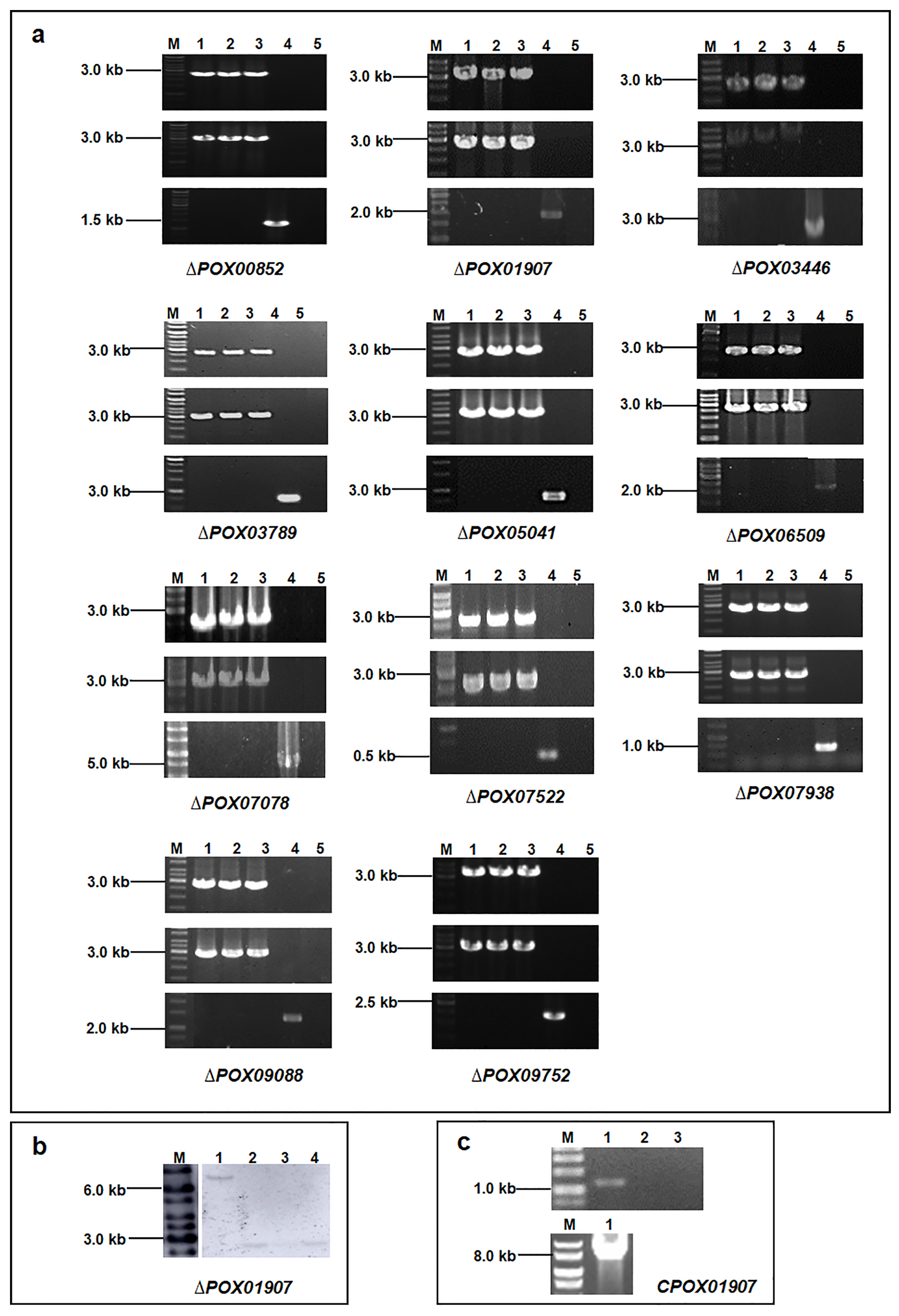

Supplement: Supplementary file 5 — Additional file 5: Figure S2. Confirmation of deletion of 11 candidate genes derived from ∆PoxKu70 and the complementary strain. a PCR analysis. M, 1-kb DNA marker; lanes 1–3, three transformants for each candidate gene; lane 4, ∆PoxKu70; and lane 5, ddH2O. The top panel shows amplification of the region to the left of the target gene, the middle panel shows amplification of the region to the right of the target gene, and the bottom panel shows amplification of the region of the target gene. b Southern hybridization analysis of the deletion mutant ∆POX01907. M, 1-kb DNA marker; lane 1, ∆PoxKu70; lane 2, ∆POX01907-7; lane 3, ∆POX01907-9; and lane 4, ∆POX01907-15. c PCR confirmation of the complementary strain CPOX01907. M, 1-kb DNA marker; lane 1, CPOX01907; lane 2, ∆PoxKu70; and lane 3, ddH2O. The top panel shows amplification of the bleomycin-resistance gene, and the bottom panel shows amplification of complementary cassette. [file 13068_2018_1345_MOESM5_ESM.tif]

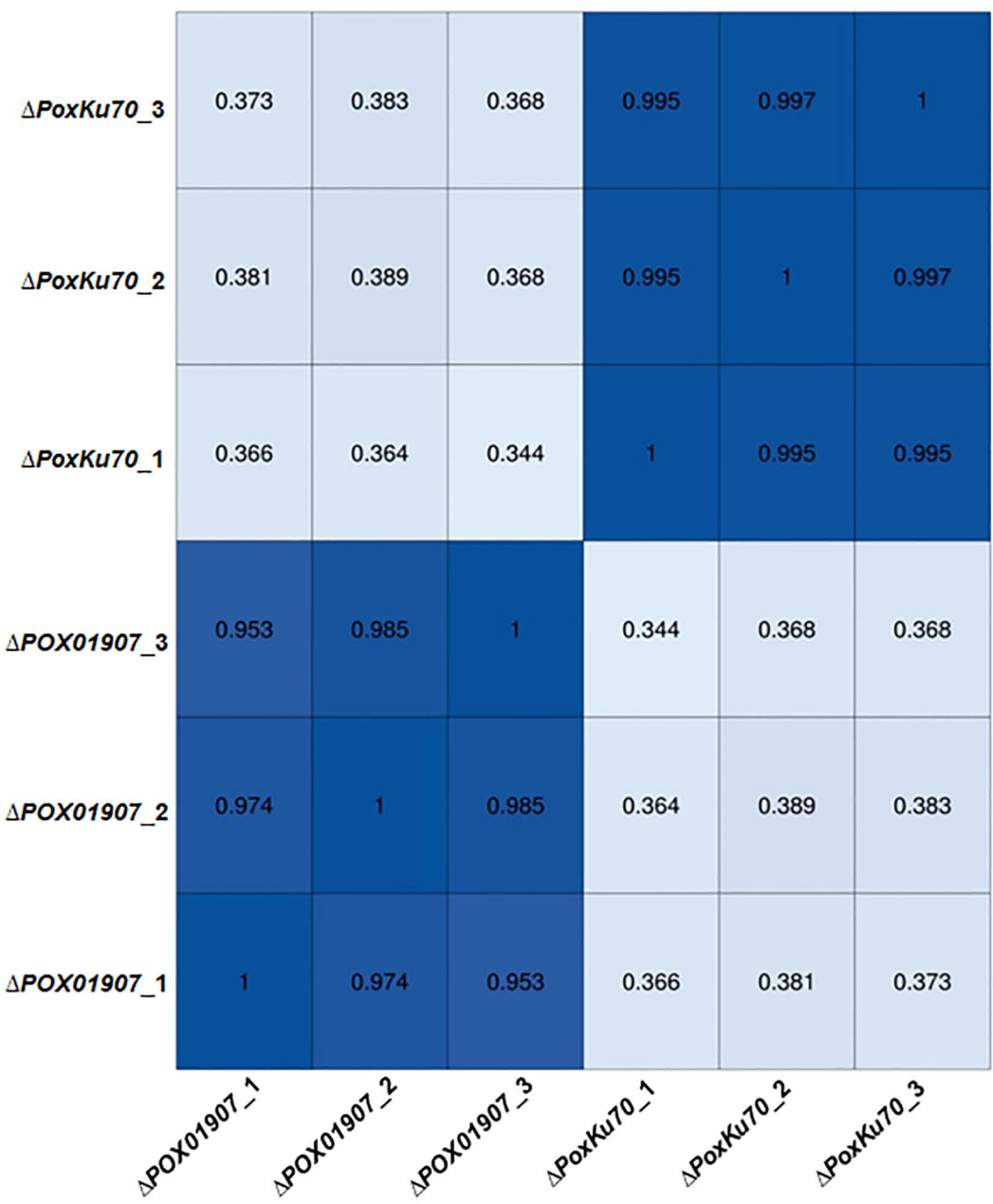

Supplement: Supplementary file 7 — Additional file 7: Figure S3. Pearson’s correlation analysis of the transcriptomes of Penicillium oxalicum strains ∆POX01907 and ∆PoxKu70 grown in medium containing soluble corn starch as the carbon source. [file 13068_2018_1345_MOESM7_ESM.pdf]
