# Supplementary material for: Identification of an essential regulator controlling the production of raw-starch-digesting glucoamylase in Penicillium oxalicum
Source: Biotechnol Biofuels. 2019 Jan 4;12:7. doi: 10.1186/s13068-018-1345-z (PMC6318894; doi:10.1186/s13068-018-1345-z)
Supplement: Supplementary file 3 — Additional file 3: Table S2. List of 916 differentially expressed genes in Penicillium oxalicum HP7-1 grown in the presence of starch as compared with that in the presence of glucose. [file 13068_2018_1345_MOESM3_ESM.pdf]

Additional file 3: Table S2. List of 961 differentially expressed genes in *Penicillium oxalicum* HP7-1 grown in the presence of starch as compared with that in the presence of glucose

Gene ID	Production	CWDEs	CAZy family	Putative Transcription factor	Means-HP7-1_Glu_FPKM	Means-HP7-1_starch_FPKM	Log2 (HP7-1_starch_FPKM/HP7-1_Glu_FPKM)	Up/Down Regulation (HP7-1_starch/HP7-1_glu)	Probability
POX00003	hypothetical protein	NA	NA	NA	47.70	88.25	0.89	Up	0.68
POX00022	hypothetical protein	NA	NA	NA	181.71	369.45	1.02	Up	0.77
POX00023	hypothetical protein	NA	NA	NA	61.85	127.92	1.05	Up	0.74
POX00029	hypothetical protein	NA	NA	NA	204.78	328.63	0.68	Up	0.66
POX00039	hypothetical protein	NA	NA	NA	17.20	66.35	1.95	Up	0.81
POX00040	hypothetical protein	NA	NA	NA	15.27	42.19	1.47	Up	0.71
POX00042	hypothetical protein	NA	NA	NA	19.27	42.25	1.13	Up	0.66
POX00050	hypothetical protein	NA	CE10	NA	5.17	38.86	2.91	Up	0.79
POX00069	hypothetical protein	NA	NA	NA	2209.39	4363.03	0.98	Up	0.78
POX00078	hypothetical protein	NA	NA	NA	280.61	506.84	0.85	Up	0.73
POX00089	hypothetical protein	NA	GH18	NA	55.88	143.24	1.36	Up	0.80
POX00098	hypothetical protein	NA	NA	NA	893.52	1592.26	0.83	Up	0.73
POX00100	hypothetical protein	NA	NA	NA	94.07	151.10	0.68	Up	0.64
POX00103	hypothetical protein	NA	NA	NA	554.84	818.15	0.56	Up	0.61
POX00108	hypothetical protein	NA	NA	NA	1521.22	2827.51	0.89	Up	0.76
POX00109	hypothetical protein	NA	NA	NA	52.99	91.17	0.78	Up	0.64
POX00114	hypothetical protein	NA	NA	NA	38.92	68.58	0.82	Up	0.63
POX00120	hypothetical protein	NA	NA	NA	112.64	175.95	0.64	Up	0.62
POX00121	hypothetical protein	NA	NA	NA	131.15	321.28	1.29	Up	0.82

POX00122	hypothetical protein	NA	NA	NA	869.27	1811.42	1.06	Up	0.79
POX00123	hypothetical protein	NA	NA	NA	57.97	117.06	1.01	Up	0.73
POX00124	hypothetical protein	NA	NA	NA	77.55	143.31	0.89	Up	0.71
POX00138	hypothetical protein	NA	NA	NA	1159.41	1828.21	0.66	Up	0.67
POX00141	hypothetical protein	NA	NA	NA	101.42	159.76	0.66	Up	0.63
POX00146	hypothetical protein	NA	NA	NA	79.61	165.90	1.06	Up	0.76
POX00162	hypothetical protein	NA	NA	NA	121.38	188.37	0.63	Up	0.62
POX00167	NA	NA	NA	NA	666.36	1536.06	1.20	Up	0.82
POX00174	hypothetical protein	NA	NA	NA	47.11	157.44	1.74	Up	0.84
POX00176	hypothetical protein	NA	NA	NA	1810.15	5488.41	1.60	Up	0.86
POX00177	hypothetical protein	NA	NA	NA	41.99	74.05	0.82	Up	0.64
POX00180	hypothetical protein	NA	NA	NA	90.56	151.66	0.74	Up	0.66
POX00186	hypothetical protein	NA	NA	NA	105.74	202.74	0.94	Up	0.74
POX00217	hypothetical protein	NA	NA	NA	957.81	541.06	-0.82	Down	0.73
POX00218	hypothetical protein	NA	NA	NA	22.85	4.71	-2.28	Down	0.68
POX00225	hypothetical protein	NA	NA	NA	80.33	143.11	0.83	Up	0.69
POX00230	hypothetical protein	NA	NA	NA	162.75	266.29	0.71	Up	0.67
POX00231	hypothetical protein	NA	NA	NA	64.72	151.78	1.23	Up	0.79
POX00244	hypothetical protein	NA	NA	NA	10.53	35.95	1.77	Up	0.72
POX00250	hypothetical protein	NA	NA	NA	209.77	324.88	0.63	Up	0.64
POX00257	hypothetical protein	NA	NA	NA	55.99	106.32	0.93	Up	0.71
POX00260	hypothetical protein	NA	NA	NA	32.34	68.70	1.09	Up	0.71
POX00270	hypothetical protein	NA	NA	NA	21.40	43.62	1.03	Up	0.64
POX00292	hypothetical protein	NA	NA	NA	113.56	200.40	0.82	Up	0.70
POX00293	hypothetical protein	NA	NA	NA	1385.86	2125.47	0.62	Up	0.65
POX00296	hypothetical protein	NA	NA	NA	74.08	115.81	0.64	Up	0.60
POX00323	hypothetical protein	NA	NA	NA	63.05	110.22	0.81	Up	0.67
POX00347	hypothetical protein	NA	CBM21	NA	267.96	150.74	-0.83	Down	0.71
POX00348	TPA: Histone H4. 2	NA	NA	NA	108.58	192.42	0.83	Up	0.70
POX00352	hypothetical protein	NA	NA	NA	7.81	29.23	1.90	Up	0.70
POX00356	hypothetical protein	NA	NA	NA	82.63	155.69	0.91	Up	0.72
POX00361	hypothetical protein	NA	NA	NA	166.71	432.62	1.38	Up	0.83
POX00365	hypothetical protein	NA	NA	NA	116.58	210.39	0.85	Up	0.71
POX00383	hypothetical protein	NA	NA	NA	53.01	89.06	0.75	Up	0.63
POX00431	hypothetical protein	NA	NA	NA	55.31	100.42	0.86	Up	0.68
POX00449	hypothetical protein	NA	NA	NA	895.98	603.62	-0.57	Down	0.62
POX00466	hypothetical protein	NA	NA	NA	29.63	67.54	1.19	Up	0.73
POX00470	hypothetical protein	NA	NA	NA	16.77	34.21	1.03	Up	0.60
POX00484	hypothetical protein	NA	NA	NA	35.11	62.93	0.84	Up	0.63

POX00499	hypothetical protein	NA	NA	NA	504.74	978.22	0.95	Up	0.77
POX00504	hypothetical protein	NA	NA	NA	72.35	113.92	0.66	Up	0.61
POX00506	hypothetical protein	NA	NA	NA	27.91	65.93	1.24	Up	0.73
POX00548	hypothetical protein	NA	NA	NA	37.38	80.53	1.11	Up	0.73
POX00549	hypothetical protein	NA	NA	NA	14.10	35.11	1.32	Up	0.66
POX00585	hypothetical protein	NA	NA	NA	67.35	105.63	0.65	Up	0.60
POX00587	hypothetical protein	NA	NA	NA	90.89	145.93	0.68	Up	0.63
POX00590	hypothetical protein	NA	NA	NA	101.72	282.36	1.47	Up	0.83
POX00616	hypothetical protein	NA	NA	NA	52.90	24.12	-1.13	Down	0.69
POX00618	hypothetical protein	NA	NA	NA	262.57	732.17	1.48	Up	0.85
POX00623	hypothetical protein	NA	NA	NA	75.34	133.74	0.83	Up	0.69
POX00640	hypothetical protein	NA	NA	NA	78.71	148.23	0.91	Up	0.72
POX00656	hypothetical protein	NA	NA	NA	255.16	156.49	-0.71	Down	0.67
POX00658	hypothetical protein	NA	NA	NA	58.99	100.30	0.77	Up	0.65
POX00659	hypothetical protein	NA	NA	NA	1644.46	3629.88	1.14	Up	0.81
POX00667	hypothetical protein	NA	NA	NA	487.95	254.62	-0.94	Down	0.76
POX00670	hypothetical protein	NA	NA	NA	121.45	248.40	1.03	Up	0.77
POX00676	hypothetical protein	NA	NA	NA	194.98	120.89	-0.69	Down	0.65
POX00679	NA	NA	NA	NA	7.51	26.30	1.81	Up	0.68
POX00683	NA	NA	NA	NA	0.01	11.53	10.17	Up	0.68
POX00695	hypothetical protein	NA	NA	NA	29.56	75.56	1.35	Up	0.76
POX00702	hypothetical protein	NA	NA	NA	17.95	44.31	1.30	Up	0.70
POX00714	hypothetical protein	NA	NA	NA	20.19	3.10	-2.70	Down	0.68
POX00715	hypothetical protein	NA	NA	NA	131.85	62.53	-1.08	Down	0.75
POX00730	hypothetical protein	NA	NA	NA	59.95	33.78	-0.83	Down	0.62
POX00732	hypothetical protein	NA	NA	NA	3.69	16.67	2.18	Up	0.61
POX00740	hypothetical protein	NA	NA	NA	4.16	19.89	2.26	Up	0.65
POX00741	hypothetical protein	NA	NA	NA	28.56	69.19	1.28	Up	0.74
POX00743	hypothetical protein	NA	NA	NA	17.00	38.79	1.19	Up	0.66
POX00804	hypothetical protein	NA	NA	NA	6.29	21.57	1.78	Up	0.63
POX00805	hypothetical protein	NA	NA	NA	328.98	583.77	0.83	Up	0.73
POX00826	hypothetical protein	NA	NA	NA	98.92	187.91	0.93	Up	0.73
POX00830	hypothetical protein	NA	NA	NA	29.93	72.10	1.27	Up	0.75
POX00835	hypothetical protein	NA	NA	NA	135.89	230.36	0.76	Up	0.69
POX00852	Zinc finger DHHC-type palmitoyltransferase	NA	NA	IPR001594:Zinc finger DHHC-type	14.91	32.88	1.14	Up	0.62

POX00856	hypothetical protein	NA	NA	NA	38.31	66.38	0.79	Up	0.62
POX00865	hypothetical protein	NA	NA	NA	90.62	188.67	1.06	Up	0.76
POX00876	hypothetical protein	NA	NA	NA	102.44	171.60	0.74	Up	0.67
POX00879	hypothetical protein	NA	NA	NA	233.63	90.13	-1.37	Down	0.82
POX00881	hypothetical protein	NA	NA	NA	75.16	33.61	-1.16	Down	0.73
POX00882	hypothetical protein	NA	NA	NA	527.22	332.95	-0.66	Down	0.66
POX00905	hypothetical protein	NA	NA	NA	33.04	14.93	-1.15	Down	0.62
POX00916	hypothetical protein	NA	NA	NA	33.99	82.31	1.28	Up	0.76
POX00917	hypothetical protein	NA	NA	NA	190.02	289.78	0.61	Up	0.62
POX00961	hypothetical protein	NA	NA	NA	277.47	522.59	0.91	Up	0.75
POX00974	hypothetical protein	NA	NA	NA	78.43	133.99	0.77	Up	0.67
POX00976	hypothetical protein	NA	NA	NA	474.64	323.04	-0.56	Down	0.61
POX00981	hypothetical protein	NA	NA	IPR007087:Zinc finger C2H2-type	23.22	43.06	0.89	Up	0.60
POX00987	hypothetical protein	NA	NA	NA	21.58	48.38	1.16	Up	0.68
POX01008	hypothetical protein	NA	NA	NA	330.61	518.40	0.65	Up	0.65
POX01015	hypothetical protein	NA	NA	NA	16.42	3.46	-2.25	Down	0.61
POX01017	hypothetical protein	NA	NA	NA	226.41	147.92	-0.61	Down	0.62
POX01019	hypothetical protein	NA	NA	NA	68.69	40.92	-0.75	Down	0.61
POX01022	hypothetical protein	NA	NA	NA	107.54	205.84	0.94	Up	0.74
POX01024	hypothetical protein	NA	NA	NA	6.40	34.23	2.42	Up	0.76
POX01026	hypothetical protein	NA	NA	NA	17.94	37.15	1.05	Up	0.62
POX01027	hypothetical protein	NA	NA	NA	136.32	293.85	1.11	Up	0.79
POX01031	hypothetical protein	NA	NA	NA	114.23	195.64	0.78	Up	0.69
POX01033	hypothetical protein	NA	NA	NA	547.95	997.75	0.86	Up	0.74
POX01041	hypothetical protein	NA	NA	NA	9.35	26.23	1.49	Up	0.64
POX01061	hypothetical protein	NA	NA	NA	19.63	45.97	1.23	Up	0.69
POX01067	hypothetical protein	NA	NA	NA	110.36	181.08	0.71	Up	0.66
POX01074	hypothetical protein	NA	NA	NA	383.21	212.16	-0.85	Down	0.73
POX01087	hypothetical protein	NA	NA	NA	98.90	181.36	0.87	Up	0.72
POX01113	hypothetical protein	NA	NA	NA	1098.30	1822.66	0.73	Up	0.70
POX01115	hypothetical protein	NA	NA	NA	93.23	143.18	0.62	Up	0.60
POX01134	hypothetical protein	NA	NA	NA	860.66	1285.96	0.58	Up	0.63
POX01193	NA	NA	NA	NA	12.94	34.65	1.42	Up	0.68
POX01201	hypothetical protein	NA	NA	NA	11.46	34.48	1.59	Up	0.70
POX01211	hypothetical protein	NA	NA	NA	38.24	3.29	-3.54	Down	0.80
POX01239	hypothetical protein	NA	NA	NA	60.65	154.71	1.35	Up	0.80

POX01268	hypothetical protein	NA	NA	NA	212.51	316.26	0.57	Up	0.60
POX01286	hypothetical protein	NA	NA	NA	323.29	205.49	-0.65	Down	0.65
POX01287	hypothetical protein	NA	NA	NA	355.95	224.68	-0.66	Down	0.65
POX01301	hypothetical protein	NA	NA	NA	1583.00	2447.82	0.63	Up	0.66
POX01304	hypothetical protein	NA	NA	NA	30.82	57.84	0.91	Up	0.64
POX01307	hypothetical protein	NA	NA	NA	56.35	91.31	0.70	Up	0.61
POX01317	hypothetical protein	NA	NA	NA	338.07	1837.19	2.44	Up	0.90
POX01322	hypothetical protein	NA	NA	NA	8.31	23.22	1.48	Up	0.61
POX01332	hypothetical protein	NA	NA	NA	52.91	29.31	-0.85	Down	0.61
POX01336	vacuolar serine protease PrtB	NA	NA	NA	887.05	524.18	-0.76	Down	0.71
POX01339	hypothetical protein	NA	NA	NA	573.31	1002.27	0.81	Up	0.73
POX01342	hypothetical protein	NA	NA	NA	32.81	86.55	1.40	Up	0.78
POX01356	hypothetical protein	NA	CBM20;GH15	NA	13.24	130.99	3.31	Up	0.88
POX01369	hypothetical protein	NA	GT41	NA	265.86	135.00	-0.98	Down	0.76
POX01376	hypothetical protein	NA	NA	NA	10.08	31.76	1.66	Up	0.69
POX01399	hypothetical protein	NA	NA	NA	53.56	98.58	0.88	Up	0.69
POX01406	hypothetical protein	NA	NA	NA	52.44	84.67	0.69	Up	0.60
POX01407	hypothetical protein	NA	NA	NA	106.37	182.76	0.78	Up	0.68
POX01419	hypothetical protein	NA	NA	NA	333.95	216.78	-0.62	Down	0.63
POX01422	hypothetical protein	NA	NA	NA	29.39	6.97	-2.08	Down	0.71
POX01443	hypothetical protein	NA	NA	NA	2473.36	3956.72	0.68	Up	0.68
POX01446	hypothetical protein	NA	NA	NA	111.46	245.63	1.14	Up	0.79
POX01462	hypothetical protein	NA	NA	NA	52.27	26.73	-0.97	Down	0.65
POX01484	hypothetical protein	NA	NA	NA	52.55	172.44	1.71	Up	0.84
POX01491	hypothetical protein	NA	NA	NA	61.75	129.77	1.07	Up	0.75
POX01519	hypothetical protein	NA	NA	NA	11.12	32.60	1.55	Up	0.69
POX01524	hypothetical protein	Expansin-like	CBM63	NA	147.27	86.35	-0.77	Down	0.67
POX01547	hypothetical protein	NA	NA	NA	296.66	553.61	0.90	Up	0.75
POX01549	hypothetical protein	NA	NA	NA	100.50	169.90	0.76	Up	0.67
POX01554	hypothetical protein	NA	NA	NA	39.89	92.95	1.22	Up	0.76
POX01568	hypothetical protein	NA	NA	NA	75.02	44.56	-0.75	Down	0.62
POX01583	hypothetical protein	NA	NA	NA	107.28	297.12	1.47	Up	0.84
POX01597	hypothetical protein	NA	NA	NA	253.66	133.31	-0.93	Down	0.74
POX01608	hypothetical protein	NA	NA	NA	12.75	32.11	1.33	Up	0.65

POX01614	hypothetical protein	NA	NA	NA	42.65	21.26	-1.00	Down	0.63
POX01617	hypothetical protein	NA	NA	NA	128.14	205.81	0.68	Up	0.65
POX01626	hypothetical protein	NA	NA	NA	38.80	109.70	1.50	Up	0.81
POX01630	hypothetical protein	NA	NA	NA	69.27	119.36	0.79	Up	0.67
POX01636	hypothetical protein	NA	NA	NA	12.73	34.25	1.43	Up	0.68
POX01639	hypothetical protein	NA	NA	NA	11.69	45.83	1.97	Up	0.77
POX01651	hypothetical protein	NA	NA	NA	644.89	328.56	-0.97	Down	0.77
POX01660	hypothetical protein	NA	NA	NA	24.88	47.59	0.94	Up	0.63
POX01664	hypothetical protein	NA	NA	NA	971.67	1960.00	1.01	Up	0.78
POX01688	hypothetical protein	NA	NA	NA	265.43	505.96	0.93	Up	0.76
POX01704	hypothetical protein	NA	NA	NA	32.11	63.78	0.99	Up	0.68
POX01715	hypothetical protein	NA	NA	NA	20.19	53.36	1.40	Up	0.73
POX01738	hypothetical protein	NA	NA	NA	29.51	65.19	1.14	Up	0.71
POX01742	hypothetical protein	NA	NA	NA	44.36	78.69	0.83	Up	0.65
POX01751	hypothetical protein	NA	NA	NA	26.63	49.62	0.90	Up	0.62
POX01754	hypothetical protein	NA	NA	NA	41.44	69.11	0.74	Up	0.60
POX01760	hypothetical protein	NA	NA	NA	128.02	216.98	0.76	Up	0.68
POX01761	hypothetical protein	NA	NA	NA	232.84	367.34	0.66	Up	0.65
POX01763	hypothetical protein	NA	NA	NA	57.16	165.52	1.53	Up	0.83
POX01775	hypothetical protein	NA	NA	NA	77.96	125.80	0.69	Up	0.63
POX01777	hypothetical protein	NA	NA	NA	148.10	349.00	1.24	Up	0.81
POX01781	hypothetical protein	NA	NA	NA	79.07	124.43	0.65	Up	0.61
POX01793	hypothetical protein	NA	NA	NA	34.33	66.33	0.95	Up	0.67
POX01812	hypothetical protein	NA	NA	NA	19.55	4.81	-2.02	Down	0.63
POX01826	hypothetical protein	NA	NA	NA	213.67	98.18	-1.12	Down	0.78
POX01829	hypothetical protein	NA	NA	NA	38.86	99.74	1.36	Up	0.78
POX01834	hypothetical protein	NA	NA	NA	234.46	368.64	0.65	Up	0.65
POX01860	hypothetical protein	NA	NA	NA	35.22	67.84	0.95	Up	0.67
POX01870	hypothetical protein	NA	NA	NA	62.34	125.37	1.01	Up	0.74
POX01877	hypothetical protein	NA	NA	NA	73.35	42.09	-0.80	Down	0.63
POX01880	hypothetical protein	NA	NA	NA	240.76	377.30	0.65	Up	0.65
POX01886	hypothetical protein	NA	NA	NA	35.55	81.82	1.20	Up	0.75
POX01889	hypothetical protein	NA	NA	NA	21.90	43.13	0.98	Up	0.63
POX01891	hypothetical protein	NA	NA	NA	125.69	219.95	0.81	Up	0.70

POX01907	hypothetical protein	NA	NA	IPR001005:Myb DNA-binding//IPR009057:Homeodomain-like	61.04	30.94	-0.98	Down	0.67
POX01910	hypothetical protein	NA	NA	NA	63.67	103.47	0.70	Up	0.62
POX01912	hypothetical protein	NA	NA	NA	0.77	13.71	4.15	Up	0.62
POX01920	hypothetical protein	NA	NA	NA	5.08	30.61	2.59	Up	0.75
POX01929	hypothetical protein	NA	NA	NA	1.30	30.06	4.54	Up	0.77
POX01976	hypothetical protein	NA	NA	NA	1319.31	2079.31	0.66	Up	0.67
POX01983	hypothetical protein	NA	NA	NA	66.55	104.99	0.66	Up	0.60
POX01986	hypothetical protein	NA	AA7	NA	162.96	282.98	0.80	Up	0.70
POX01987	hypothetical protein	NA	NA	NA	39.84	95.86	1.27	Up	0.77
POX02008	hypothetical protein	NA	NA	NA	4475.06	2222.69	-1.01	Down	0.78
POX02020	hypothetical protein	NA	NA	NA	17.27	0.22	-6.32	Down	0.70
POX02023	hypothetical protein	NA	NA	NA	353.29	547.78	0.63	Up	0.64
POX02026	hypothetical protein	NA	NA	NA	43.54	210.13	2.27	Up	0.87
POX02033	hypothetical protein	NA	NA	NA	115.61	66.89	-0.79	Down	0.66
POX02047	hypothetical protein	NA	NA	NA	20.17	53.17	1.40	Up	0.73
POX02054	NA	NA	NA	NA	183.02	21.56	-3.09	Down	0.89
POX02055	hypothetical protein	NA	NA	NA	49.03	1.19	-5.36	Down	0.84
POX02066	hypothetical protein	NA	NA	NA	110.50	60.51	-0.87	Down	0.69
POX02069	hypothetical protein	NA	NA	NA	247.45	115.42	-1.10	Down	0.78
POX02082	hypothetical protein	NA	NA	NA	1587.83	2501.09	0.66	Up	0.67
POX02099	hypothetical protein	NA	NA	NA	409.65	611.62	0.58	Up	0.62
POX02111	hypothetical protein	NA	NA	NA	52.04	24.14	-1.11	Down	0.68
POX02121	hypothetical protein	NA	NA	NA	74.13	30.34	-1.29	Down	0.75
POX02123	hypothetical protein	NA	NA	NA	45.04	20.36	-1.15	Down	0.67
POX02126	hypothetical protein	NA	AA4;AA7	NA	45.94	87.13	0.92	Up	0.69
POX02137	hypothetical protein	NA	NA	NA	745.76	501.19	-0.57	Down	0.62
POX02152	hypothetical protein	NA	NA	NA	75.89	182.46	1.27	Up	0.80
POX02183	hypothetical protein	NA	NA	NA	60.12	112.59	0.91	Up	0.70
POX02195	hypothetical protein	NA	NA	NA	57.95	213.35	1.88	Up	0.86
POX02197	hypothetical protein	NA	NA	NA	23.98	8.84	-1.44	Down	0.61
POX02211	hypothetical protein	NA	NA	NA	39.82	70.27	0.82	Up	0.63
POX02217	hypothetical protein	NA	NA	NA	24.77	45.19	0.87	Up	0.60
POX02219	hypothetical protein	NA	NA	NA	89.82	169.25	0.91	Up	0.72
POX02223	hypothetical protein	NA	NA	NA	78.29	31.90	-1.30	Down	0.76

POX02243	hypothetical protein	NA	NA	NA	416.95	241.50	-0.79	Down	0.71
POX02249	hypothetical protein	NA	NA	NA	10.10	31.76	1.65	Up	0.69
POX02252	hypothetical protein	NA	NA	NA	59.79	110.91	0.89	Up	0.70
POX02254	hypothetical protein	NA	NA	NA	56.81	27.69	-1.04	Down	0.68
POX02264	hypothetical protein	NA	NA	NA	1442.36	2460.77	0.77	Up	0.72
POX02286	hypothetical protein	NA	NA	NA	801.57	506.83	-0.66	Down	0.66
POX02290	hypothetical protein	NA	NA	IPR009057:Homeo domain-like	2040.98	953.27	-1.10	Down	0.80
POX02292	hypothetical protein	NA	NA	NA	1366.74	2497.10	0.87	Up	0.75
POX02306	hypothetical protein	NA	NA	NA	761.68	1744.23	1.20	Up	0.82
POX02336	hypothetical protein	NA	NA	NA	85.16	160.53	0.91	Up	0.72
POX02340	hypothetical protein	NA	NA	NA	29.44	57.55	0.97	Up	0.66
POX02372	hypothetical protein	NA	NA	NA	131.41	282.30	1.10	Up	0.78
POX02383	NA	NA	NA	NA	38.04	19.01	-1.00	Down	0.61
POX02395	hypothetical protein	NA	NA	NA	211.99	133.01	-0.67	Down	0.64
POX02405	hypothetical protein	NA	NA	NA	41.55	109.09	1.39	Up	0.79
POX02411	hypothetical protein	NA	NA	NA	40.42	104.24	1.37	Up	0.79
POX02412	hypothetical protein	NA	CBM20;GH15	NA	7.49	56.51	2.91	Up	0.83
POX02432	hypothetical protein	NA	NA	NA	27.78	66.19	1.25	Up	0.74
POX02436	hypothetical protein	NA	NA	NA	9.81	27.15	1.47	Up	0.64
POX02446	hypothetical protein	NA	NA	NA	207.63	320.16	0.62	Up	0.63
POX02452	hypothetical protein	NA	NA	IPR007087:Zinc finger C2H2-type	371.39	212.41	-0.81	Down	0.71
POX02472	hypothetical protein	NA	NA	NA	52.10	7.20	-2.85	Down	0.82
POX02491	hypothetical protein	NA	NA	NA	21.75	43.42	1.00	Up	0.63
POX02497	hypothetical protein	NA	NA	NA	11.89	36.31	1.61	Up	0.71
POX02503	hypothetical protein	NA	NA	NA	131.39	223.61	0.77	Up	0.69
POX02508	hypothetical protein	NA	NA	NA	112.59	187.41	0.74	Up	0.67
POX02510	hypothetical protein	NA	NA	NA	30.98	59.07	0.93	Up	0.65
POX02511	hypothetical protein	NA	NA	NA	54.09	113.24	1.07	Up	0.74
POX02516	hypothetical protein	NA	NA	NA	95.23	172.95	0.86	Up	0.71
POX02520	hypothetical protein	NA	NA	NA	218.85	343.96	0.65	Up	0.65
POX02529	hypothetical protein	NA	NA	NA	47.32	78.78	0.74	Up	0.62
POX02551	hypothetical protein	NA	NA	NA	94.88	149.98	0.66	Up	0.63
POX02557	hypothetical protein	NA	NA	NA	41.51	69.84	0.75	Up	0.61
POX02568	hypothetical protein	NA	NA	NA	152.30	388.88	1.35	Up	0.83

POX02584	hypothetical protein	NA	NA	NA	131.33	199.74	0.60	Up	0.61
POX02589	hypothetical protein	NA	NA	NA	34.18	16.44	-1.06	Down	0.61
POX02605	hypothetical protein	NA	NA	NA	146.47	13.19	-3.47	Down	0.88
POX02608	hypothetical protein	NA	NA	NA	72.55	43.70	-0.73	Down	0.61
POX02615	hypothetical protein	NA	NA	NA	41.18	109.99	1.42	Up	0.80
POX02616	hypothetical protein	NA	NA	NA	169.16	273.63	0.69	Up	0.66
POX02618	hypothetical protein	NA	NA	NA	1225.77	3133.36	1.35	Up	0.84
POX02628	hypothetical protein	NA	NA	NA	705.75	392.55	-0.85	Down	0.74
POX02633	hypothetical protein	NA	NA	NA	16.03	47.14	1.56	Up	0.74
POX02640	hypothetical protein	NA	NA	NA	962.65	1949.91	1.02	Up	0.79
POX02649	hypothetical protein	NA	NA	NA	63.91	23.10	-1.47	Down	0.76
POX02670	hypothetical protein	NA	NA	NA	39.84	75.02	0.91	Up	0.67
POX02680	hypothetical protein	NA	NA	NA	786.59	1494.74	0.93	Up	0.77
POX02691	hypothetical protein	NA	NA	NA	72.83	14.34	-2.34	Down	0.83
POX02700	hypothetical protein	NA	NA	NA	87.81	47.03	-0.90	Down	0.68
POX02724	hypothetical protein	NA	NA	NA	15.96	35.89	1.17	Up	0.64
POX02730	hypothetical protein	NA	NA	NA	1493.39	2699.22	0.85	Up	0.74
POX02745	hypothetical protein	NA	NA	NA	46.52	18.28	-1.35	Down	0.71
POX02774	hypothetical protein	NA	NA	NA	33.67	58.16	0.79	Up	0.60
POX02787	hypothetical protein	NA	NA	NA	1643.39	2719.10	0.73	Up	0.70
POX02791	hypothetical protein	NA	NA	NA	118.73	220.96	0.90	Up	0.73
POX02811	hypothetical protein	NA	NA	NA	20.35	44.32	1.12	Up	0.66
POX02822	hypothetical protein	NA	NA	NA	167.95	266.27	0.66	Up	0.65
POX02846	hypothetical protein	NA	NA	NA	31.53	6.76	-2.22	Down	0.74
POX02853	NA	NA	NA	NA	87.68	177.50	1.02	Up	0.75
POX02873	hypothetical protein	NA	NA	NA	40.84	19.45	-1.07	Down	0.64
POX02884	hypothetical protein	NA	NA	NA	30.03	58.90	0.97	Up	0.66
POX02888	hypothetical protein	NA	NA	NA	45.95	100.36	1.13	Up	0.75
POX02896	hypothetical protein	NA	NA	NA	388.99	703.09	0.85	Up	0.74
POX02897	hypothetical protein	NA	NA	NA	108.64	63.69	-0.77	Down	0.65
POX02901	hypothetical protein	NA	NA	NA	86.95	160.73	0.89	Up	0.72
POX02905	hypothetical protein	NA	NA	NA	25.12	56.23	1.16	Up	0.70
POX02914	hypothetical protein	NA	NA	NA	22.53	4.22	-2.42	Down	0.69
POX02918	hypothetical protein	NA	NA	NA	205.29	424.53	1.05	Up	0.78
POX02926	hypothetical protein	NA	NA	NA	68.09	140.82	1.05	Up	0.75

POX02944	hypothetical protein	NA	NA	IPR001138:Fungal transcriptional regulatory protein N-terminal	60.29	113.10	0.91	Up	0.70
POX02953	hypothetical protein	NA	NA	NA	128.03	204.40	0.67	Up	0.64
POX02972	hypothetical protein	NA	NA	NA	80.63	215.21	1.42	Up	0.82
POX02974	hypothetical protein	NA	NA	NA	11.90	27.71	1.22	Up	0.61
POX02982	hypothetical protein	NA	NA	NA	113.92	179.45	0.66	Up	0.63
POX02986	hypothetical protein	NA	NA	NA	50.95	89.14	0.81	Up	0.65
POX03005	putative cutinase	NA	CE5	NA	1751.91	847.85	-1.05	Down	0.79
POX03019	hypothetical protein	NA	NA	NA	331.69	499.00	0.59	Up	0.62
POX03021	hypothetical protein	NA	GH18	NA	59.51	106.21	0.84	Up	0.68
POX03022	hypothetical protein	NA	NA	NA	3068.56	4953.14	0.69	Up	0.69
POX03026	hypothetical protein	NA	NA	NA	85.73	50.23	-0.77	Down	0.64
POX03045	hypothetical protein	NA	NA	NA	50.96	87.19	0.77	Up	0.64
POX03083	hypothetical protein	NA	NA	NA	211.84	370.70	0.81	Up	0.71
POX03089	hypothetical protein	NA	NA	NA	744.46	1592.13	1.10	Up	0.80
POX03093	hypothetical protein	NA	NA	NA	328.24	71.64	-2.20	Down	0.88
POX03095	hypothetical protein	NA	NA	NA	2741.20	942.18	-1.54	Down	0.86
POX03096	hypothetical protein	NA	NA	NA	401.72	62.42	-2.69	Down	0.89
POX03101	hypothetical protein	NA	NA	NA	76.29	121.41	0.67	Up	0.62
POX03104	hypothetical protein	NA	NA	NA	32.48	68.36	1.07	Up	0.70
POX03129	hypothetical protein	NA	NA	NA	176.12	404.32	1.20	Up	0.81
POX03163	hypothetical protein	NA	NA	NA	40.14	14.36	-1.48	Down	0.71
POX03174	hypothetical protein	NA	NA	NA	7.02	21.01	1.58	Up	0.61
POX03179	hypothetical protein	NA	NA	NA	47.93	78.16	0.71	Up	0.60
POX03182	hypothetical protein	NA	NA	NA	35.22	64.81	0.88	Up	0.65
POX03183	hypothetical protein	NA	NA	NA	144.36	83.43	-0.79	Down	0.68
POX03195	hypothetical protein	NA	NA	NA	85.46	180.50	1.08	Up	0.77
POX03202	hypothetical protein	NA	NA	NA	56.51	119.39	1.08	Up	0.75
POX03211	hypothetical protein	NA	NA	NA	34.63	67.32	0.96	Up	0.67
POX03212	hypothetical protein	NA	NA	NA	25.96	52.02	1.00	Up	0.66
POX03227	hypothetical protein	NA	NA	NA	38.41	89.38	1.22	Up	0.76

POX03228	putative alpha-1` 3-glucan synthase	NA	GH13;GT4; GT5	NA	142.28	77.89	-0.87	Down	0.71
POX03270	Mitochondrial carrier protein	NA	NA	NA	19.67	39.99	1.02	Up	0.62
POX03279	hypothetical protein	NA	NA	NA	26.14	6.96	-1.91	Down	0.68
POX03292	hypothetical protein	NA	NA	NA	138.31	61.92	-1.16	Down	0.77
POX03301	hypothetical protein	NA	NA	NA	48.31	91.48	0.92	Up	0.69
POX03308	hypothetical protein	NA	NA	NA	113.95	202.93	0.83	Up	0.70
POX03310	hypothetical protein	NA	NA	NA	23.73	93.69	1.98	Up	0.83
POX03327	hypothetical protein	NA	CE10	NA	49.66	81.58	0.72	Up	0.61
POX03332	hypothetical protein	NA	NA	NA	113.43	71.26	-0.67	Down	0.61
POX03339	hypothetical protein	NA	NA	NA	71.84	149.90	1.06	Up	0.75
POX03341	hypothetical protein	NA	NA	NA	68.66	119.06	0.79	Up	0.67
POX03344	hypothetical protein	NA	NA	NA	15.67	43.88	1.49	Up	0.72
POX03347	hypothetical protein	NA	NA	NA	60.87	100.58	0.72	Up	0.63
POX03361	hypothetical protein	NA	NA	NA	36.08	70.24	0.96	Up	0.68
POX03369	hypothetical protein	NA	NA	NA	140.59	52.08	-1.43	Down	0.81
POX03417	hypothetical protein	NA	NA	NA	89.80	173.47	0.95	Up	0.74
POX03429	hypothetical protein	NA	NA	NA	29.41	68.48	1.22	Up	0.73
POX03443	hypothetical protein	NA	NA	NA	173.87	261.89	0.59	Up	0.61
POX03446	hypothetical protein	NA	NA	IPR001138:Fungal transcriptional regulatory protein N-terminal//IPR007219:Fungal specific transcription factor	11.47	28.95	1.34	Up	0.63
POX03449	hypothetical protein	NA	NA	NA	13.76	30.85	1.16	Up	0.61
POX03473	hypothetical protein	NA	NA	NA	175.34	91.42	-0.94	Down	0.73
POX03477	hypothetical protein	NA	NA	NA	100.42	155.19	0.63	Up	0.61
POX03483	hypothetical protein	NA	NA	NA	438.22	249.94	-0.81	Down	0.72
POX03490	hypothetical protein	NA	GT90	NA	11.69	30.11	1.37	Up	0.65
POX03531	hypothetical protein	NA	NA	NA	34.35	82.44	1.26	Up	0.76
POX03532	hypothetical protein	NA	NA	NA	0.87	19.59	4.50	Up	0.70

POX03560	hypothetical protein	NA	NA	NA	376.66	561.33	0.58	Up	0.62
POX03564	hypothetical protein	NA	NA	NA	18.41	64.32	1.80	Up	0.80
POX03570	hypothetical protein	NA	NA	NA	502.30	990.02	0.98	Up	0.77
POX03571	hypothetical protein	NA	NA	NA	1798.06	2587.89	0.53	Up	0.60
POX03594	hypothetical protein	NA	NA	NA	58.37	15.72	-1.89	Down	0.79
POX03603	hypothetical protein	NA	NA	NA	80.24	159.80	0.99	Up	0.74
POX03605	hypothetical protein	NA	NA	NA	26.68	62.97	1.24	Up	0.73
POX03629	hypothetical protein	NA	NA	NA	122.52	69.12	-0.83	Down	0.68
POX03674	hypothetical protein	NA	NA	NA	173.62	259.60	0.58	Up	0.60
POX03687	hypothetical protein	NA	NA	NA	29.94	63.29	1.08	Up	0.70
POX03696	hypothetical protein	NA	NA	NA	101.57	155.78	0.62	Up	0.61
POX03706	hypothetical protein	NA	NA	NA	116.42	20.53	-2.50	Down	0.86
POX03718	hypothetical protein	NA	NA	NA	41.24	20.76	-0.99	Down	0.62
POX03738	putative endo-alpha-1,4-polygalactosaminidase	NA	GH114	NA	40.52	78.54	0.95	Up	0.69
POX03741	putative alpha-glucosidase	NA	GH13	NA	9.37	306.84	5.03	Up	0.90
POX03742	hypothetical protein	NA	NA	NA	5.74	51.64	3.17	Up	0.82
POX03746	hypothetical protein	NA	NA	NA	12.29	28.19	1.20	Up	0.61
POX03758	hypothetical protein	NA	NA	NA	757.13	1652.63	1.13	Up	0.81
POX03762	hypothetical protein	NA	AA8	NA	8.28	45.20	2.45	Up	0.79
POX03771	NA	NA	NA	NA	72.03	37.76	-0.93	Down	0.67
POX03772	hypothetical protein	NA	NA	NA	14.76	34.12	1.21	Up	0.64
POX03782	hypothetical protein	NA	NA	NA	16.85	37.24	1.14	Up	0.64
POX03784	hypothetical protein	NA	NA	NA	1607.91	3180.28	0.98	Up	0.78
POX03789	hypothetical protein	NA	NA	IPR003163:DNA-binding yeast	332.17	188.30	-0.82	Down	0.71
POX03805	hypothetical protein	NA	NA	NA	29.54	12.30	-1.26	Down	0.62
POX03806	hypothetical protein	NA	NA	NA	67.58	181.51	1.43	Up	0.82
POX03818	hypothetical protein	NA	NA	NA	23.67	58.15	1.30	Up	0.73
POX03823	hypothetical protein	NA	NA	NA	1459.15	2184.34	0.58	Up	0.63

POX03827	hypothetical protein	NA	NA	IPR009044:ssDNA-binding transcriptional regulator	29.80	55.69	0.90	Up	0.64
POX03832	hypothetical protein	NA	NA	NA	63.30	120.17	0.92	Up	0.71
POX03842	NA	NA	NA	NA	35.08	6.35	-2.47	Down	0.76
POX03847	hypothetical protein	NA	NA	NA	65.19	107.05	0.72	Up	0.63
POX03850	hypothetical protein	NA	NA	NA	92.36	158.56	0.78	Up	0.68
POX03862	hypothetical protein	NA	NA	NA	141.19	259.83	0.88	Up	0.73
POX03882	hypothetical protein	NA	NA	NA	185.03	305.17	0.72	Up	0.68
POX03889	putative alpha-glucosidase	NA	GH31	NA	86.16	384.02	2.16	Up	0.88
POX03937	hypothetical protein	NA	NA	NA	828.90	1397.92	0.75	Up	0.71
POX03941	hypothetical protein	NA	NA	NA	139.86	219.92	0.65	Up	0.64
POX03951	hypothetical protein	NA	NA	NA	74.08	124.02	0.74	Up	0.65
POX03972	hypothetical protein	NA	NA	NA	59.97	117.83	0.97	Up	0.72
POX03988	hypothetical protein	NA	NA	NA	495.41	744.20	0.59	Up	0.63
POX03996	hypothetical protein	NA	NA	NA	30.76	61.29	0.99	Up	0.67
POX04002	hypothetical protein	NA	NA	NA	26.89	48.32	0.85	Up	0.60
POX04007	Zinc finger C2H2-type	NA	NA	IPR007087:Zinc finger C2H2-type	62.48	35.15	-0.83	Down	0.63
POX04016	hypothetical protein	NA	NA	NA	1650.73	3042.48	0.88	Up	0.75
POX04017	hypothetical protein	NA	NA	NA	146.99	313.95	1.09	Up	0.78
POX04022	hypothetical protein	NA	NA	NA	84.42	139.25	0.72	Up	0.65
POX04035	hypothetical protein	NA	NA	NA	1533.09	2499.65	0.71	Up	0.69
POX04036	hypothetical protein	NA	NA	NA	3.55	16.90	2.25	Up	0.62
POX04048	hypothetical protein	NA	NA	NA	1058.24	2005.32	0.92	Up	0.76
POX04060	hypothetical protein	NA	NA	NA	156.20	94.96	-0.72	Down	0.65
POX04062	hypothetical protein	NA	NA	NA	1322.78	2350.66	0.83	Up	0.74
POX04064	hypothetical protein	NA	NA	NA	212.08	127.32	-0.74	Down	0.67
POX04075	hypothetical protein	NA	NA	NA	1491.86	2336.08	0.65	Up	0.67
POX04076	hypothetical protein	NA	NA	NA	1795.56	3191.09	0.83	Up	0.74
POX04101	hypothetical protein	NA	NA	NA	186.15	296.33	0.67	Up	0.65
POX04106	hypothetical protein	NA	NA	NA	359.76	536.34	0.58	Up	0.62
POX04109	hypothetical protein	NA	NA	NA	61.28	173.19	1.50	Up	0.83
POX04113	hypothetical protein	NA	NA	NA	27.70	62.06	1.16	Up	0.71
POX04118	hypothetical protein	NA	NA	NA	67.17	110.38	0.72	Up	0.63

POX04137	putative endo-beta-1,4-glucanase	Endo-beta-1,4-glucanase	CBM1;GH5	NA	335.09	150.79	-1.15	Down	0.80
POX04142	hypothetical protein	NA	NA	NA	53.63	26.85	-1.00	Down	0.66
POX04143	hypothetical protein	NA	NA	NA	261.73	144.88	-0.85	Down	0.72
POX04163	hypothetical protein	NA	NA	NA	10.53	25.72	1.29	Up	0.60
POX04179	hypothetical protein	NA	NA	NA	5.01	27.97	2.48	Up	0.73
POX04181	hypothetical protein	NA	NA	NA	998.91	1606.91	0.69	Up	0.68
POX04185	hypothetical protein	NA	NA	NA	18.80	40.24	1.10	Up	0.64
POX04200	hypothetical protein	NA	NA	NA	67.92	114.63	0.76	Up	0.65
POX04206	hypothetical protein	NA	NA	NA	80.68	147.38	0.87	Up	0.71
POX04207	hypothetical protein	NA	NA	NA	45.30	77.79	0.78	Up	0.63
POX04214	hypothetical protein	NA	NA	NA	118.94	416.71	1.81	Up	0.87
POX04258	hypothetical protein	NA	NA	NA	106.54	61.24	-0.80	Down	0.66
POX04260	hypothetical protein	NA	NA	NA	7.15	33.55	2.23	Up	0.75
POX04288	hypothetical protein	NA	NA	NA	108.15	202.85	0.91	Up	0.73
POX04310	hypothetical protein	NA	NA	NA	51.53	87.01	0.76	Up	0.63
POX04316	hypothetical protein	NA	NA	NA	10.60	42.86	2.02	Up	0.77
POX04321	hypothetical protein	NA	NA	NA	101.25	331.10	1.71	Up	0.86
POX04326	hypothetical protein	NA	NA	NA	92.64	156.47	0.76	Up	0.67
POX04328	hypothetical protein	NA	NA	NA	11.83	28.45	1.27	Up	0.62
POX04331	hypothetical protein	NA	NA	NA	58.60	99.43	0.76	Up	0.64
POX04333	hypothetical protein	NA	NA	NA	1161.14	2282.02	0.97	Up	0.78
POX04360	hypothetical protein	NA	NA	NA	187.45	353.27	0.91	Up	0.75
POX04373	hypothetical protein	NA	NA	NA	23.08	45.51	0.98	Up	0.63
POX04379	hypothetical protein	NA	NA	NA	176.74	286.52	0.70	Up	0.67
POX04384	hypothetical protein	NA	NA	NA	423.97	190.93	-1.15	Down	0.80
POX04392	hypothetical protein	NA	NA	NA	74.65	36.66	-1.03	Down	0.70
POX04405	hypothetical protein	NA	NA	NA	235.75	472.36	1.00	Up	0.77
POX04406	hypothetical protein	NA	NA	NA	113.80	39.93	-1.51	Down	0.81
POX04407	hypothetical protein	NA	NA	NA	228.48	99.96	-1.19	Down	0.80
POX04408	hypothetical protein	NA	NA	NA	1667.58	768.92	-1.12	Down	0.80
POX04411	hypothetical protein	NA	NA	NA	50.25	17.98	-1.48	Down	0.74
POX04448	hypothetical protein	NA	NA	NA	1273.38	1940.33	0.61	Up	0.64
POX04455	hypothetical protein	NA	NA	NA	246.54	435.51	0.82	Up	0.72
POX04458	hypothetical protein	NA	NA	NA	50.43	117.42	1.22	Up	0.77
POX04462	hypothetical protein	NA	NA	NA	2198.77	1157.90	-0.93	Down	0.77

POX04476	hypothetical protein	NA	NA	NA	30.33	67.94	1.16	Up	0.72
POX04480	hypothetical protein	NA	NA	NA	19.61	5.92	-1.73	Down	0.61
POX04486	hypothetical protein	NA	NA	NA	51.85	97.86	0.92	Up	0.70
POX04502	hypothetical protein	NA	NA	NA	10.24	26.01	1.34	Up	0.61
POX04510	hypothetical protein	NA	NA	IPR000679:Zinc finger GATA-type	62.91	36.50	-0.79	Down	0.61
POX04519	hypothetical protein	NA	NA	NA	15.50	35.89	1.21	Up	0.65
POX04524	hypothetical protein	NA	NA	NA	517.39	339.52	-0.61	Down	0.63
POX04546	hypothetical protein	NA	NA	NA	128.48	220.02	0.78	Up	0.69
POX04549	hypothetical protein	NA	NA	NA	166.57	66.82	-1.32	Down	0.80
POX04565	hypothetical protein	NA	NA	NA	145.54	245.61	0.75	Up	0.68
POX04593	hypothetical protein	NA	NA	NA	148.68	91.50	-0.70	Down	0.65
POX04594	hypothetical protein	NA	NA	NA	406.60	127.60	-1.67	Down	0.86
POX04608	hypothetical protein	NA	NA	NA	37.33	14.36	-1.38	Down	0.68
POX04612	hypothetical protein	NA	NA	NA	47.15	79.92	0.76	Up	0.63
POX04646	hypothetical protein	NA	NA	NA	85.09	36.04	-1.24	Down	0.76
POX04659	hypothetical protein	NA	NA	NA	16.14	48.96	1.60	Up	0.75
POX04673	sphingolipid long chain base-responsive protein LSP1-like	NA	NA	NA	57.12	96.60	0.76	Up	0.64
POX04686	hypothetical protein	NA	GH128	NA	228.94	392.75	0.78	Up	0.70
POX04735	hypothetical protein	NA	NA	NA	10.08	40.89	2.02	Up	0.76
POX04748	hypothetical protein	NA	NA	NA	97.90	187.81	0.94	Up	0.74
POX04751	hypothetical protein	NA	NA	NA	261.69	388.99	0.57	Up	0.61
POX04754	hypothetical protein	NA	NA	NA	22.58	44.52	0.98	Up	0.63
POX04764	hypothetical protein	NA	NA	NA	14.84	39.77	1.42	Up	0.70
POX04765	hypothetical protein	NA	NA	NA	66.26	133.53	1.01	Up	0.74
POX04768	hypothetical protein	NA	NA	NA	60.40	137.26	1.18	Up	0.78
POX04769	hypothetical protein	NA	NA	IPR007087:Zinc finger C2H2-type	108.88	60.34	-0.85	Down	0.68
POX04770	hypothetical protein	NA	NA	NA	91.60	54.90	-0.74	Down	0.63
POX04773	NA	NA	NA	NA	41.42	15.07	-1.46	Down	0.71
POX04781	hypothetical protein	NA	NA	NA	2028.17	1207.89	-0.75	Down	0.71

POX04803	hypothetical protein	NA	NA	NA	55.56	31.29	-0.83	Down	0.61
POX04814	hypothetical protein	NA	NA	NA	50.81	82.19	0.69	Up	0.60
POX04819	hypothetical protein	NA	NA	NA	806.66	1198.95	0.57	Up	0.62
POX04824	hypothetical protein	NA	NA	NA	36.93	16.84	-1.13	Down	0.64
POX04850	hypothetical protein	NA	NA	NA	988.00	1528.85	0.63	Up	0.65
POX04854	hypothetical protein	NA	NA	NA	19.82	48.73	1.30	Up	0.71
POX04860	hypothetical protein	NA	NA	IPR001356:Homeobox//IPR009057:Homeodomain-like	166.60	96.14	-0.79	Down	0.68
POX04863	hypothetical protein	NA	NA	NA	1086.11	2124.26	0.97	Up	0.78
POX04865	hypothetical protein	NA	NA	NA	32.28	86.92	1.43	Up	0.78
POX04866	hypothetical protein	NA	NA	NA	57.09	131.93	1.21	Up	0.78
POX04867	hypothetical protein	NA	NA	NA	54.59	96.83	0.83	Up	0.67
POX04868	hypothetical protein	NA	NA	NA	18.58	39.79	1.10	Up	0.64
POX04872	hypothetical protein	NA	NA	NA	339.43	181.14	-0.91	Down	0.74
POX04887	hypothetical protein	NA	NA	NA	46.97	84.25	0.84	Up	0.66
POX04895	putative alpha-mannosidase	NA	GH92	NA	58.63	30.79	-0.93	Down	0.65
POX04908	hypothetical protein	NA	NA	NA	89.00	55.24	-0.69	Down	0.61
POX04910	hypothetical protein	NA	AA2	NA	439.68	298.36	-0.56	Down	0.60
POX04923	hypothetical protein	NA	NA	NA	168.35	274.33	0.70	Up	0.67
POX04933	hypothetical protein	NA	NA	NA	50.24	27.56	-0.87	Down	0.61
POX04966	hypothetical protein	NA	NA	NA	103.01	195.33	0.92	Up	0.73
POX04984	hypothetical protein	NA	NA	NA	34.38	74.02	1.11	Up	0.72
POX04985	hypothetical protein	NA	NA	NA	119.43	186.46	0.64	Up	0.63
POX04986	hypothetical protein	NA	NA	NA	42.98	74.47	0.79	Up	0.63
POX04988	hypothetical protein	NA	NA	NA	99.89	152.65	0.61	Up	0.60
POX04991	hypothetical protein	NA	NA	NA	1300.71	2035.04	0.65	Up	0.67
POX04993	hypothetical protein	NA	NA	NA	66.37	104.52	0.66	Up	0.60
POX05007	hypothetical protein	NA	NA	NA	692.68	223.07	-1.63	Down	0.86
POX05041	hypothetical protein	NA	NA	IPR001138:Fungal transcriptional regulatory protein N-terminal	188.82	89.38	-1.08	Down	0.77
POX05045	hypothetical protein	NA	NA	NA	73.14	171.94	1.23	Up	0.79
POX05046	hypothetical protein	NA	NA	NA	58.65	109.42	0.90	Up	0.70

POX05049	hypothetical protein	NA	NA	NA	44.33	82.97	0.90	Up	0.68
POX05059	hypothetical protein	NA	NA	NA	102.06	165.53	0.70	Up	0.65
POX05068	hypothetical protein	NA	NA	NA	83.07	149.48	0.85	Up	0.70
POX05079	hypothetical protein	NA	NA	NA	28.73	78.10	1.44	Up	0.78
POX05081	hypothetical protein	NA	NA	NA	81.50	143.52	0.82	Up	0.69
POX05083	hypothetical protein	NA	NA	NA	244.31	394.01	0.69	Up	0.67
POX05087	hypothetical protein	NA	NA	NA	243.16	100.26	-1.28	Down	0.81
POX05094	hypothetical protein	NA	NA	NA	23.99	46.81	0.96	Up	0.63
POX05112	hypothetical protein	NA	NA	NA	155.75	280.02	0.85	Up	0.72
POX05119	hypothetical protein	NA	NA	NA	102.52	61.22	-0.74	Down	0.64
POX05122	hypothetical protein	NA	NA	NA	237.07	439.56	0.89	Up	0.74
POX05169	hypothetical protein	NA	NA	NA	66.45	35.40	-0.91	Down	0.66
POX05170	hypothetical protein	NA	NA	NA	47.50	88.83	0.90	Up	0.68
POX05177	hypothetical protein	NA	NA	NA	103.92	189.00	0.86	Up	0.71
POX05208	hypothetical protein	NA	NA	NA	59.90	102.29	0.77	Up	0.65
POX05218	hypothetical protein	NA	NA	NA	9.73	0.01	-9.93	Down	0.63
POX05233	hypothetical protein	NA	NA	NA	20.12	40.45	1.01	Up	0.62
POX05234	hypothetical protein	NA	NA	NA	56.05	98.49	0.81	Up	0.66
POX05288	hypothetical protein	NA	NA	NA	52.52	88.78	0.76	Up	0.64
POX05300	hypothetical protein	NA	NA	NA	27.07	11.69	-1.21	Down	0.60
POX05303	hypothetical protein	NA	NA	NA	108.36	189.72	0.81	Up	0.70
POX05304	hypothetical protein	NA	NA	NA	183.68	340.77	0.89	Up	0.74
POX05306	hypothetical protein	NA	NA	NA	23.73	7.95	-1.58	Down	0.63
POX05312	hypothetical protein	NA	NA	NA	103.03	161.54	0.65	Up	0.62
POX05322	hypothetical protein	NA	NA	NA	33.48	57.80	0.79	Up	0.60
POX05329	hypothetical protein	NA	NA	NA	20.03	4.68	-2.10	Down	0.64
POX05330	hypothetical protein	NA	NA	NA	122.13	43.23	-1.50	Down	0.81
POX05376	hypothetical protein	NA	NA	NA	38.40	70.67	0.88	Up	0.66
POX05378	hypothetical protein	NA	NA	NA	108.40	283.74	1.39	Up	0.82
POX05381	hypothetical protein	NA	NA	NA	24.87	48.00	0.95	Up	0.63
POX05404	hypothetical protein	NA	NA	NA	73.64	40.36	-0.87	Down	0.66
POX05405	hypothetical protein	NA	NA	NA	136.81	234.14	0.78	Up	0.69
POX05422	hypothetical protein	NA	NA	NA	965.69	1838.62	0.93	Up	0.77
POX05430	hypothetical protein	NA	NA	NA	53.51	29.13	-0.88	Down	0.63
POX05451	hypothetical protein	NA	GH125	NA	24.62	49.79	1.02	Up	0.65
POX05469	hypothetical protein	NA	NA	NA	53.84	88.14	0.71	Up	0.61
POX05477	NA	NA	NA	NA	220.90	116.97	-0.92	Down	0.74
POX05490	hypothetical protein	NA	NA	NA	10.21	32.77	1.68	Up	0.70
POX05492	hypothetical protein	NA	NA	NA	12.68	59.21	2.22	Up	0.81

POX05497	hypothetical protein	NA	NA	NA	998.37	2070.86	1.05	Up	0.79
POX05500	hypothetical protein	NA	CE10	NA	6.87	22.71	1.72	Up	0.64
POX05507	hypothetical protein	NA	NA	NA	254.06	507.58	1.00	Up	0.77
POX05511	hypothetical protein	NA	NA	NA	71.01	41.35	-0.78	Down	0.62
POX05512	hypothetical protein	NA	NA	NA	1539.81	891.58	-0.79	Down	0.72
POX05515	hypothetical protein	NA	NA	NA	41.81	189.20	2.18	Up	0.87
POX05521	hypothetical protein	NA	NA	NA	30.13	60.93	1.02	Up	0.68
POX05538	hypothetical protein	NA	NA	NA	79.37	180.84	1.19	Up	0.79
POX05540	hypothetical protein	beta-xylosidase/alpha-L-arabinofuranosidase	GH62	NA	31.36	13.32	-1.24	Down	0.63
POX05554	hypothetical protein	NA	NA	NA	9494.27	14904.94	0.65	Up	0.67
POX05558	hypothetical protein	NA	NA	NA	8.85	31.92	1.85	Up	0.71
POX05561	hypothetical protein	NA	NA	NA	132.72	282.54	1.09	Up	0.78
POX05565	hypothetical protein	NA	NA	NA	860.32	341.99	-1.33	Down	0.83
POX05567	hypothetical protein	NA	NA	NA	669.13	230.35	-1.54	Down	0.85
POX05569	hypothetical protein	NA	NA	NA	5.58	49.40	3.15	Up	0.82
POX05575	hypothetical protein	NA	NA	NA	4.79	31.61	2.72	Up	0.76
POX05577	hypothetical protein	NA	NA	NA	13.79	30.26	1.13	Up	0.60
POX05593	hypothetical protein	NA	NA	NA	298.41	157.29	-0.92	Down	0.74
POX05619	hypothetical protein	NA	NA	NA	139.77	88.56	-0.66	Down	0.62
POX05646	hypothetical protein	NA	NA	NA	8.62	36.27	2.07	Up	0.75
POX05650	hypothetical protein	NA	NA	NA	385.40	681.16	0.82	Up	0.73
POX05651	hypothetical protein	NA	NA	NA	25.08	58.64	1.23	Up	0.72
POX05660	hypothetical protein	NA	NA	NA	488.74	719.53	0.56	Up	0.61
POX05669	hypothetical protein	NA	NA	NA	32.51	62.98	0.95	Up	0.67
POX05679	hypothetical protein	NA	NA	NA	91.15	191.28	1.07	Up	0.77
POX05686	hypothetical protein	NA	NA	NA	450.90	191.15	-1.24	Down	0.81
POX05690	hypothetical protein	NA	NA	NA	45.50	74.87	0.72	Up	0.60
POX05697	hypothetical protein	NA	NA	NA	19.33	38.71	1.00	Up	0.61
POX05725	hypothetical protein	NA	NA	NA	42.30	72.69	0.78	Up	0.62
POX05726	hypothetical protein	NA	NA	IPR007087:Zinc finger C2H2-type	70.45	24.86	-1.50	Down	0.78
POX05742	hypothetical protein	NA	NA	NA	6.68	24.45	1.87	Up	0.67
POX05744	hypothetical protein	NA	NA	NA	9.45	24.53	1.38	Up	0.61

POX05747	hypothetical protein	NA	NA	NA	40.80	84.15	1.04	Up	0.71
POX05770	hypothetical protein	NA	NA	NA	23.57	58.84	1.32	Up	0.73
POX05783	hypothetical protein	NA	NA	NA	55.66	89.56	0.69	Up	0.61
POX05818	hypothetical protein	NA	NA	NA	2.73	18.25	2.74	Up	0.66
POX05844	Nucleic acid-binding OB-fold	NA	NA	NA	295.96	705.82	1.25	Up	0.82
POX05873	hypothetical protein	NA	NA	NA	64.98	117.38	0.85	Up	0.69
POX05880	Ribosomal protein L1 superfamily	NA	NA	NA	831.66	1363.85	0.71	Up	0.69
POX05896	hypothetical protein	NA	NA	NA	23.34	50.10	1.10	Up	0.67
POX05921	hypothetical protein	NA	NA	NA	39.26	82.38	1.07	Up	0.72
POX05929	hypothetical protein	NA	NA	NA	29.42	10.39	-1.50	Down	0.66
POX05932	hypothetical protein	NA	NA	NA	53.75	101.49	0.92	Up	0.70
POX05933	hypothetical protein	NA	NA	NA	268.39	421.52	0.65	Up	0.65
POX05936	hypothetical protein	NA	NA	NA	257.90	386.07	0.58	Up	0.61
POX05939	putative endo-beta-1,3-glucanase	NA	GH55	NA	73.76	17.91	-2.04	Down	0.82
POX05948	hypothetical protein	NA	NA	NA	28.49	52.32	0.88	Up	0.62
POX05956	hypothetical protein	NA	NA	NA	509.86	955.17	0.91	Up	0.76
POX05968	hypothetical protein	Cellulose	AA9	NA	37.64	80.85	1.10	Up	0.72
POX05974	hypothetical protein	NA	NA	NA	80.44	39.20	-1.04	Down	0.71
POX05981	hypothetical protein	NA	NA	NA	23.93	46.93	0.97	Up	0.63
POX05984	hypothetical protein	NA	NA	NA	68.21	149.20	1.13	Up	0.77
POX05987	hypothetical protein	NA	NA	NA	47.51	13.12	-1.86	Down	0.77
POX05995	hypothetical protein	NA	NA	NA	108.00	216.57	1.00	Up	0.76
POX05997	hypothetical protein	NA	NA	NA	22.57	47.58	1.08	Up	0.66
POX06002	hypothetical protein	NA	NA	NA	350.91	222.67	-0.66	Down	0.65
POX06004	hypothetical protein	NA	NA	NA	39.46	67.34	0.77	Up	0.61
POX06006	hypothetical protein	NA	NA	NA	6.12	26.11	2.09	Up	0.70
POX06008	hypothetical protein	NA	NA	NA	9.00	99.09	3.46	Up	0.87
POX06017	hypothetical protein	NA	NA	NA	43.56	96.10	1.14	Up	0.75
POX06028	hypothetical protein	NA	NA	NA	1713.12	2740.42	0.68	Up	0.68
POX06035	hypothetical protein	NA	NA	NA	60.55	99.19	0.71	Up	0.62
POX06038	hypothetical protein	NA	NA	NA	217.54	140.60	-0.63	Down	0.63
POX06043	hypothetical protein	NA	NA	NA	86.46	48.78	-0.83	Down	0.66
POX06064	hypothetical protein	NA	NA	NA	1.18	14.53	3.62	Up	0.63
POX06075	hypothetical protein	NA	NA	NA	25.50	47.65	0.90	Up	0.62
POX06086	hypothetical protein	NA	NA	NA	1535.79	925.58	-0.73	Down	0.70

POX06098	hypothetical protein	NA	NA	NA	47.82	81.32	0.77	Up	0.63
POX06099	hypothetical protein	NA	NA	NA	601.88	878.45	0.55	Up	0.61
POX06117	hypothetical protein	NA	NA	NA	415.59	660.52	0.67	Up	0.67
POX06146	hypothetical protein	NA	NA	NA	281.96	510.24	0.86	Up	0.74
POX06156	hypothetical protein	NA	NA	NA	74.04	116.01	0.65	Up	0.61
POX06162	hypothetical protein	NA	NA	NA	1185.48	2135.79	0.85	Up	0.74
POX06168	hypothetical protein	NA	NA	NA	127.38	37.74	-1.76	Down	0.84
POX06190	hypothetical protein	NA	NA	NA	121.89	193.85	0.67	Up	0.64
POX06217	hypothetical protein	NA	NA	NA	38.29	78.78	1.04	Up	0.71
POX06236	hypothetical protein	NA	NA	NA	356.38	610.80	0.78	Up	0.71
POX06242	putative alpha-glucosidase	NA	GH31	NA	15.40	47.52	1.63	Up	0.75
POX06247	hypothetical protein	NA	NA	NA	12.45	31.20	1.33	Up	0.65
POX06262	hypothetical protein	NA	NA	NA	1125.40	1683.01	0.58	Up	0.63
POX06278	hypothetical protein	NA	NA	NA	56.15	32.28	-0.80	Down	0.60
POX06293	hypothetical protein	NA	NA	NA	46.91	83.64	0.83	Up	0.65
POX06306	hypothetical protein	NA	NA	NA	178.41	97.44	-0.87	Down	0.71
POX06307	hypothetical protein	NA	NA	NA	3094.15	694.26	-2.16	Down	0.89
POX06308	hypothetical protein	NA	NA	NA	5754.86	2208.19	-1.38	Down	0.84
POX06342	hypothetical protein	NA	NA	NA	49.04	101.33	1.05	Up	0.73
POX06345	hypothetical protein	NA	NA	NA	51.29	131.18	1.35	Up	0.80
POX06356	hypothetical protein	NA	NA	NA	28.72	60.89	1.08	Up	0.69
POX06368	hypothetical protein	NA	NA	NA	61.29	103.24	0.75	Up	0.64
POX06379	hypothetical protein	NA	NA	NA	49.80	95.63	0.94	Up	0.70
POX06395	hypothetical protein	NA	NA	NA	883.78	255.17	-1.79	Down	0.87
POX06398	hypothetical protein	NA	NA	NA	173.01	270.75	0.65	Up	0.64
POX06399	hypothetical protein	NA	NA	NA	140.44	228.11	0.70	Up	0.66
POX06413	hypothetical protein	NA	NA	NA	96.44	39.79	-1.28	Down	0.77
POX06417	hypothetical protein	NA	NA	NA	132.71	217.22	0.71	Up	0.66
POX06418	hypothetical protein	NA	NA	NA	19.23	3.34	-2.53	Down	0.66

POX06425	hypothetical protein	NA	NA	IPR001138:Fungal transcriptional regulatory protein N-terminal//IPR007219:Fungal specific transcription factor	71.52	37.98	-0.91	Down	0.67
POX06431	hypothetical protein	NA	NA	NA	547.39	889.65	0.70	Up	0.68
POX06442	hypothetical protein	NA	NA	NA	18.03	36.70	1.02	Up	0.61
POX06482	hypothetical protein	NA	NA	NA	235.55	148.65	-0.66	Down	0.64
POX06500	putative glycogen/starch phosphorylase	NA	GT35	NA	1088.86	616.71	-0.82	Down	0.73
POX06509	hypothetical protein	NA	NA	IPR004827:Basic-leucine zipper (bZIP) transcription factor	109.98	69.80	-0.66	Down	0.61
POX06513	hypothetical protein	NA	NA	NA	5284.68	2377.39	-1.15	Down	0.81
POX06516	hypothetical protein	NA	NA	NA	1829.23	3809.68	1.06	Up	0.79
POX06521	hypothetical protein	NA	NA	NA	27.98	11.47	-1.29	Down	0.62
POX06528	hypothetical protein	NA	NA	NA	39.19	100.36	1.36	Up	0.78
POX06530	hypothetical protein	NA	NA	NA	491.06	295.19	-0.73	Down	0.69
POX06543	hypothetical protein	NA	NA	NA	140.62	64.00	-1.14	Down	0.77
POX06549	hypothetical protein	NA	NA	NA	20.40	43.41	1.09	Up	0.65
POX06577	putative chitooligosaccharide deacetylase	NA	CE4	NA	21.80	43.78	1.01	Up	0.63
POX06580	hypothetical protein	NA	NA	NA	312.34	553.31	0.82	Up	0.72
POX06602	hypothetical protein	NA	AA7	NA	13.45	32.74	1.28	Up	0.65
POX06603	hypothetical protein	NA	NA	NA	4.61	27.96	2.60	Up	0.73
POX06628	hypothetical protein	NA	NA	NA	82.21	139.54	0.76	Up	0.67
POX06642	hypothetical protein	NA	NA	NA	210.62	117.37	-0.84	Down	0.71

POX06656	hypothetical protein	NA	NA	NA	141.28	67.17	-1.07	Down	0.75
POX06658	hypothetical protein	NA	NA	NA	26.79	54.37	1.02	Up	0.67
POX06689	putative endo-beta-1,4-galactanase	Endo-beta-1,4-galactanase	GH53	NA	2.43	17.72	2.87	Up	0.65
POX06696	hypothetical protein	NA	NA	NA	1423.48	2254.35	0.66	Up	0.67
POX06705	hypothetical protein	NA	NA	NA	1027.34	1713.74	0.74	Up	0.70
POX06707	hypothetical protein	NA	NA	NA	122.12	74.58	-0.71	Down	0.64
POX06714	hypothetical protein	NA	NA	NA	153.33	394.74	1.36	Up	0.83
POX06726	hypothetical protein	NA	NA	NA	53.14	90.44	0.77	Up	0.64
POX06734	hypothetical protein	NA	NA	NA	8.92	30.14	1.76	Up	0.70
POX06740	hypothetical protein	NA	NA	NA	7.51	21.51	1.52	Up	0.60
POX06746	hypothetical protein	NA	NA	NA	31.42	57.13	0.86	Up	0.63
POX06767	Vacuolar protein sorting-associated protein 74	NA	NA	NA	232.20	349.92	0.59	Up	0.62
POX06783	putative endo-beta-1,4-xylanase	Endo-beta-1,4-xylanase	CBM1;GH11	NA	136.89	49.77	-1.46	Down	0.81
POX06785	hypothetical protein	NA	NA	NA	88.91	50.11	-0.83	Down	0.66
POX06788	hypothetical protein	NA	NA	NA	1340.60	2500.22	0.90	Up	0.76
POX06794	hypothetical protein	NA	NA	NA	1629.29	2964.80	0.86	Up	0.75
POX06798	hypothetical protein	NA	NA	NA	104.19	63.84	-0.71	Down	0.63
POX06803	hypothetical protein	NA	NA	NA	128.71	63.30	-1.02	Down	0.74
POX06804	hypothetical protein	NA	NA	NA	219.50	121.96	-0.85	Down	0.72
POX06807	hypothetical protein	NA	NA	NA	1834.25	3629.02	0.98	Up	0.78
POX06831	hypothetical protein	NA	NA	NA	18.97	41.88	1.14	Up	0.66
POX06853	hypothetical protein	NA	NA	NA	1161.53	1739.23	0.58	Up	0.63
POX06859	putative glycogenin	NA	GT8	NA	150.25	89.91	-0.74	Down	0.66
POX06875	hypothetical protein	NA	NA	NA	2071.19	3960.69	0.94	Up	0.77

POX06892	hypothetical protein	NA	NA	IPR011991:Winged helix repressor DNA-binding	973.04	1675.95	0.78	Up	0.72
POX06904	hypothetical protein	NA	NA	NA	1073.51	689.94	-0.64	Down	0.66
POX06922	hypothetical protein	NA	NA	NA	35.84	61.45	0.78	Up	0.61
POX06939	hypothetical protein	NA	NA	NA	154.76	272.31	0.82	Up	0.71
POX06944	hypothetical protein	NA	NA	NA	86.92	44.74	-0.96	Down	0.70
POX06946	hypothetical protein	NA	NA	NA	163.38	289.30	0.82	Up	0.71
POX06947	hypothetical protein	NA	NA	NA	1701.35	3325.02	0.97	Up	0.78
POX06951	hypothetical protein	NA	NA	NA	62.77	100.71	0.68	Up	0.61
POX06961	hypothetical protein	NA	NA	NA	1632.32	3301.04	1.02	Up	0.79
POX06963	hypothetical protein	NA	NA	NA	26.52	77.59	1.55	Up	0.79
POX06964	hypothetical protein	NA	NA	NA	72.61	193.66	1.42	Up	0.82
POX06970	hypothetical protein	NA	NA	NA	82.84	34.05	-1.28	Down	0.76
POX06978	hypothetical protein	NA	NA	NA	28.94	57.46	0.99	Up	0.67
POX06994	hypothetical protein	NA	NA	NA	87.18	139.02	0.67	Up	0.63
POX07002	hypothetical protein	NA	NA	NA	1202.38	1866.00	0.63	Up	0.66
POX07011	hypothetical protein	NA	NA	NA	1503.37	2454.92	0.71	Up	0.69
POX07016	hypothetical protein	NA	NA	NA	141.84	234.70	0.73	Up	0.67
POX07027	hypothetical protein	NA	NA	NA	35.02	62.61	0.84	Up	0.63
POX07029	hypothetical protein	NA	NA	NA	64.07	101.88	0.67	Up	0.61
POX07035	hypothetical protein	NA	NA	NA	72.67	42.47	-0.78	Down	0.62
POX07040	hypothetical protein	NA	NA	NA	97.66	50.66	-0.95	Down	0.71
POX07059	hypothetical protein	NA	NA	NA	75.44	147.46	0.97	Up	0.73
POX07078	hypothetical protein	NA	NA	IPR009057:Homeo domain-like	50.71	24.38	-1.06	Down	0.67
POX07097	hypothetical protein	NA	NA	NA	962.75	1595.67	0.73	Up	0.70
POX07098	putative UDP-GlcNAc: peptide beta-N-acetylglucosaminyltransfer ase	NA	GT41	NA	101.73	53.28	-0.93	Down	0.70
POX07107	hypothetical protein	NA	NA	NA	380.58	728.47	0.94	Up	0.76
POX07116	hypothetical protein	NA	NA	NA	80.64	48.62	-0.73	Down	0.61

POX07118	hypothetical protein	NA	NA	NA	65.33	104.21	0.67	Up	0.61
POX07123	hypothetical protein	NA	NA	NA	63.14	22.36	-1.50	Down	0.77
POX07141	hypothetical protein	NA	NA	NA	22.52	47.15	1.07	Up	0.66
POX07143	hypothetical protein	NA	AA6	NA	863.88	562.08	-0.62	Down	0.64
POX07145	putative chitinase	NA	GH18	NA	35.41	97.48	1.46	Up	0.79
POX07147	hypothetical protein	NA	NA	NA	40.73	105.57	1.37	Up	0.79
POX07150	hypothetical protein	NA	NA	NA	34.79	62.48	0.84	Up	0.63
POX07153	hypothetical protein	NA	NA	NA	137.74	264.28	0.94	Up	0.75
POX07173	hypothetical protein	NA	NA	NA	161.29	36.19	-2.16	Down	0.86
POX07226	hypothetical protein	NA	NA	NA	51.31	20.61	-1.32	Down	0.72
POX07227	hypothetical protein	NA	NA	NA	478.64	298.75	-0.68	Down	0.67
POX07234	hypothetical protein	NA	NA	NA	221.17	468.12	1.08	Up	0.79
POX07264	hypothetical protein	NA	GT41	NA	152.76	92.96	-0.72	Down	0.65
POX07289	hypothetical protein	NA	NA	NA	15.49	32.63	1.07	Up	0.60
POX07291	hypothetical protein	NA	NA	NA	199.20	328.80	0.72	Up	0.68
POX07300	hypothetical protein	NA	NA	NA	139.89	257.60	0.88	Up	0.73
POX07311	hypothetical protein	NA	NA	NA	11.41	27.46	1.27	Up	0.61
POX07378	putative cutinase	NA	CE5	NA	1.54	20.15	3.71	Up	0.70
POX07380	putative lysozyme	NA	GH25	NA	130.46	1124.90	3.11	Up	0.91
POX07386	hypothetical protein	NA	NA	NA	21.69	43.93	1.02	Up	0.64
POX07393	NA	NA	NA	NA	986.89	565.42	-0.80	Down	0.72
POX07401	hypothetical protein	NA	NA	NA	35.32	88.68	1.33	Up	0.77
POX07420	hypothetical protein	NA	NA	NA	154.65	89.67	-0.79	Down	0.68
POX07428	hypothetical protein	NA	NA	NA	19.16	63.89	1.74	Up	0.79
POX07431	hypothetical protein	NA	NA	NA	36.54	78.61	1.11	Up	0.72
POX07448	hypothetical protein	NA	NA	NA	71.02	39.02	-0.86	Down	0.65
POX07454	hypothetical protein	NA	NA	NA	59.43	32.66	-0.86	Down	0.63
POX07461	hypothetical protein	NA	NA	NA	46.31	89.14	0.94	Up	0.70
POX07471	hypothetical protein	NA	NA	NA	34.45	15.65	-1.14	Down	0.63
POX07476	hypothetical protein	NA	NA	NA	12.61	28.90	1.20	Up	0.61
POX07481	hypothetical protein	NA	NA	NA	51.39	92.26	0.84	Up	0.67
POX07497	hypothetical protein	NA	NA	NA	107.40	37.64	-1.51	Down	0.81
POX07500	hypothetical protein	NA	NA	NA	29.90	53.14	0.83	Up	0.61
POX07501	hypothetical protein	NA	NA	NA	102.18	203.82	1.00	Up	0.75
POX07505	hypothetical protein	NA	NA	NA	47.70	79.58	0.74	Up	0.62
POX07513	hypothetical protein	NA	NA	NA	1485.39	2207.11	0.57	Up	0.63
POX07516	hypothetical protein	NA	NA	NA	2708.01	1356.66	-1.00	Down	0.78
POX07517	hypothetical protein	NA	NA	NA	122.53	65.57	-0.90	Down	0.71

POX07522	hypothetical protein	NA	NA	IPR011991:Winged helix repressor DNA-binding	38.52	68.39	0.83	Up	0.64
POX07525	hypothetical protein	NA	NA	NA	258.80	536.09	1.05	Up	0.78
POX07526	hypothetical protein	NA	NA	NA	5.70	24.46	2.10	Up	0.68
POX07531	hypothetical protein	NA	NA	NA	349.08	170.61	-1.03	Down	0.77
POX07532	hypothetical protein	NA	NA	NA	1243.40	851.69	-0.55	Down	0.61
POX07534	putative beta-1,3-glucanosyltransferase	NA	GH17	NA	3631.61	2126.64	-0.77	Down	0.72
POX07539	hypothetical protein	NA	NA	NA	61.12	131.90	1.11	Up	0.76
POX07573	putative beta-N-acetylhexosaminidase	NA	GH3	NA	14.04	37.07	1.40	Up	0.68
POX07576	hypothetical protein	NA	NA	NA	604.05	1359.43	1.17	Up	0.81
POX07601	hypothetical protein	NA	NA	NA	57.29	184.13	1.68	Up	0.84
POX07608	hypothetical protein	NA	NA	NA	206.03	386.44	0.91	Up	0.75
POX07619	hypothetical protein	NA	NA	NA	33.95	71.47	1.07	Up	0.71
POX07668	hypothetical protein	NA	NA	NA	50.69	27.40	-0.89	Down	0.62
POX07669	hypothetical protein	NA	NA	NA	32.86	12.74	-1.37	Down	0.66
POX07678	hypothetical protein	NA	NA	NA	18.62	38.60	1.05	Up	0.63
POX07681	hypothetical protein	NA	NA	NA	121.32	77.62	-0.64	Down	0.60
POX07707	hypothetical protein	NA	NA	NA	19.83	39.48	0.99	Up	0.62
POX07713	hypothetical protein	NA	NA	NA	53.63	90.91	0.76	Up	0.64
POX07804	hypothetical protein	NA	NA	NA	34.74	68.08	0.97	Up	0.68
POX07814	hypothetical protein	NA	NA	NA	14.23	38.69	1.44	Up	0.70
POX07822	hypothetical protein	NA	NA	NA	742.14	1104.78	0.57	Up	0.62
POX07847	hypothetical protein	NA	NA	NA	54.19	29.02	-0.90	Down	0.63
POX07849	hypothetical protein	NA	NA	NA	22.68	50.50	1.15	Up	0.69
POX07862	hypothetical protein	NA	NA	NA	343.73	20.39	-4.08	Down	0.90
POX07863	hypothetical protein	NA	NA	NA	433.11	79.99	-2.44	Down	0.89
POX07864	hypothetical protein	NA	NA	NA	45.40	4.59	-3.31	Down	0.81
POX07865	hypothetical protein	NA	NA	NA	69.84	5.54	-3.66	Down	0.85

POX07868	hypothetical protein	NA	NA	NA	14.57	0.71	-4.36	Down	0.64
POX07888	hypothetical protein	NA	NA	NA	48.64	24.06	-1.02	Down	0.65
POX07894	hypothetical protein	NA	NA	NA	178.58	116.49	-0.62	Down	0.61
POX07899	hypothetical protein	NA	NA	NA	21.53	44.80	1.06	Up	0.65
POX07903	hypothetical protein	NA	NA	NA	7.01	20.74	1.57	Up	0.60
POX07928	hypothetical protein	NA	NA	NA	36.72	17.89	-1.04	Down	0.62
POX07938	hypothetical protein	NA	NA	IPR001138:Fungal transcriptional regulatory protein N-terminal	170.45	106.72	-0.68	Down	0.64
POX07971	putative chitinase	NA	GH18	NA	797.76	466.77	-0.77	Down	0.71
POX07980	hypothetical protein	NA	NA	NA	1284.60	2199.17	0.78	Up	0.72
POX07996	hypothetical protein	NA	NA	NA	86.45	178.78	1.05	Up	0.76
POX08016	hypothetical protein	NA	NA	NA	38.72	70.79	0.87	Up	0.65
POX08017	putative alpha-1,6-mannanase	NA	GH76	NA	8.86	29.78	1.75	Up	0.69
POX08021	hypothetical protein	NA	NA	NA	74.51	117.06	0.65	Up	0.61
POX08022	hypothetical protein	NA	NA	NA	81.43	183.84	1.17	Up	0.79
POX08030	hypothetical protein	NA	NA	NA	184.21	329.85	0.84	Up	0.72
POX08032	hypothetical protein	NA	NA	NA	30.71	83.35	1.44	Up	0.78
POX08033	hypothetical protein	NA	NA	NA	12.71	28.73	1.18	Up	0.61
POX08034	putative endo-alpha-1,4-polygalactosaminidase	NA	GH114	NA	141.65	283.97	1.00	Up	0.76
POX08048	hypothetical protein	NA	NA	NA	8.85	24.96	1.50	Up	0.63
POX08053	hypothetical protein	NA	NA	NA	67.43	113.49	0.75	Up	0.65
POX08056	hypothetical protein	NA	NA	NA	54.01	87.01	0.69	Up	0.60
POX08066	hypothetical protein	NA	NA	NA	4432.25	2850.52	-0.64	Down	0.66
POX08085	hypothetical protein	NA	NA	NA	1468.05	3686.68	1.33	Up	0.84
POX08089	hypothetical protein	NA	NA	NA	14.28	1.48	-3.27	Down	0.62
POX08101	hypothetical protein	NA	NA	NA	22.31	41.88	0.91	Up	0.60
POX08103	hypothetical protein	NA	NA	NA	38.97	3.12	-3.64	Down	0.80
POX08111	hypothetical protein	NA	NA	NA	96.34	153.83	0.68	Up	0.64

POX08125	hypothetical protein	NA	NA	NA	67.48	117.07	0.79	Up	0.67
POX08145	hypothetical protein	NA	NA	NA	115.35	247.74	1.10	Up	0.78
POX08146	Molybdopterin biosynthesis MoaE	NA	NA	NA	26.11	48.52	0.89	Up	0.62
POX08179	hypothetical protein	NA	NA	NA	55.32	15.84	-1.80	Down	0.78
POX08182	hypothetical protein	NA	NA	NA	40.15	11.15	-1.85	Down	0.75
POX08193	hypothetical protein	NA	NA	NA	41.12	21.15	-0.96	Down	0.61
POX08195	hypothetical protein	NA	NA	NA	98.83	38.68	-1.35	Down	0.78
POX08198	hypothetical protein	NA	NA	NA	1435.44	2513.21	0.81	Up	0.73
POX08199	hypothetical protein	NA	NA	NA	1409.88	2675.77	0.92	Up	0.76
POX08200	hypothetical protein	NA	NA	NA	416.40	260.85	-0.67	Down	0.66
POX08205	hypothetical protein	NA	NA	NA	128.19	199.32	0.64	Up	0.63
POX08210	hypothetical protein	NA	NA	NA	1304.14	2467.44	0.92	Up	0.76
POX08217	hypothetical protein	NA	NA	NA	444.19	798.22	0.85	Up	0.74
POX08223	hypothetical protein	NA	NA	NA	36.81	63.98	0.80	Up	0.62
POX08225	hypothetical protein	NA	NA	NA	972.25	1638.24	0.75	Up	0.71
POX08240	hypothetical protein	NA	NA	NA	67.04	141.41	1.08	Up	0.76
POX08243	hypothetical protein	NA	NA	NA	65.61	141.68	1.11	Up	0.76
POX08249	hypothetical protein	NA	NA	NA	78.28	128.48	0.71	Up	0.64
POX08298	hypothetical protein	NA	NA	NA	81.89	154.37	0.91	Up	0.72
POX08307	hypothetical protein	NA	NA	NA	235.81	1160.42	2.30	Up	0.89
POX08310	hypothetical protein	NA	NA	NA	38.05	18.99	-1.00	Down	0.61
POX08314	hypothetical protein	NA	NA	NA	662.42	1269.18	0.94	Up	0.77
POX08316	hypothetical protein	NA	NA	NA	85.14	50.70	-0.75	Down	0.63
POX08324	hypothetical protein	NA	NA	NA	28.85	55.51	0.94	Up	0.65
POX08335	hypothetical protein	NA	NA	NA	46.90	78.21	0.74	Up	0.62
POX08339	hypothetical protein	NA	NA	NA	62.74	30.69	-1.03	Down	0.68
POX08359	hypothetical protein	NA	NA	NA	4043.62	2256.99	-0.84	Down	0.74
POX08366	hypothetical protein	NA	NA	NA	24.31	7.54	-1.69	Down	0.65
POX08371	hypothetical protein	NA	NA	NA	23.34	66.05	1.50	Up	0.77
POX08372	hypothetical protein	NA	NA	NA	50.73	24.31	-1.06	Down	0.67
POX08437	hypothetical protein	NA	NA	NA	35.37	70.93	1.00	Up	0.69
POX08450	hypothetical protein	NA	NA	NA	25.35	56.88	1.17	Up	0.71
POX08451	hypothetical protein	NA	NA	NA	5.82	24.45	2.07	Up	0.68
POX08454	hypothetical protein	NA	NA	NA	503.37	1209.54	1.26	Up	0.83
POX08456	hypothetical protein	NA	NA	NA	56.01	109.93	0.97	Up	0.72
POX08482	hypothetical protein	NA	NA	NA	188.08	82.78	-1.18	Down	0.79

POX08486	hypothetical protein	NA	NA	NA	160.06	411.18	1.36	Up	0.83
POX08489	hypothetical protein	NA	NA	NA	63.54	101.28	0.67	Up	0.61
POX08497	hypothetical protein	NA	NA	NA	67.75	128.78	0.93	Up	0.72
POX08502	hypothetical protein	NA	NA	NA	2447.63	4349.24	0.83	Up	0.74
POX08525	hypothetical protein	NA	NA	NA	1451.85	2314.22	0.67	Up	0.68
POX08528	hypothetical protein	NA	NA	NA	218.71	354.56	0.70	Up	0.67
POX08541	hypothetical protein	NA	NA	NA	31.30	65.25	1.06	Up	0.70
POX08562	hypothetical protein	NA	NA	NA	3377.53	2265.39	-0.58	Down	0.63
POX08583	hypothetical protein	NA	NA	NA	321.69	1033.83	1.68	Up	0.87
POX08602	hypothetical protein	NA	NA	NA	41.05	16.81	-1.29	Down	0.68
POX08633	hypothetical protein	NA	NA	NA	50.55	21.76	-1.22	Down	0.70
POX08645	hypothetical protein	NA	NA	NA	7.98	24.03	1.59	Up	0.63
POX08646	hypothetical protein	NA	NA	NA	95.35	213.33	1.16	Up	0.79
POX08672	v-SNARE protein	NA	NA	NA	237.56	401.80	0.76	Up	0.70
POX08712	hypothetical protein	NA	NA	NA	22.03	51.11	1.21	Up	0.70
POX08716	hypothetical protein	NA	NA	NA	384.37	169.83	-1.18	Down	0.80
POX08718	hypothetical protein	NA	NA	NA	344.96	181.73	-0.92	Down	0.75
POX08721	hypothetical protein	NA	NA	NA	56.70	109.59	0.95	Up	0.71
POX08724	hypothetical protein	NA	NA	NA	161.78	79.15	-1.03	Down	0.75
POX08731	hypothetical protein	NA	NA	NA	56.10	90.83	0.70	Up	0.61
POX08744	hypothetical protein	NA	NA	NA	23.83	6.03	-1.98	Down	0.67
POX08745	hypothetical protein	NA	NA	NA	27.89	9.96	-1.49	Down	0.65
POX08746	hypothetical protein	NA	NA	NA	4.99	19.42	1.96	Up	0.63
POX08750	hypothetical protein	NA	NA	NA	29.33	89.68	1.61	Up	0.81
POX08760	putative alpha,alpha-trehalose-phosphate synthase [UDP-forming]	NA	GT20;GT4	NA	17.74	2.93	-2.60	Down	0.64
POX08764	hypothetical protein	NA	NA	NA	94.78	161.31	0.77	Up	0.68
POX08772	hypothetical protein	NA	NA	NA	67.25	109.46	0.70	Up	0.63
POX08776	hypothetical protein	NA	NA	NA	57.27	105.47	0.88	Up	0.69
POX08780	hypothetical protein	NA	NA	NA	56.77	31.38	-0.86	Down	0.63
POX08783	hypothetical protein	NA	NA	NA	1.24	24.75	4.32	Up	0.74
POX08789	hypothetical protein	NA	NA	NA	51.19	124.60	1.28	Up	0.79
POX08803	hypothetical protein	NA	NA	NA	35.91	95.52	1.41	Up	0.79
POX08804	hypothetical protein	NA	NA	NA	197.49	319.33	0.69	Up	0.67

POX08811	hypothetical protein	NA	NA	NA	23.74	47.26	0.99	Up	0.64
POX08822	hypothetical protein	NA	NA	NA	614.97	1838.31	1.58	Up	0.86
POX08831	hypothetical protein	NA	NA	NA	411.25	192.93	-1.09	Down	0.79
POX08840	hypothetical protein	NA	NA	NA	26.63	58.54	1.14	Up	0.70
POX08848	hypothetical protein	NA	NA	NA	19.33	39.36	1.03	Up	0.62
POX08858	hypothetical protein	NA	NA	NA	15.87	39.96	1.33	Up	0.69
POX08864	hypothetical protein	NA	NA	NA	24.60	50.18	1.03	Up	0.66
POX08877	hypothetical protein	NA	NA	NA	14.16	86.42	2.61	Up	0.85
POX08887	hypothetical protein	NA	NA	NA	17.87	47.41	1.41	Up	0.72
POX08906	hypothetical protein	NA	NA	NA	153.90	275.03	0.84	Up	0.72
POX08908	NA	NA	NA	NA	12.22	0.01	-10.26	Down	0.69
POX08920	hypothetical protein	NA	NA	NA	58.79	148.85	1.34	Up	0.80
POX08946	hypothetical protein	NA	NA	NA	41.95	113.65	1.44	Up	0.80
POX08954	carbohydrate binding domain-containing protein	NA	NA	NA	198.57	79.28	-1.32	Down	0.81
POX08956	hypothetical protein	NA	NA	NA	362.45	182.81	-0.99	Down	0.76
POX08958	hypothetical protein	NA	NA	NA	47.14	87.07	0.89	Up	0.68
POX08961	hypothetical protein	NA	NA	NA	32.55	67.73	1.06	Up	0.70
POX08990	putative endo-beta-1,4-xylanase	Endo-beta-1,4-xylanase	GH10	NA	58.36	20.29	-1.52	Down	0.76
POX09003	hypothetical protein	NA	NA	NA	98.54	50.72	-0.96	Down	0.71
POX09017	hypothetical protein	NA	NA	NA	162.36	269.94	0.73	Up	0.68
POX09023	hypothetical protein	NA	NA	NA	103.20	159.06	0.62	Up	0.61
POX09025	hypothetical protein	NA	NA	NA	32.94	8.71	-1.92	Down	0.72
POX09028	hypothetical protein	NA	NA	NA	257.76	529.71	1.04	Up	0.78
POX09040	hypothetical protein	NA	NA	NA	677.05	1094.90	0.69	Up	0.68
POX09062	hypothetical protein	NA	NA	NA	227.48	346.77	0.61	Up	0.63
POX09071	hypothetical protein	NA	NA	NA	36.82	65.96	0.84	Up	0.64
POX09088	hypothetical protein	NA	NA	IPR001138:Fungal transcriptional regulatory protein N-terminal	8.48	35.33	2.06	Up	0.74

POX09095	hypothetical protein	NA	NA	NA	67.81	126.06	0.89	Up	0.71
POX09160	hypothetical protein	NA	NA	NA	44.79	91.68	1.03	Up	0.72
POX09163	hypothetical protein	NA	NA	NA	40.09	12.43	-1.69	Down	0.73
POX09192	hypothetical protein	NA	NA	NA	1308.32	2134.27	0.71	Up	0.69
POX09193	hypothetical protein	NA	NA	NA	52.27	86.88	0.73	Up	0.62
POX09194	hypothetical protein	NA	NA	NA	105.11	61.38	-0.78	Down	0.65
POX09198	hypothetical protein	NA	NA	NA	40.98	70.08	0.77	Up	0.62
POX09204	hypothetical protein	NA	NA	NA	75.19	148.75	0.98	Up	0.74
POX09210	hypothetical protein	NA	NA	NA	25.99	50.41	0.96	Up	0.64
POX09243	Ribosomal protein S23	NA	NA	NA	2410.07	5555.51	1.20	Up	0.82
POX09271	hypothetical protein	NA	NA	NA	20.16	39.71	0.98	Up	0.61
POX09280	Ribosomal protein L6	NA	NA	NA	523.11	886.80	0.76	Up	0.71
POX09284	hypothetical protein	NA	NA	NA	54.24	266.88	2.30	Up	0.88
POX09285	hypothetical protein	NA	NA	NA	241.74	397.71	0.72	Up	0.68
POX09287	hypothetical protein	NA	NA	NA	273.62	158.06	-0.79	Down	0.70
POX09332	hypothetical protein	NA	NA	NA	457.58	86.61	-2.40	Down	0.89
POX09333	hypothetical protein	NA	NA	NA	161.11	81.16	-0.99	Down	0.74
POX09334	hypothetical protein	NA	NA	NA	213.29	70.42	-1.60	Down	0.84
POX09335	hypothetical protein	NA	NA	NA	428.95	73.58	-2.54	Down	0.89
POX09336	hypothetical protein	NA	NA	NA	565.15	245.90	-1.20	Down	0.81
POX09337	hypothetical protein	NA	NA	NA	1003.03	317.88	-1.66	Down	0.86
POX09338	hypothetical protein	NA	NA	NA	299.44	127.96	-1.23	Down	0.81
POX09339	hypothetical protein	NA	NA	NA	79.51	26.32	-1.59	Down	0.80
POX09340	hypothetical protein	NA	NA	NA	388.52	50.48	-2.94	Down	0.90
POX09345	hypothetical protein	NA	NA	NA	21.58	116.76	2.44	Up	0.86
POX09352	alpha-amylase Amy13A	NA	CBM20;GH13	NA	1.10	23.87	4.44	Up	0.74
POX09358	hypothetical protein	NA	NA	NA	534.39	289.82	-0.88	Down	0.74
POX09378	hypothetical protein	NA	NA	NA	58.25	32.74	-0.83	Down	0.62
POX09379	hypothetical protein	NA	NA	NA	40.22	14.19	-1.50	Down	0.71
POX09402	hypothetical protein	NA	NA	NA	84.63	46.02	-0.88	Down	0.67
POX09430	hypothetical protein	NA	NA	NA	63.86	109.31	0.78	Up	0.66
POX09432	hypothetical protein	NA	NA	NA	84.83	137.37	0.70	Up	0.64
POX09458	hypothetical protein	NA	NA	NA	46.70	20.15	-1.21	Down	0.69
POX09495	hypothetical protein	NA	NA	NA	181.07	284.41	0.65	Up	0.64
POX09521	hypothetical protein	NA	NA	NA	217.53	113.01	-0.94	Down	0.74
POX09526	hypothetical protein	NA	NA	NA	88.61	138.79	0.65	Up	0.62
POX09533	hypothetical protein	NA	NA	NA	19.29	43.60	1.18	Up	0.67
POX09537	hypothetical protein	NA	NA	NA	989.78	1442.01	0.54	Up	0.61

POX09538	hypothetical protein	NA	NA	NA	83.59	147.25	0.82	Up	0.69
POX09552	NA	NA	NA	NA	42.76	16.32	-1.39	Down	0.70
POX09559	NA	NA	NA	NA	66.10	107.39	0.70	Up	0.63
POX09637	hypothetical protein	NA	NA	NA	1388.42	2532.80	0.87	Up	0.75
POX09658	hypothetical protein	NA	NA	NA	163.18	269.51	0.72	Up	0.67
POX09661	hypothetical protein	NA	NA	NA	94.82	59.46	-0.67	Down	0.60
POX09674	hypothetical protein	NA	NA	NA	87.36	29.75	-1.55	Down	0.80
POX09675	hypothetical protein	NA	NA	NA	57.95	23.42	-1.31	Down	0.73
POX09676	hypothetical protein	NA	NA	NA	601.93	313.80	-0.94	Down	0.76
POX09677	hypothetical protein	NA	NA	NA	991.93	533.10	-0.90	Down	0.76
POX09717	NA	NA	NA	NA	37.63	71.43	0.92	Up	0.67
POX09752	hypothetical protein	NA	NA	IPR001138:Fungal transcriptional regulatory protein N-terminal	238.15	104.70	-1.19	Down	0.80
POX09759	hypothetical protein	NA	NA	NA	368.73	237.73	-0.63	Down	0.64
POX09760	hypothetical protein	NA	NA	NA	97.07	57.71	-0.75	Down	0.64
POX09767	hypothetical protein	NA	NA	NA	32.15	77.23	1.26	Up	0.75
POX09768	hypothetical protein	NA	NA	NA	56.24	105.38	0.91	Up	0.70
POX09786	hypothetical protein	NA	NA	NA	36.10	83.34	1.21	Up	0.75
POX09822	hypothetical protein	NA	NA	NA	160.08	400.87	1.32	Up	0.82
POX09823	hypothetical protein	NA	NA	NA	24.67	51.36	1.06	Up	0.67
POX09827	hypothetical protein	NA	NA	NA	7.24	63.68	3.14	Up	0.84
