# Supplementary material for: Identification of an essential regulator controlling the production of raw-starch-digesting glucoamylase in Penicillium oxalicum
Source: Biotechnol Biofuels. 2019 Jan 4;12:7. doi: 10.1186/s13068-018-1345-z (PMC6318894; doi:10.1186/s13068-018-1345-z)
Supplement: Supplementary file 4 — Additional file 4: Table S3. List of 23 candidate regulatory genes determined in this study that regulate raw starch-degrading enzymes production in P. oxalicum HP7-1. [file 13068_2018_1345_MOESM4_ESM.pdf]

Additional file 4: Table S3. List of 23 candidate regulatory genes determined in this study that regulate raw starch-degrading enzymes production in *P. oxalicum* HP7-1

Name	InterPro annotation of predicted protein	Domain description of predicted protein
POX00852	IPR001594	Zinc finger, DHHC-type
POX00981	IPR007087	Zinc finger, C2H2-type
POX01907	IPR001005	Myb/SANT DNA binding domain
POX02290	IPR005930<br>IPR009057<br>IPR011764	Pyruvate carboxylase;<br>Homeodomain-like;<br>Biotin carboxylation domain
POX02452	IPR007087	Zinc finger, C2H2-type
POX02944	IPR001138	Zinc finger, Zn2Cys6 type
POX03446	IPR001138<br>IPR007219	Zinc finger, Zn2Cys6 type;<br>Fungal_Trans
POX03789	IPR003163	APSES-type DNA-binding domain
POX03827	IPR009044	ssDNA-binding transcriptional regulator
POX04007	IPR007087	Zinc finger, C2H2-type
POX04510	IPR000679	Zinc finger, GATA-type
POX04769	IPR007087	Zinc finger, C2H2-type
POX04860	IPR009057	Homeodomain-like
POX05041	IPR001138	Zinc finger, Zn2Cys6 type
POX05726	IPR007087	Zinc finger, C2H2-type
POX06425	IPR001138<br>IPR007219	Zinc finger, Zn2Cys6 type;<br>Fungal_Trans
POX06509	IPR004827	Basic-leucine zipper (bZIP) domain
POX06892	IPR011991	Winged helix repressor DNA-binding domain

POX07078	IPR000949	ELM2 domain;
	IPR001025	Bromo adjacent homology (BAH) domain;
	IPR001965	Zinc finger, PHD-type;
	IPR009057	Homeodomain-like
POX07522	IPR011991	Winged helix repressor DNA-binding domain
POX07938	IPR001138	Zinc finger, Zn2Cys6 type
POX09088	IPR001138	Zinc finger, Zn2Cys6 type
POX09752	IPR001138	Zn2Cys6 fungal-type DNA-binding domain
	IPR001451	Hexapeptide repeat
	IPR024688	Maltose/galactoside acetyltransferase
