# Supplementary material for: Identification of an essential regulator controlling the production of raw-starch-digesting glucoamylase in Penicillium oxalicum
Source: Biotechnol Biofuels. 2019 Jan 4;12:7. doi: 10.1186/s13068-018-1345-z (PMC6318894; doi:10.1186/s13068-018-1345-z)
Supplement: Supplementary file 6 — Additional file 6: Table S4. Primers used in this study. [file 13068_2018_1345_MOESM6_ESM.pdf]

Additional file 6: Table S4. Primers used in this study.

Primer name	Sequence（5'-3'）
**Primers used for the construction of deletion mutants**	
POX00852-L-F	CCGCAGCATAGGATGAAGGA
POX00852-L-R	GGTAATCCTTCTTTCTAGAAGTGCCCTGCGGGGGAA
POX00852-R-F	CAATATCATCTTCTGTCGACCATGAACAATGTGTGTGTGTCAGAA
POX00852-R-R	ACCCTCGTAAACTCGCTCCA
POX00852-N-F	CCTGTCAATCCCCAGTCCT
POX00852-N-R	TCCAAACTGTGAGCGTCG
POX00852-V-F	TCTCGTCGTTCTAGGGATTT
POX00852-V-R	TGGCATACCCAGAGCATCA
POX01907-L-F	CGAGCCGCCCAGTAAAGG
POX01907-L-R	GGTAATCCTTCTTTCTAGACCGGGCCACCCGATGA
POX01907-R-F	CAATATCATCTTCTGTCGACGTTGGACGTGCGACATGAG
POX01907-R-R	GCGTCTGCGTCATTCACA
POX01907-N-F	CCAGCAAGTTTCCGATTC
POX01907-N-R	GGACGCCTCATAACCTCTAT
POX01907-V-F	GTTCCTGCCTTCGGTTCA
POX01907-V-R	TCGCATCACTCGGGTCAA
POX03446-L-F	GGAGCGACCGATTGACCT
POX03446-L-R	GGTAATCCTTCTTTCTAGAGGGGCAGATGGATCTCAC
POX03446-R-F	CAATATCATCTTCTGTCGACGGACCTACAGGCCAATGTGT
POX03446-R-R	TCTAAGCGGCGGACTACC
POX03446-V-F	GAGGCCCTGGTCCAGTTG
POX03446-V-R	CGAATCTCGCTTGGGTTG
POX03446-N-F	CCTTTCGTCGGGTACGTT
POX03446-N-R	CCTCATACTGTATCAGGGTTG
POX03789-L-F	TGCTCGTTCGGATTTGCC
POX03789-L-R	TTTAGAGGTAATCCTTCTTTCTAGACGAAGTTGGGCTGGGG
POX03789-R-F	TCCTTCAATATCATCTTCTGTCGACGAATCATTTCTCGCTTGGAAAG
POX03789-R-R	CTCCAACCTCTTCGCCTCC
POX03789-N-F	TTTTTACCTTTCTGGTTGCTGC
POX03789-N-R	CCCACTGTCCTGTCCACTTTT
POX03789-V-F	CTTCACCACCAGGCAATG
POX03789-V-R	AGCGGTCGTTCTTCAAATC
POX05041-L-F	ATGTGGGCTCACCGTGTAAC
POX05041-L-R	TTTAGAGGTAATCCTTCTTTCTAGAGACATGAGCGGAAAACCCT
POX05041-R-F	TCCTTCAATATCATCTTCTGTCGACACTGATGATACAAAGTCACCTCCC
POX05041-R-R	CATCGTGGCACACAACTGGT
POX05041-N-F	CACCGAAAGTCTGGAGCGT
POX05041-N-R	ACACGATTGAAGTTGGCATTAG
POX05041-V-F	TGGGCACAGTCCGTCCTA

POX05041-V-R	GGTCGGTTGTTTGTCTTGG
POX06509-L-F	ACGGCGAACGGTAGGATA
POX06509-L-R	GGTAATCCTTCTTTCTAGAGATGTTTGTGATAGATGGTGCC
POX06509-R-F	CAATATCATCTTCTGTCGACTTCCCCCTTTAGCGTCTT
POX06509-R-R	AGTTCCACTCGGGACGGT
POX06509-N-F	GAACGGACGCCAGGGA
POX06509-N-R	TGAACAGTTTCTCCCTTCG
POX06509-V-F	GGAACATCCCATGCTCAA
POX06509-V-R	CTGGCAGAGTGTTCTCAAAC
POX07078-L-F	AAAGGGCGTGTAGGTTCG
POX07078-L-R	GGTAATCCTTCTTTCTAGATGGGTACCAGCCTTAGTCATC
POX07078-R-F	CAATATCATCTTCTGTCGACTTGGTTGTCCATTCCCCAA
POX07078-R-R	GAACATGGCATCCTGGTTTT
POX07078-N-F	TTCAAAGCCGTCCGCAATC
POX07078-N-R	TTCCCTCCAAACGCACC
POX07078-V-F	TCCAAGTCCGACAGTGAGAA
POX07078-V-R	AGTGGATGGTGGTGAATGAGA
POX07522-L-F	AGAATGGTGGACAAGACGAGG
POX07522-L-R	GGTAATCCTTCTTTCTAGAtCTGAGTCCGATTCTTTCTCTCAAG
POX07522-R-F	CAATATCATCTTCTGTCGACTAGGGGGGTTATTACTTGATGAA
POX07522-R-R	CCAGTAGCGACCAAAAGATGAA
POX07522-N-F	ATGCGGTGTAGTCATCATCAGA
POX07522-N-R	TGTCGTTATCGTCTTTGGAGTCT
POX07522-V-F	TCGGTCCTGAACGAAGAT
POX07522-V-R	TCAACACCCACAGCCAAA
POX07938-L-F	CGACCGATTTTATCCAGGCT
POX07938-L-R	TTTAGAGGTAATCCTTCTTTCTAGACTTGTGTGGGATGTGAGGG
POX07938-R-F	TCCTTCAATATCATCTTCTGTCGACGTCCCGGTGGGTGATGAA
POX07938-R-R	GAAGTGGGCTGAGCGTGTA
POX07938-N-F	GCACCGCACTGAAACCGT
POX07938-N-R	ATACAAAACTACCAAACACGAACG
POX07938-V-F	CAAGCGTGGCTCGTTCAT
POX07938-V-R	GTCGTCGTAATCCGTGGG
POX09088-L-F	GTCTGGGGATGAATCGGTG
POX09088-L-R	TTTAGAGGTAATCCTTCTTTCTAGACGGGTTTGAAGGATACAGACAC
POX09088-R-F	TCCTTCAATATCATCTTCTGTCGACCATGAACTTGAGGGACCTCTGAG
POX09088-R-R	CACCTTGATACACCGTTGGAG
POX09088-N-F	GAGAGGCAGGTGTCAGTCGTC
POX09088-N-R	AATCTGCGGGGCTTCCTG
POX09088-V-F	CGTCGTCTGGAAGCAGTTGT
POX09088-V-R	AATCCATTTGCCGTTCGTAG
POX09752-L-F	CAACGCTATGGATCGTGAAT
POX09752-L-R	GGTAATCCTTCTTTCTAGACACTTTTTGGTCGGGAGGTAC

POX09752-R-F	CAATATCATCTTCTGTCGACAATACCCCTTTCGTTTGTTTCG
POX09752-R-R	TGGCGACTGAGATGCTCTTTCC
POX09752-N-F	TGTACCATAGGGTGCCGTAG
POX09752-N-R	GAGACTCTGCTCGGGACAAC
POX09752-V-F	TACGGCTGTCAATGGTAGGGA
POX09752-V-R	ATTCAAGTGAACGCCAGGATAA
G418-F	TCTAGAAAGAAGGATTACC
G418-R	GTCGACAGAAGATGATATT
G418-V-F	GTGAATGCTCCGTAACACCCAAT
G418-V-R	CGCTACTGCTTACAAGTGGGCTGAT
**Primers used for the complementation**	
CPOX01907-L-F	TTCGCACGAATTGCTCCG
CPOX01907-L-R	GCCGAACGGAGATATGCTAA
POX05007-L-F	GCCAAGCTTGGTACCGAGCTCGGATCCAGTTGCTGGCTGCTGGAGT
POX05007-L-R	TGAAGCTATGGTGTGTGGGGATGGACGAGACGGTACGAT
Ben-F	CCCACACACCATAGCTTCA
Ben-R	TTCCAGCACACTGGCGGCCGTTAAGCTTGCAAATTAAAGCCTTC
CPOX05007-R-F	CTTCAATATCATCTTCTGTCGACATGTAGATCACCAAGTTGCA
CPOX05007-R-R	GATCAGCGTCTGAACCTGGGC
CPOX01907-N-F	TCCAACGGAGTCCATTTCAAC
CPOX01907-N-R	CTGAAAGTCGTATCCAGCACA
Ben-V-F	CAAAACACCCAAGCACAGC
Ben-V-R	AATTAAAGCCTTCGAGCGTC
CPOX01907-V-F	TTCGCACGAATTGCTCCG
CPOX01907-V-R	GCCGAACGGAGAATATGCTAA
**Primers used for the probe amplification in Southern hybridization**	
POX01907-probe-F	CGACCTTGGCTTGAACCG
POX01907-probe-R	TGGACTACCACCACCCTGAT
**Primers used for RT-qPCR analysis**	
RT-actin-F	CTCCATCCAGGCCGTTCTG
RT-actin-R	CATGAGGTAGTCGGTCAAGTCAC
RT-POX01356-F	CCTCGGTGAGCCCAAGTT
RT-POX01356-R	CCAAAGTCAATCAAGGCAA
RT-POX02412-F	TATGTGGATTCCTTCCGCTCTA
RT-POX02412-R	ATGGATTGCCTCCTTGGT
RT-POX09352-F	CTGACGGCTGCCCAATG
RT-POX09352-R	CCAAATCGCAGTAAATCCC
