# Supplementary material for: Identification of an essential regulator controlling the production of raw-starch-digesting glucoamylase in Penicillium oxalicum
Source: Biotechnol Biofuels. 2019 Jan 4;12:7. doi: 10.1186/s13068-018-1345-z (PMC6318894; doi:10.1186/s13068-018-1345-z)
Supplement: Supplementary file 8 — Additional file 8: Table S5. List of 1003 genes differentially expressed in ∆POX01907 as compared with the parental strain ∆PoxKu70 grown on soluble corn starch as the sole carbon source. [file 13068_2018_1345_MOESM8_ESM.pdf]

Additional file 8: Table S5. List of 1003 genes differentially expressed in the mutant Δ*POX01907* as compared with the parental strain Δ*PoxKu70* grown on soluble starch as the sole carbon source

Gene ID	Production	CWDEs	CAZy family	Putative Transcription factor	Δ*PoxKu70*_FPKM	Δ*POX01907*_FPKM	log2 (Δ*POX01907*_FPKM/Δ*PoxKu70*_FPKM)	Padj	Up/Down
POX08949	hypothetical protein	NA	NA	NA	8382.89	16780.93	1.00	7.87E-08	Up
POX01907	hypothetical protein	NA	NA	IPR001005:Myb, DNA-binding//IPR009057:H	452.78	3.03	-7.22	8.61E-53	Down
POX07890	putative pectate lyase	Pectate	PL1	NA	28.97	58.01	1.00	0.005474	Up
POX07855	hypothetical protein	NA	NA	NA	357.70	716.79	1.00	1.21E-05	Up
POX02691	hypothetical protein	NA	NA	NA	77.56	155.48	1.00	0.000441	Up
POX00445	hypothetical protein	NA	NA	NA	37.67	75.54	1.00	0.006499	Up
POX03994	hypothetical protein	NA	NA	NA	164.65	330.31	1.00	7.05E-05	Up
POX04258	hypothetical protein	NA	NA	NA	496.48	996.21	1.00	1.08E-05	Up
POX03535	hypothetical protein	NA	CE12	NA	112.20	225.15	1.00	1.86E-05	Up
POX01695	hypothetical protein	NA	NA	NA	14872.86	112.09	-7.05	1.7E-241	Down
POX03272	hypothetical protein	NA	NA	NA	17.74	35.63	1.01	0.023658	Up
POX09358	hypothetical protein	NA	NA	NA	196258.71	2925.09	-6.07	2.41E-95	Down
POX01694	hypothetical protein	NA	NA	NA	4078.90	90.90	-5.49	4.42E-92	Down
POX00861	hypothetical protein	NA	NA	NA	1808.56	46.08	-5.29	1.9E-107	Down
POX03627	hypothetical protein	NA	NA	NA	43.37	87.14	1.01	0.002206	Up
POX02478	hypothetical protein	NA	NA	NA	36.55	73.48	1.01	0.004119	Up
POX00862	hypothetical protein	NA	NA	NA	3148.04	91.78	-5.10	7.83E-82	Down
POX01287	hypothetical protein	NA	NA	NA	368.75	741.32	1.01	0.000105	Up
POX00965	putative cellulose synthase	NA	GT2	NA	1393.06	2805.25	1.01	1.24E-07	Up
POX03801	hypothetical protein	NA	NA	NA	18.57	37.40	1.01	0.027036	Up
POX09333	hypothetical protein	NA	NA	NA	142.03	286.30	1.01	0.000366	Up
POX07874	hypothetical protein	NA	NA	NA	202.53	408.49	1.01	0.000596	Up
POX09499	hypothetical protein	NA	GH32	NA	18.86	38.04	1.01	0.041467	Up
POX03325	hypothetical protein	NA	NA	NA	1521.39	3070.21	1.01	1.8E-06	Up
POX02944	hypothetical protein	NA	NA	IPR001138:Fungal transcriptional regulatory protein, N-terminal	579.50	1170.25	1.01	3.01E-07	Up
POX00022	hypothetical protein	NA	NA	NA	2635.90	5331.15	1.02	2.73E-07	Up

POX03228	putative alpha-1,3-glucan synthase	NA	GH13;GT4;GT5	NA	1536.60	3108.14	1.02	0.000159	Up
POX07535	putative endo-beta-1,4-glucanase	Endo-beta-1,4-glucanase	GH12	NA	18.98	38.47	1.02	0.022521	Up
POX04380	hypothetical protein	NA	NA	NA	225.08	7.06	-4.99	7.92E-43	Down
POX01692	hypothetical protein	NA	NA	NA	3814.38	120.20	-4.99	9.9E-121	Down
POX04067	hypothetical protein	NA	NA	NA	53.59	108.75	1.02	0.001905	Up
POX08876	hypothetical protein	NA	CE9	NA	73.85	149.96	1.02	0.000274	Up
POX03666	hypothetical protein	NA	NA	NA	635.50	22.56	-4.82	4.47E-22	Down
POX01693	hypothetical protein	NA	NA	NA	2611.65	103.21	-4.66	1.17E-94	Down
POX07503	hypothetical protein	NA	NA	NA	238.93	485.22	1.02	0.000126	Up
POX04289	hypothetical protein	NA	NA	NA	985.84	2005.34	1.02	8.69E-06	Up
POX09088	hypothetical protein	NA	NA	IPR001138:Fungal transcriptional regulatory protein, N-terminal	75.76	154.14	1.02	0.000307	Up
POX06104	hypothetical protein	NA	NA	NA	61.07	124.43	1.03	0.00071	Up
POX09025	hypothetical protein	NA	NA	NA	454.89	20.28	-4.49	7.19E-65	Down
POX00396	hypothetical protein	NA	NA	IPR007219:Fungal specific transcription factor	312.87	637.53	1.03	4.03E-06	Up
POX01883	hypothetical protein	NA	NA	NA	323.08	659.06	1.03	7.5E-07	Up
POX06117	hypothetical protein	NA	NA	NA	2957.80	6039.19	1.03	8.91E-08	Up
POX02886	hypothetical protein	NA	NA	NA	128.56	262.58	1.03	0.000351	Up
POX09210	hypothetical protein	NA	NA	NA	466.43	21.04	-4.47	8.16E-59	Down
POX04536	hypothetical protein	NA	CBM24;GH71	NA	568.49	1162.53	1.03	5.64E-07	Up
POX08483	putative alpha-N-acetylglucosaminidase	NA	GH89	NA	140.59	7.83	-4.17	4.19E-27	Down
POX02834	hypothetical protein	NA	NA	NA	447.79	26.91	-4.06	9.32E-54	Down
POX00870	hypothetical protein	NA	NA	NA	1856.90	112.90	-4.04	1E-108	Down
POX02810	hypothetical protein	NA	NA	NA	17.30	35.37	1.03	0.023528	Up
POX01320	hypothetical protein	NA	NA	NA	233.55	14.37	-4.02	1.06E-37	Down
POX09798	hypothetical protein	NA	NA	NA	90.25	5.69	-3.99	1.32E-21	Down
POX08898	hypothetical protein	NA	NA	NA	37.69	2.63	-3.84	4.89E-12	Down
POX02405	hypothetical protein	NA	NA	NA	280.42	573.60	1.03	4.52E-05	Up
POX08221	hypothetical protein	NA	NA	NA	280.05	573.40	1.03	1.94E-08	Up
POX06145	hypothetical protein	NA	NA	NA	10101.51	712.29	-3.83	3.93E-64	Down
POX08245	hypothetical protein	NA	CE10;CE1	NA	99.02	7.45	-3.73	4.37E-21	Down
POX09365	hypothetical protein	NA	NA	NA	62545.05	5056.98	-3.63	3.72E-44	Down
POX03912	hypothetical protein	NA	NA	NA	25.39	2.11	-3.59	1.21E-09	Down

POX08194	hypothetical protein	NA	NA	NA	123.34	10.30	-3.58	3.16E-23	Down
POX00863	hypothetical protein	NA	NA	NA	30.03	2.51	-3.58	2.76E-10	Down
POX09819	hypothetical protein	NA	NA	NA	149.54	12.50	-3.58	2.2E-27	Down
POX01608	hypothetical protein	NA	NA	NA	250.20	513.26	1.04	9.89E-06	Up
POX05155	hypothetical protein	NA	NA	NA	71.92	6.32	-3.51	8.13E-17	Down
POX09361	hypothetical protein	NA	NA	NA	27746.81	2549.82	-3.44	9.88E-13	Down
POX08228	NIPSNAP family protein	NA	NA	NA	852.77	1751.80	1.04	6.01E-10	Up
POX06195	hypothetical protein	NA	NA	IPR001356:Homeobox//IPR007087:Zinc finger, C2H2-type//IPR009057:Homeodomain-like	328.56	675.45	1.04	4.54E-08	Up
POX08289	hypothetical protein	NA	NA	NA	1948.53	4006.62	1.04	1.8E-05	Up
POX04400	hypothetical protein	NA	NA	NA	29.85	61.40	1.04	0.00573	Up
POX02113	hypothetical protein	NA	NA	NA	483.05	993.49	1.04	1.4E-06	Up
POX04197	hypothetical protein	NA	NA	NA	29.39	60.49	1.04	0.00433	Up
POX05960	hypothetical protein	NA	NA	NA	2605.16	5364.57	1.04	1.04E-06	Up
POX06149	hypothetical protein	NA	NA	NA	524.98	1081.29	1.04	2.83E-10	Up
POX05584	hypothetical protein	NA	NA	IPR001138:Fungal transcriptional regulatory protein, N-terminal	58.01	119.51	1.04	0.00014	Up
POX09195	hypothetical protein	NA	NA	NA	138.33	12.75	-3.44	1.31E-25	Down
POX03448	hypothetical protein	NA	NA	NA	70.57	6.59	-3.42	2.35E-16	Down
POX08195	hypothetical protein	NA	NA	NA	1053.27	101.82	-3.37	1.03E-73	Down
POX09345	hypothetical protein	NA	NA	NA	67.72	6.66	-3.35	8.37E-15	Down
POX07173	hypothetical protein	NA	NA	NA	465.78	46.77	-3.32	1.68E-46	Down
POX02011	hypothetical protein	NA	NA	NA	378.37	38.27	-3.31	8.59E-35	Down
POX08341	hypothetical protein	NA	NA	NA	926.75	94.97	-3.29	7.05E-65	Down
POX05776	hypothetical protein	NA	NA	NA	39.86	82.13	1.04	0.001406	Up
POX07963	putative beta-glucosidase	beta-glucosidase	GH3	NA	212.26	438.26	1.05	2.19E-06	Up
POX06818	Heat shock protein DnaJ, N-terminal	NA	GT41	NA	497.52	1029.52	1.05	7.57E-08	Up
POX02221	hypothetical protein	NA	NA	NA	159.70	330.64	1.05	3.03E-06	Up
POX08497	hypothetical protein	NA	NA	NA	562.55	60.59	-3.21	1.96E-47	Down
POX02436	hypothetical protein	NA	NA	NA	70.96	147.12	1.05	4E-05	Up
POX03838	hypothetical protein	NA	NA	NA	456.81	947.65	1.05	1.41E-05	Up

POX09252	hypothetical protein	NA	NA	IPR001138:Fungal transcriptional regulatory protein, N-terminal	20.17	41.94	1.06	0.012241	Up
POX07067	hypothetical protein	NA	NA	NA	28.90	60.19	1.06	0.004255	Up
POX03565	hypothetical protein	NA	NA	NA	18.86	39.38	1.06	0.014559	Up
POX03742	hypothetical protein	NA	NA	NA	376.24	786.45	1.06	8.53E-05	Up
POX00936	hypothetical protein	NA	NA	IPR001138:Fungal transcriptional regulatory protein, N-terminal	133.11	279.18	1.07	2.52E-06	Up
POX04344	hypothetical protein	NA	NA	NA	637.19	1338.39	1.07	4.55E-05	Up
POX06146	hypothetical protein	NA	NA	NA	11186.93	1226.38	-3.19	9.78E-33	Down
POX06231	hypothetical protein	NA	NA	NA	707.56	77.97	-3.18	1.89E-11	Down
POX03873	hypothetical protein	NA	NA	IPR001138:Fungal transcriptional regulatory protein, N-terminal	453.02	51.60	-3.13	5.73E-41	Down
POX07909	hypothetical protein	NA	NA	NA	58.10	6.63	-3.13	1.39E-12	Down
POX08001	hypothetical protein	NA	NA	NA	662.23	1394.49	1.07	3.7E-05	Up
POX04093	hypothetical protein	NA	NA	NA	3775.82	7978.69	1.08	1.27E-11	Up
POX00642	hypothetical protein	NA	NA	NA	341.26	723.49	1.08	8.77E-06	Up
POX05285	hypothetical protein	NA	NA	NA	472.77	1004.17	1.09	7.93E-08	Up
POX06798	hypothetical protein	NA	NA	NA	1119.38	2378.35	1.09	5.48E-06	Up
POX05482	hypothetical protein	NA	NA	NA	4940.93	10508.42	1.09	1.7E-08	Up
POX00977	hypothetical protein	NA	NA	NA	408.76	871.25	1.09	7.84E-09	Up
POX07186	hypothetical protein	NA	NA	NA	603.59	1287.83	1.09	3.86E-06	Up
POX09687	hypothetical protein	NA	NA	NA	83.40	9.60	-3.12	1.02E-16	Down
POX06554	hypothetical protein	NA	NA	NA	195.63	417.49	1.09	0.000122	Up
POX03699	hypothetical protein	NA	NA	NA	14.57	31.10	1.09	0.026537	Up
POX05733	hypothetical protein	NA	NA	NA	59.00	6.88	-3.10	5E-13	Down
POX02233	hypothetical protein	NA	NA	NA	58.14	6.83	-3.09	4.79E-13	Down
POX00338	hypothetical protein	NA	NA	NA	28.69	3.39	-3.08	1.22E-07	Down
POX07081	hypothetical protein	NA	NA	NA	84.64	10.02	-3.08	7.85E-16	Down
POX07269	hypothetical protein	NA	NA	NA	13.72	1.63	-3.07	1.92E-06	Down
POX02267	hypothetical protein	NA	NA	NA	3411.04	7291.68	1.10	4.13E-06	Up

POX06073	hypothetical protein	NA	NA	NA	228.81	489.84	1.10	1.32E-05	Up
POX04893	hypothetical protein	NA	AA3;AA8	NA	287.75	618.47	1.10	5.74E-06	Up
POX07272	hypothetical protein	NA	NA	NA	660.25	1419.69	1.10	6.42E-08	Up
POX07211	hypothetical protein	NA	NA	NA	423.90	911.53	1.10	2.41E-09	Up
POX02074	hypothetical protein	NA	NA	NA	4354.94	9390.94	1.11	6.26E-10	Up
POX01155	hypothetical protein	NA	NA	NA	15.93	34.35	1.11	0.028164	Up
POX00900	hypothetical protein	NA	NA	NA	22.42	48.44	1.11	0.004661	Up
POX03005	putative cutinase	NA	CE5	NA	5456.87	11799.20	1.11	3.07E-06	Up
POX04132	hypothetical protein	NA	NA	NA	340.96	738.67	1.12	9.89E-07	Up
POX01989	hypothetical protein	NA	NA	NA	36.22	78.51	1.12	0.003157	Up
POX04879	hypothetical protein	NA	NA	NA	118.43	14.09	-3.07	4.28E-20	Down
POX07666	hypothetical protein	NA	NA	NA	28.37	3.39	-3.07	2.03E-08	Down
POX09359	hypothetical protein	NA	NA	NA	748.71	90.90	-3.04	1.93E-27	Down
POX09440	hypothetical protein	NA	NA	NA	5134.91	624.53	-3.04	1.86E-85	Down
POX00372	hypothetical protein	NA	NA	NA	52.50	114.24	1.12	0.00016	Up
POX02068	hypothetical protein	NA	NA	NA	1413.35	174.00	-3.02	1.67E-52	Down
POX07423	hypothetical protein	NA	CBM18	NA	3659.02	453.43	-3.01	1.93E-75	Down
POX08861	putative acetyl xylan esterase	Acetyl	CBM1;CE2	NA	23.40	50.99	1.12	0.003369	Up
POX05421	hypothetical protein	NA	NA	NA	10.58	23.09	1.13	0.034658	Up
POX01927	hypothetical protein	NA	NA	NA	111.21	13.81	-3.01	9.47E-17	Down
POX02480	hypothetical protein	NA	NA	NA	788.89	1724.91	1.13	5.33E-09	Up
POX06650	hypothetical protein	NA	NA	NA	224.93	492.51	1.13	2.28E-07	Up
POX00701	hypothetical protein	NA	NA	NA	15.66	1.95	-3.01	1.76E-06	Down
POX07270	hypothetical protein	NA	NA	NA	24.30	3.04	-3.00	1.46E-07	Down
POX00979	hypothetical protein	NA	NA	NA	381.20	835.05	1.13	5.17E-05	Up
POX02069	hypothetical protein	NA	NA	NA	1537.64	200.27	-2.94	1.92E-55	Down
POX08890	hypothetical protein	NA	NA	NA	31.34	4.12	-2.93	4.13E-08	Down
POX01088	hypothetical protein	NA	NA	NA	5447.03	11943.20	1.13	1.12E-10	Up
POX08836	hypothetical protein	NA	NA	NA	11.40	25.01	1.13	0.045045	Up
POX07092	hypothetical protein	NA	NA	NA	239.51	525.67	1.13	1.22E-09	Up
POX04355	hypothetical protein	NA	NA	NA	411.74	903.71	1.13	1.14E-05	Up
POX00086	hypothetical protein	NA	NA	NA	138.16	303.30	1.13	7.09E-06	Up
POX00555	hypothetical protein	NA	NA	NA	556.39	74.55	-2.90	3.08E-35	Down
POX01832	hypothetical protein	NA	NA	NA	12342.93	27108.27	1.14	2.44E-07	Up
POX05062	hypothetical protein	NA	NA	NA	188.87	414.98	1.14	3.14E-07	Up
POX04096	hypothetical protein	NA	NA	NA	4549.30	10018.85	1.14	1.8E-10	Up
POX09661	hypothetical protein	NA	NA	NA	441.95	974.17	1.14	2.14E-11	Up
POX03860	hypothetical protein	NA	NA	NA	152.15	20.40	-2.90	5.24E-23	Down

POX09354	hypothetical protein	NA	NA	NA	1439.91	193.94	-2.89	3.26E-29	Down
POX04800	hypothetical protein	NA	NA	NA	1029.44	140.46	-2.87	1.29E-30	Down
POX06345	hypothetical protein	NA	NA	NA	345.02	47.61	-2.86	2.01E-23	Down
POX05467	hypothetical protein	NA	NA	NA	217.09	29.99	-2.86	1.33E-21	Down
POX09026	hypothetical protein	NA	NA	NA	914.75	127.77	-2.84	1.14E-56	Down
POX01435	hypothetical protein	NA	NA	NA	601.52	1332.57	1.15	5.55E-06	Up
POX02066	hypothetical protein	NA	NA	NA	429.24	61.81	-2.80	3.37E-39	Down
POX01701	hypothetical protein	NA	CE9	NA	13.22	1.93	-2.77	1.82E-05	Down
POX06987	hypothetical protein	NA	NA	NA	27.38	4.01	-2.77	2.5E-07	Down
POX08902	putative alpha-1,6-mannanase	NA	GH76	NA	32.29	4.73	-2.77	8.3E-08	Down
POX06508	hypothetical protein	NA	NA	NA	76.97	11.40	-2.76	4.07E-13	Down
POX07424	putative chitinase	NA	GH18	NA	6114.69	907.90	-2.75	6.78E-45	Down
POX01470	hypothetical protein	NA	GH18	NA	1843.01	274.85	-2.75	1.67E-35	Down
POX01843	hypothetical protein	NA	NA	NA	283.60	42.65	-2.73	1.29E-24	Down
POX00805	hypothetical protein	NA	NA	NA	3548.17	7867.36	1.15	2.58E-09	Up
POX07644	hypothetical protein	NA	NA	NA	346.15	53.68	-2.69	2.18E-34	Down
POX08943	hypothetical protein	NA	NA	NA	4797.69	10641.16	1.15	4.33E-11	Up
POX04189	hypothetical protein	NA	NA	NA	18960.28	42067.49	1.15	3.99E-12	Up
POX01689	hypothetical protein	NA	NA	NA	72.18	160.42	1.15	2.91E-05	Up
POX01211	hypothetical protein	NA	NA	NA	61.03	9.54	-2.68	3.75E-10	Down
POX08617	hypothetical protein	NA	NA	NA	134.87	299.99	1.15	9.11E-06	Up
POX06226	hypothetical protein	NA	NA	NA	458.49	1021.34	1.16	2.93E-09	Up
POX01286	hypothetical protein	NA	NA	NA	1682.03	3748.63	1.16	1.05E-06	Up
POX07819	hypothetical protein	NA	NA	NA	21.05	46.94	1.16	0.004967	Up
POX08810	hypothetical protein	NA	NA	NA	54.04	8.49	-2.67	3.48E-10	Down
POX09357	hypothetical protein	NA	NA	NA	7826.64	1247.41	-2.65	8.8E-22	Down
POX03306	hypothetical protein	NA	NA	NA	237.94	38.24	-2.64	2.44E-23	Down
POX04838	hypothetical protein	NA	NA	NA	60.42	135.08	1.16	0.000133	Up
POX03797	hypothetical protein	NA	NA	NA	38.31	85.95	1.17	0.002391	Up
POX06627	hypothetical protein	NA	NA	NA	991.64	2226.06	1.17	2.69E-11	Up
POX09006	hypothetical protein	NA	NA	NA	149.54	24.14	-2.63	2.8E-20	Down

POX07913	hypothetical protein	NA	NA	NA	282.29	45.78	-2.62	2.08E-20	Down
POX01184	hypothetical protein	NA	NA	IPR011991:Winged helix repressor DNA-binding	9.23	1.50	-2.62	0.000108	Down
POX09355	hypothetical protein	NA	NA	NA	3128.59	513.67	-2.61	9.56E-23	Down
POX02429	hypothetical protein	NA	NA	NA	570.37	1280.53	1.17	1.26E-09	Up
POX00057	hypothetical protein	NA	NA	IPR003163:DNA-binding, yeast	9.48	21.30	1.17	0.031899	Up
POX01934	putative chitosanase	NA	GH75	NA	9.19	1.52	-2.60	0.000123	Down
POX08136	hypothetical protein	NA	NA	NA	195.03	32.46	-2.59	2.13E-20	Down
POX03459	hypothetical protein	NA	NA	NA	48.96	8.18	-2.58	3.3E-09	Down
POX03973	hypothetical protein	NA	NA	NA	246.94	555.43	1.17	3.49E-09	Up
POX04476	hypothetical protein	NA	NA	NA	213.65	480.82	1.17	0.000163	Up
POX00656	hypothetical protein	NA	NA	NA	1220.03	2747.52	1.17	1.37E-10	Up
POX08443	hypothetical protein	NA	NA	NA	55.62	125.59	1.18	0.00049	Up
POX06581	hypothetical protein	NA	NA	NA	367.76	830.70	1.18	3.94E-09	Up
POX00730	hypothetical protein	NA	NA	NA	126.94	21.34	-2.57	1.82E-15	Down
POX06725	hypothetical protein	NA	NA	NA	329.70	744.77	1.18	8.07E-06	Up
POX08592	hypothetical protein	NA	NA	NA	266.59	45.55	-2.55	9.97E-17	Down
POX07339	hypothetical protein	NA	NA	NA	114.87	19.71	-2.54	3.16E-15	Down
POX07399	putative UDP-Glc:sterol glucosyltransferase	NA	GT1	NA	99.78	17.55	-2.51	6.39E-13	Down
POX02996	hypothetical protein	NA	NA	NA	156.45	28.31	-2.47	4.14E-19	Down
POX08767	hypothetical protein	NA	NA	NA	564.08	103.39	-2.45	6.29E-29	Down
POX06834	hypothetical protein	NA	NA	NA	59.90	11.01	-2.44	2.92E-10	Down
POX03085	hypothetical protein	NA	NA	NA	775.60	144.55	-2.42	1.37E-19	Down
POX05359	hypothetical protein	NA	NA	NA	132.95	24.97	-2.41	1.28E-15	Down
POX04599	hypothetical protein	NA	NA	NA	131.77	25.05	-2.40	3.15E-16	Down
POX01135	hypothetical protein	NA	NA	Winged helix repressor DNA-binding:IPR011991:Winged helix repressor DNA-binding	48.67	9.29	-2.39	1.24E-08	Down
POX05112	hypothetical protein	NA	NA	NA	128.18	24.69	-2.38	1.04E-10	Down
POX01697	hypothetical protein	NA	NA	NA	766.96	152.47	-2.33	2.83E-33	Down
POX09360	hypothetical protein	NA	NA	NA	778.16	155.77	-2.32	7.28E-06	Down

POX08030	hypothetical protein	NA	NA	NA	8599.38	1724.61	-2.32	1.23E-26	Down
POX09580	hypothetical protein	NA	NA	NA	23.70	4.76	-2.32	4.96E-05	Down
POX08990	putative endo-beta-1,4-xylanase	Endo-beta-1,4-xylanase	GH10	NA	19.75	44.63	1.18	0.006813	Up
POX09717	hypothetical protein	NA	NA	NA	53.28	120.74	1.18	0.002381	Up
POX02414	hypothetical protein	NA	NA	NA	10.21	23.16	1.18	0.030612	Up
POX00232	hypothetical protein	NA	NA	NA	21.81	49.64	1.19	0.002758	Up
POX01189	putative alpha-mannosidase	NA	GH47	NA	1877.45	381.97	-2.30	1.51E-38	Down
POX04593	hypothetical protein	NA	NA	NA	34.06	77.52	1.19	0.000827	Up
POX01794	hypothetical protein	NA	NA	NA	127.13	290.13	1.19	5.15E-08	Up
POX05650	hypothetical protein	NA	NA	NA	5681.57	13024.29	1.20	2.63E-07	Up
POX06121	hypothetical protein	NA	NA	NA	342.53	786.18	1.20	6.87E-09	Up
POX08906	hypothetical protein	NA	NA	NA	2048.78	418.02	-2.29	8.02E-29	Down
POX00804	hypothetical protein	NA	NA	NA	11.93	27.38	1.20	0.022819	Up
POX00096	hypothetical protein	NA	NA	NA	38.36	7.95	-2.27	1.27E-06	Down
POX09318	hypothetical protein	NA	NA	IPR007219:Fungal specific transcription factor	41.16	8.59	-2.26	6.04E-07	Down
POX04951	hypothetical protein	NA	NA	NA	922.51	2126.29	1.20	8.21E-09	Up
POX09015	hypothetical protein	NA	NA	NA	598.44	1381.33	1.21	1.34E-08	Up
POX09498	hypothetical protein	NA	NA	NA	24.97	57.65	1.21	0.001255	Up
POX01867	hypothetical protein	NA	NA	NA	94.29	19.72	-2.26	1.15E-10	Down
POX03444	hypothetical protein	NA	NA	NA	17.31	40.16	1.21	0.004299	Up
POX09800	hypothetical protein	NA	NA	IPR006600:Centromere protein B, DNA-binding region//IPR007889:Helix-turn-helix, Psq//IPR009057:Hom	23.82	4.99	-2.26	7.32E-05	Down
POX07904	hypothetical protein	NA	NA	NA	98.73	20.74	-2.25	4.82E-10	Down
POX04079	hypothetical protein	NA	NA	NA	10.29	2.16	-2.25	0.000931	Down
POX09187	hypothetical protein	NA	NA	IPR009071:High mobility group box	83.82	17.62	-2.25	1.32E-09	Down
POX03297	hypothetical protein	NA	NA	NA	138.89	29.41	-2.24	4.13E-14	Down
POX07587	hypothetical protein	NA	NA	NA	65.30	151.66	1.22	3.15E-05	Up
POX00835	hypothetical protein	NA	NA	NA	1925.73	4475.69	1.22	3.2E-11	Up
POX01783	hypothetical protein	NA	NA	NA	11.77	27.37	1.22	0.016633	Up
POX08659	hypothetical protein	NA	NA	NA	982.99	2288.69	1.22	2.36E-08	Up
POX00441	hypothetical protein	NA	NA	NA	879.64	2052.14	1.22	2.77E-05	Up

POX06380	SUN domain-containing protein	NA	GH132	NA	503.76	1176.06	1.22	2.43E-11	Up
POX02067	hypothetical protein	NA	NA	NA	2032.70	431.38	-2.24	9.17E-27	Down
POX07470	putative beta-1,6-N-acetylglucosaminidase	NA	GH20	NA	638.31	1490.75	1.22	6.3E-09	Up
POX07375	putative beta-1,3-glucanase	NA	GH16	NA	2081.18	444.86	-2.23	5.16E-25	Down
POX04737	hypothetical protein	NA	NA	NA	587.90	126.01	-2.22	7.02E-29	Down
POX06829	hypothetical protein	NA	NA	NA	9.87	23.08	1.23	0.025391	Up
POX07854	hypothetical protein	NA	NA	IPR004827:Basic-leucine zipper (bZIP) transcription factor	944.04	2209.35	1.23	1.63E-09	Up
POX01238	hypothetical protein	NA	NA	NA	26587.42	62276.19	1.23	8.14E-13	Up
POX01188	hypothetical protein	NA	NA	NA	17.24	3.75	-2.20	0.000194	Down
POX04525	hypothetical protein	NA	NA	NA	139.84	329.20	1.24	2.05E-09	Up
POX04214	hypothetical protein	NA	NA	NA	4192.03	9877.84	1.24	2.94E-07	Up
POX05251	hypothetical protein	NA	NA	IPR001138:Fungal transcriptional regulatory protein, N-terminal	150.26	354.32	1.24	1.19E-06	Up
POX07376	chitin binding domain-containing protein	NA	AA5	NA	1155.03	251.52	-2.20	1E-18	Down
POX09323	hypothetical protein	NA	NA	NA	51.24	11.22	-2.19	6.21E-08	Down
POX03075	hypothetical protein	NA	NA	NA	12.86	2.82	-2.19	0.000737	Down
POX03414	hypothetical protein	NA	NA	NA	7.90	18.64	1.24	0.038005	Up
POX03456	hypothetical protein	NA	NA	NA	10.97	2.41	-2.18	0.001058	Down
POX09313	hypothetical protein	NA	NA	NA	15.59	3.43	-2.18	0.000397	Down
POX06242	putative alpha-glucosidase	NA	GH31	NA	326.52	770.44	1.24	1.29E-05	Up
POX04793	hypothetical protein	NA	NA	NA	1641.85	3875.67	1.24	3.5E-08	Up
POX01617	hypothetical protein	NA	NA	NA	1205.09	2850.32	1.24	4.03E-08	Up
POX06064	hypothetical protein	NA	NA	NA	192.62	42.85	-2.17	6.73E-17	Down
POX09311	hypothetical protein	NA	NA	NA	31.43	7.16	-2.13	1.78E-05	Down
POX06221	hypothetical protein	NA	NA	NA	3907.51	9256.11	1.24	6.68E-14	Up
POX02699	hypothetical protein	NA	NA	NA	68.94	163.42	1.25	0.000104	Up
POX00873	hypothetical protein	NA	NA	NA	157.12	36.00	-2.13	1.88E-13	Down
POX09759	hypothetical protein	NA	NA	NA	97.94	232.40	1.25	0.000307	Up
POX03298	hypothetical protein	NA	NA	NA	10.38	2.38	-2.13	0.001542	Down
POX08652	hypothetical protein	NA	NA	NA	85.36	19.57	-2.12	8.09E-11	Down
POX00250	hypothetical protein	NA	NA	NA	886.02	2104.55	1.25	2.24E-07	Up

POX08897	putative cellulose monooxygenase	Cellulose	CBM1;AA9	NA	5.65	1.30	-2.12	0.002803	Down
POX08868	hypothetical protein	NA	NA	NA	85.41	203.32	1.25	4.51E-06	Up
POX03815	hypothetical protein	NA	NA	NA	11.13	2.58	-2.11	0.001911	Down
POX05530	hypothetical protein	NA	NA	IPR007219:Fungal specific transcription factor	94.62	22.06	-2.10	1.2E-10	Down
POX05611	hypothetical protein	NA	NA	NA	86.26	20.14	-2.10	1.82E-10	Down
POX06915	hypothetical protein	NA	NA	NA	857.69	202.25	-2.08	8.14E-22	Down
POX00917	hypothetical protein	NA	NA	NA	1783.11	4246.69	1.25	5.17E-11	Up
POX02831	hypothetical protein	NA	NA	NA	34.53	8.15	-2.08	7.2E-06	Down
POX02830	hypothetical protein	NA	NA	NA	27.49	6.56	-2.07	0.000142	Down
POX04919	hypothetical protein	NA	NA	NA	14.77	3.53	-2.06	0.001121	Down
POX00439	hypothetical protein	NA	NA	NA	16.32	39.06	1.26	0.00462	Up
POX04927	hypothetical protein	NA	NA	NA	24.07	5.80	-2.05	0.000113	Down
POX04083	hypothetical protein	NA	NA	NA	17.53	41.98	1.26	0.004286	Up
POX02157	hypothetical protein	NA	NA	NA	902.67	2164.09	1.26	1.02E-08	Up
POX07775	hypothetical protein	NA	NA	NA	306.55	74.12	-2.05	3.57E-18	Down
POX03252	hypothetical protein	NA	NA	NA	213.49	512.61	1.26	5.58E-08	Up
POX05511	hypothetical protein	NA	NA	NA	409.13	982.63	1.26	4.49E-13	Up
POX01799	hypothetical protein	NA	NA	NA	118.02	28.57	-2.05	5.86E-12	Down
POX04352	hypothetical protein	NA	NA	NA	24.42	58.67	1.26	0.000833	Up
POX05132	putative cutinase	NA	CE5	NA	1827.61	4392.12	1.26	2.56E-07	Up
POX03707	hypothetical protein	NA	NA	NA	177.16	43.19	-2.04	2.11E-15	Down
POX08831	hypothetical protein	NA	NA	NA	875.39	214.50	-2.03	3.57E-18	Down
POX07622	hypothetical protein	NA	NA	NA	39.11	9.60	-2.03	2.92E-05	Down
POX03104	hypothetical protein	NA	NA	NA	1093.79	269.13	-2.02	4.53E-17	Down
POX04373	hypothetical protein	NA	NA	NA	100.49	24.78	-2.02	1.03E-09	Down
POX05652	hypothetical protein	NA	NA	NA	64.00	15.87	-2.01	4.47E-08	Down
POX00759	putative beta-mannosidase	beta-mannosidase	GH2	NA	187.81	46.67	-2.01	6.31E-15	Down
POX03408	hypothetical protein	NA	NA	NA	81.87	20.41	-2.00	1.96E-08	Down
POX03879	hypothetical protein	NA	NA	NA	27.90	7.01	-1.99	0.000157	Down
POX05740	hypothetical protein	NA	NA	NA	1201.59	2891.00	1.27	7.45E-11	Up
POX09601	hypothetical protein	NA	NA	NA	5.90	14.21	1.27	0.045858	Up
POX06366	hypothetical protein	NA	NA	NA	264.54	66.78	-1.99	6.98E-18	Down
POX01926	hypothetical protein	NA	NA	NA	88.36	22.34	-1.98	3.15E-07	Down
POX07894	hypothetical protein	NA	NA	NA	461.86	1112.59	1.27	2.18E-06	Up

POX01796	putative lipopolysaccharide beta-1,4-N-acetylgalactosaminyltransferase	NA	GT25	NA	328.77	792.25	1.27	3.49E-09	Up
POX05360	hypothetical protein	NA	NA	NA	12032.06	3047.87	-1.98	2.36E-18	Down
POX04074	hypothetical protein	NA	NA	NA	731.70	1763.59	1.27	1.14E-10	Up
POX04392	hypothetical protein	NA	NA	NA	253.08	64.22	-1.98	3.17E-15	Down
POX00771	hypothetical protein	NA	NA	NA	27.40	66.15	1.27	0.000409	Up
POX01417	putative endo-beta-1,4-xylanase	Endo-beta-1,4-xylanase	GH11	NA	161.40	390.27	1.27	3.64E-05	Up
POX09781	hypothetical protein	NA	NA	NA	20.25	5.14	-1.98	0.000619	Down
POX04868	hypothetical protein	NA	NA	NA	154.41	39.75	-1.96	1.06E-10	Down
POX09743	hypothetical protein	NA	NA	NA	15.40	4.01	-1.94	0.001451	Down
POX04869	hypothetical protein	NA	NA	NA	50.09	13.20	-1.92	1.25E-06	Down
POX05540	putative alpha-L-arabinofuranosidase	beta-xylosidase/alpha-L-arabinofuranosidase	GH62	NA	25.45	61.66	1.28	0.00043	Up
POX02187	hypothetical protein	NA	NA	NA	12.56	30.49	1.28	0.010039	Up
POX04928	hypothetical protein	NA	NA	NA	54.02	14.28	-1.92	3.02E-06	Down
POX05240	putative alpha-L-arabinofuranosidase	beta-xylosidase/alpha-L-arabinofuranosidase	GH117;GH43	NA	22.82	6.03	-1.92	0.000375	Down
POX00302	hypothetical protein	NA	NA	NA	149.08	362.08	1.28	2.73E-10	Up
POX05425	hypothetical protein	NA	NA	NA	29.55	71.97	1.28	0.000333	Up
POX08627	hypothetical protein	NA	NA	NA	59.42	15.77	-1.91	2.78E-07	Down
POX03782	hypothetical protein	NA	NA	NA	121.12	32.37	-1.90	5.12E-10	Down
POX08236	hypothetical protein	NA	NA	NA	9.34	22.75	1.28	0.024434	Up
POX05500	hypothetical protein	NA	CE10	NA	69.27	168.74	1.28	3.01E-05	Up
POX03233	hypothetical protein	NA	NA	NA	139.03	37.32	-1.90	4.28E-11	Down
POX00005	hypothetical protein	NA	NA	NA	8.40	20.49	1.29	0.033255	Up
POX07084	putative UDP-Xyl: (mannosyl) glucuronoxylomannan/galactoxylomannan beta-1, 2-	NA	GT90	NA	19.14	46.70	1.29	0.001666	Up
POX01381	chitin binding domain-containing protein	NA	AA11	NA	86.46	23.30	-1.89	1.22E-07	Down
POX01017	hypothetical protein	NA	NA	NA	856.69	2092.01	1.29	1.45E-09	Up
POX00079	hypothetical protein	NA	NA	NA	48.37	118.27	1.29	0.000106	Up
POX01704	hypothetical protein	NA	NA	NA	124.77	33.95	-1.88	1.61E-08	Down
POX07884	hypothetical protein	NA	NA	NA	228.19	62.32	-1.87	1.42E-13	Down
POX05628	hypothetical protein	NA	NA	NA	214.74	527.38	1.30	6.71E-10	Up

POX01283	hypothetical protein	NA	NA	NA	2818.08	6940.33	1.30	2.5E-09	Up
POX02111	hypothetical protein	NA	NA	NA	121.88	300.69	1.30	2.73E-08	Up
POX07232	hypothetical protein	NA	NA	NA	18.25	5.00	-1.87	0.001251	Down
POX07621	hypothetical protein	NA	NA	NA	32.49	8.93	-1.86	0.000123	Down
POX04446	hypothetical protein	NA	NA	NA	346.53	856.55	1.31	1.22E-11	Up
POX06545	putative beta-galactosidase	NA	GH35	NA	72.23	178.84	1.31	1.04E-06	Up
POX00867	hypothetical protein	NA	NA	NA	592.10	163.31	-1.86	9.51E-20	Down
POX02938	hypothetical protein	NA	NA	NA	153.49	42.41	-1.86	2.57E-11	Down
POX01930	hypothetical protein	NA	NA	NA	45.81	113.44	1.31	4.56E-05	Up
POX07374	hypothetical protein	NA	NA	NA	5530.40	1545.89	-1.84	5.61E-17	Down
POX04781	hypothetical protein	NA	NA	NA	3437.79	962.50	-1.84	2.69E-16	Down
POX05266	hypothetical protein	NA	AA1	NA	147.61	365.67	1.31	2.54E-10	Up
POX05306	hypothetical protein	NA	NA	NA	72.05	20.26	-1.83	3.03E-05	Down
POX07602	hypothetical protein	NA	NA	NA	69.35	19.59	-1.82	9.47E-07	Down
POX01651	hypothetical protein	NA	NA	NA	625.48	176.76	-1.82	3.07E-13	Down
POX04292	hypothetical protein	NA	NA	NA	412.90	117.06	-1.82	3.51E-15	Down
POX08776	hypothetical protein	NA	NA	NA	919.98	261.74	-1.81	1.29E-18	Down
POX08613	hypothetical protein	NA	NA	NA	101.28	28.85	-1.81	2.93E-09	Down
POX08877	hypothetical protein	NA	NA	NA	18.95	5.40	-1.81	0.001916	Down
POX02048	hypothetical protein	NA	NA	NA	628.21	178.97	-1.81	5.97E-22	Down
POX05038	hypothetical protein	NA	NA	NA	121.72	301.77	1.31	3.39E-07	Up
POX06602	hypothetical protein	NA	AA7	NA	724.82	206.54	-1.81	3.74E-24	Down
POX06626	TPA: COPII vesicles protein Yip3	NA	NA	NA	119.12	295.57	1.31	3.76E-07	Up
POX07387	Major facilitator superfamily domain	NA	NA	NA	89.65	25.59	-1.81	2.87E-08	Down
POX05055	hypothetical protein	NA	NA	NA	96.53	27.55	-1.81	8.87E-08	Down
POX05572	hypothetical protein	NA	NA	NA	334.80	95.58	-1.81	1.19E-16	Down
POX02012	hypothetical protein	NA	NA	NA	654.36	187.63	-1.80	2.68E-23	Down
POX08455	hypothetical protein	NA	NA	NA	3565.18	8878.10	1.32	2.02E-12	Up
POX07619	hypothetical protein	NA	NA	NA	616.32	176.82	-1.80	5.59E-17	Down
POX09590	hypothetical protein	NA	NA	NA	22.82	56.86	1.32	0.008087	Up
POX08753	hypothetical protein	NA	NA	NA	151.49	377.43	1.32	8.73E-08	Up
POX07240	hypothetical protein	NA	NA	NA	45.19	112.92	1.32	2.53E-06	Up
POX06183	hypothetical protein	NA	NA	NA	43.92	110.71	1.33	5.19E-06	Up
POX00705	hypothetical protein	NA	NA	NA	63.61	18.28	-1.80	2.39E-06	Down
POX06590	hypothetical protein	NA	NA	NA	1168.34	335.96	-1.80	1.22E-17	Down
POX02986	hypothetical protein	NA	NA	NA	372.60	943.18	1.34	1.6E-15	Up
POX01904	hypothetical protein	NA	NA	NA	234.68	594.28	1.34	2.85E-11	Up

POX06661	hypothetical protein	NA	NA	NA	38.76	98.19	1.34	0.000108	Up
POX03936	hypothetical protein	NA	NA	NA	332.60	95.94	-1.79	2.7E-17	Down
POX03870	hypothetical protein	NA	NA	NA	10.01	2.89	-1.79	0.008448	Down
POX01236	hypothetical protein	NA	NA	NA	821.80	2088.63	1.35	6.29E-08	Up
POX03617	hypothetical protein	NA	NA	NA	1481.11	430.80	-1.78	9.11E-18	Down
POX00706	hypothetical protein	NA	NA	NA	423.07	124.92	-1.76	2.55E-16	Down
POX09578	hypothetical protein	NA	CE10;CE1	NA	435.71	128.82	-1.76	1.1E-14	Down
POX02792	hypothetical protein	NA	NA	NA	401.59	118.90	-1.76	3.79E-13	Down
POX06446	hypothetical protein	NA	NA	NA	1052.71	2675.88	1.35	4.43E-06	Up
POX00562	hypothetical protein	NA	NA	NA	148.85	44.09	-1.76	4.01E-09	Down
POX01987	hypothetical protein	NA	NA	NA	31.84	9.44	-1.75	0.000371	Down
POX05794	hypothetical protein	NA	NA	NA	21.00	6.23	-1.75	0.002158	Down
POX03346	hypothetical protein	NA	NA	NA	5.12	1.53	-1.75	0.01825	Down
POX04084	hypothetical protein	NA	NA	NA	1686.25	4294.95	1.35	8.12E-09	Up
POX03889	putative alpha-glucosidase	NA	GH31	NA	3212.94	8188.38	1.35	3.02E-06	Up
POX04390	putative beta-1,3-glucanosyltransglycosylase	NA	CBM43;GH72	NA	2917.92	872.97	-1.74	3.68E-22	Down
POX07212	hypothetical protein	NA	NA	NA	353.55	901.33	1.35	1.51E-14	Up
POX04465	hypothetical protein	NA	NA	IPR001138:Fungal transcriptional regulatory protein, N-terminal	63.40	161.85	1.35	2.85E-07	Up
POX06603	hypothetical protein	NA	NA	NA	248.24	75.17	-1.72	1.14E-09	Down
POX05538	hypothetical protein	NA	NA	NA	1385.17	419.51	-1.72	5.29E-12	Down
POX08764	hypothetical protein	NA	NA	NA	162.17	49.22	-1.72	1.51E-08	Down
POX09346	hypothetical protein	NA	NA	NA	10.07	3.06	-1.72	0.014651	Down
POX02014	hypothetical protein	NA	NA	NA	76.92	23.46	-1.71	4.93E-07	Down
POX02337	hypothetical protein	NA	NA	NA	7.65	19.57	1.35	0.020021	Up
POX02512	hypothetical protein	NA	NA	NA	116.64	298.50	1.36	0.000166	Up
POX08746	hypothetical protein	NA	NA	NA	198.91	60.70	-1.71	1.48E-06	Down
POX05707	hypothetical protein	NA	NA	NA	24.32	7.45	-1.71	0.001749	Down
POX09240	hypothetical protein	NA	NA	NA	347.02	888.53	1.36	1.32E-14	Up
POX09335	hypothetical protein	NA	NA	NA	1092.84	2816.90	1.37	8.07E-12	Up
POX00885	hypothetical protein	NA	NA	NA	8.32	21.47	1.37	0.020311	Up
POX03281	hypothetical protein	NA	NA	NA	90.94	28.04	-1.70	3.81E-06	Down
POX05777	hypothetical protein	NA	NA	NA	5.63	14.54	1.37	0.026984	Up
POX01833	putative alpha-mannosyltransferase	NA	GT71	NA	18.50	47.80	1.37	0.002333	Up

POX07677	hypothetical protein	NA	NA	NA	115.63	299.32	1.37	2.65E-09	Up
POX00288	hypothetical protein	NA	NA	NA	28.62	74.09	1.37	6.44E-05	Up
POX09081	hypothetical protein	NA	NA	NA	5.98	15.59	1.38	0.026438	Up
POX05271	hypothetical protein	NA	NA	NA	717.18	1877.67	1.39	3.5E-15	Up
POX07750	hypothetical protein	NA	NA	NA	16.25	5.02	-1.70	0.005792	Down
POX06177	hypothetical protein	NA	NA	NA	786.28	2058.93	1.39	3.73E-14	Up
POX03749	hypothetical protein	NA	NA	NA	1142.03	2990.59	1.39	4.85E-17	Up
POX05089	hypothetical protein	NA	NA	NA	40.95	107.30	1.39	1.07E-06	Up
POX07177	hypothetical protein	NA	NA	NA	271.83	712.31	1.39	8.94E-08	Up
POX06976	hypothetical protein	NA	NA	NA	21.66	56.79	1.39	0.000726	Up
POX00933	hypothetical protein	NA	NA	NA	117.29	307.92	1.39	9.47E-07	Up
POX09699	hypothetical protein	NA	NA	NA	918.13	285.04	-1.69	1.8E-19	Down
POX08814	hypothetical protein	NA	NA	NA	24.05	63.18	1.39	0.000247	Up
POX02261	hypothetical protein	NA	NA	IPR001138:Fungal transcriptional regulatory protein, N-terminal	18.81	49.46	1.39	0.000459	Up
POX08399	hypothetical protein	NA	NA	NA	152.03	47.51	-1.68	1.91E-10	Down
POX02448	hypothetical protein	NA	NA	NA	26.48	8.29	-1.68	0.000935	Down
POX08818	hypothetical protein	NA	NA	NA	10.42	27.41	1.40	0.006356	Up
POX02290	hypothetical protein	NA	NA	IPR009057:Homeodomain-like	5746.25	15193.66	1.40	6.6E-09	Up
POX07030	hypothetical protein	NA	NA	NA	3.69	9.76	1.40	0.045727	Up
POX00252	hypothetical protein	NA	NA	NA	6292.17	16649.93	1.40	2.32E-08	Up
POX05481	hypothetical protein	NA	NA	NA	188.33	498.97	1.41	8.3E-08	Up
POX06583	hypothetical protein	NA	NA	NA	52.55	139.24	1.41	3.44E-05	Up
POX08646	hypothetical protein	NA	NA	NA	1468.11	3896.44	1.41	1.21E-10	Up
POX09326	hypothetical protein	NA	NA	NA	5.79	1.82	-1.67	0.024559	Down
POX06301	putative alpha-mannosidase	NA	GH47	NA	494.07	155.00	-1.67	3.29E-11	Down
POX00976	hypothetical protein	NA	NA	NA	6893.40	18365.51	1.41	9.51E-15	Up
POX01325	hypothetical protein	NA	NA	NA	3.53	1.11	-1.67	0.023635	Down
POX02377	hypothetical protein	NA	NA	NA	138.51	43.77	-1.66	3.62E-10	Down
POX04504	hypothetical protein	NA	NA	NA	422.73	1127.49	1.42	9.74E-13	Up
POX09812	hypothetical protein	NA	NA	NA	11.21	3.57	-1.65	0.014042	Down
POX06337	hypothetical protein	NA	NA	NA	149.81	47.93	-1.64	5.25E-10	Down
POX09554	hypothetical protein	NA	NA	NA	6.01	16.07	1.42	0.024182	Up
POX03284	hypothetical protein	NA	NA	NA	114.64	307.30	1.42	1.13E-09	Up

POX05351	hypothetical protein	NA	NA	NA	201.35	542.52	1.43	2.78E-10	Up
POX09336	hypothetical protein	NA	NA	NA	1535.57	4137.62	1.43	7.35E-12	Up
POX03508	hypothetical protein	NA	NA	NA	701.61	1891.61	1.43	1.94E-13	Up
POX04190	hypothetical protein	NA	NA	NA	203.83	549.91	1.43	4.17E-14	Up
POX08489	hypothetical protein	NA	NA	NA	270.91	731.18	1.43	4.33E-09	Up
POX07912	hypothetical protein	NA	NA	NA	3.42	1.10	-1.64	0.026774	Down
POX08340	hypothetical protein	NA	NA	IPR007219:Fungal specific transcription factor	187.80	60.40	-1.64	5.73E-11	Down
POX03379	hypothetical protein	NA	NA	NA	8.82	23.88	1.44	0.011748	Up
POX01679	hypothetical protein	NA	NA	NA	16.22	5.22	-1.64	0.006518	Down
POX06980	hypothetical protein	NA	NA	NA	1981.85	5391.28	1.44	1.71E-11	Up
POX05037	hypothetical protein	NA	NA	NA	16.18	44.04	1.44	0.002241	Up
POX07378	putative cutinase	NA	CE5	NA	44.70	121.71	1.45	0.048722	Up
POX06621	hypothetical protein	NA	NA	NA	865.36	278.69	-1.63	1.65E-13	Down
POX01475	hypothetical protein	NA	NA	NA	77.45	24.97	-1.63	6.16E-07	Down
POX07896	hypothetical protein	NA	NA	NA	11236.18	30629.80	1.45	2.18E-07	Up
POX05220	hypothetical protein	NA	NA	NA	241.08	77.73	-1.63	1.71E-10	Down
POX05017	hypothetical protein	NA	NA	NA	469.90	1284.18	1.45	5.29E-13	Up
POX05053	hypothetical protein	NA	NA	NA	16.11	5.22	-1.63	0.008485	Down
POX07366	hypothetical protein	NA	NA	NA	330.53	107.61	-1.62	6.08E-10	Down
POX08562	hypothetical protein	NA	NA	NA	8960.46	24489.92	1.45	4.29E-09	Up
POX07539	hypothetical protein	NA	NA	NA	1929.62	629.26	-1.62	1.54E-11	Down
POX09056	putative alpha-mannosidase	NA	GH47	NA	644.31	210.36	-1.61	4.52E-17	Down
POX03283	hypothetical protein	NA	CE10	NA	34.66	94.88	1.45	3.4E-06	Up
POX06008	hypothetical protein	NA	NA	NA	538.46	176.30	-1.61	1.94E-13	Down
POX02605	hypothetical protein	NA	NA	NA	5983.26	1959.68	-1.61	2.11E-07	Down
POX02121	hypothetical protein	NA	NA	NA	7.08	19.42	1.46	0.020385	Up
POX03432	hypothetical protein	NA	NA	NA	159.55	52.32	-1.61	3.62E-10	Down
POX05826	hypothetical protein	NA	NA	NA	13.74	37.80	1.46	0.000982	Up
POX08103	hypothetical protein	NA	NA	NA	38.92	12.82	-1.60	0.001128	Down
POX09083	hypothetical protein	NA	NA	IPR001138:Fungal transcriptional regulatory protein, N-terminal//IPR007219: Fungal specific transcription factor	49.42	136.38	1.46	1.22E-07	Up
POX03620	hypothetical protein	NA	NA	NA	7.39	2.44	-1.60	0.027553	Down
POX01696	hypothetical protein	NA	NA	NA	1187.83	391.82	-1.60	2.23E-22	Down
POX09319	hypothetical protein	NA	NA	NA	9.65	3.19	-1.60	0.022447	Down

POX03507	hypothetical protein	NA	NA	NA	336.20	930.01	1.47	6.47E-15	Up
POX09606	hypothetical protein	NA	NA	NA	23.07	63.98	1.47	6.41E-05	Up
POX05344	hypothetical protein	NA	NA	NA	14.48	4.80	-1.59	0.010431	Down
POX02623	hypothetical protein	NA	NA	NA	97.09	32.34	-1.59	0.000113	Down
POX03234	hypothetical protein	NA	NA	NA	174.58	58.19	-1.59	5.84E-10	Down
POX00255	hypothetical protein	NA	NA	NA	262.07	727.30	1.47	1.34E-13	Up
POX06283	hypothetical protein	NA	NA	NA	5.42	1.81	-1.58	0.036491	Down
POX00669	hypothetical protein	NA	NA	NA	4648.94	1553.55	-1.58	4.95E-13	Down
POX04860	hypothetical protein	NA	NA	IPR001356:Homeobox//IPR009057:Homeodomain-like	496.74	1381.25	1.48	1.78E-10	Up
POX07383	hypothetical protein	NA	NA	NA	1087.78	3025.55	1.48	7.58E-08	Up
POX04577	hypothetical protein	NA	NA	NA	14.44	4.83	-1.58	0.010542	Down
POX03858	hypothetical protein	NA	NA	NA	43.04	14.43	-1.58	0.000372	Down
POX04823	hypothetical protein	NA	NA	NA	248.58	83.41	-1.58	3.54E-12	Down
POX01914	putative alpha-L-arabinofuranosidase	beta-xylosidase/alpha-L-arabinofuranosidase	CBM42;GH54	NA	106.66	35.87	-1.57	1.28E-05	Down
POX03954	hypothetical protein	NA	NA	IPR001138:Fungal transcriptional regulatory protein, N-	74.33	25.01	-1.57	4.52E-06	Down
POX04922	hypothetical protein	NA	NA	NA	1345.26	3743.45	1.48	2.25E-17	Up
POX07962	hypothetical protein	NA	NA	NA	2099.35	5858.65	1.48	7.08E-19	Up
POX00105	putative exo-beta-1,3-glucanase	NA	GH55	NA	41.05	115.36	1.49	3.03E-07	Up
POX03501	hypothetical protein	NA	NA	NA	121.16	340.54	1.49	1.71E-12	Up
POX05871	hypothetical protein	NA	NA	NA	9.52	26.82	1.50	0.002551	Up
POX02781	hypothetical protein	NA	NA	NA	41.92	118.56	1.50	6.15E-07	Up
POX06178	hypothetical protein	NA	NA	NA	11.84	33.58	1.50	0.00353	Up
POX00641	hypothetical protein	NA	NA	NA	15.03	5.06	-1.57	0.01377	Down
POX07802	putative beta-fructofuranosidase	NA	GH32	NA	47.37	16.03	-1.56	0.000189	Down
POX06953	hypothetical protein	NA	NA	IPR001138:Fungal transcriptional regulatory protein, N-terminal//IPR007219: Fungal specific transcription factor	121.78	346.05	1.51	4.02E-09	Up
POX05477	hypothetical protein	NA	NA	NA	1160.46	3303.49	1.51	3.92E-11	Up
POX08867	hypothetical protein	NA	NA	NA	12.93	36.86	1.51	0.002427	Up
POX04388	hypothetical protein	NA	NA	NA	13.14	37.47	1.51	0.002479	Up

POX08356	hypothetical protein	NA	NA	NA	14.54	4.92	-1.56	0.013059	Down
POX06876	hypothetical protein	NA	NA	NA	1917.64	5470.80	1.51	2.69E-13	Up
POX09681	hypothetical protein	NA	NA	NA	5.31	1.81	-1.56	0.041014	Down
POX04037	hypothetical protein	NA	NA	NA	10.18	3.48	-1.55	0.026269	Down
POX02007	hypothetical protein	NA	NA	NA	18.28	6.26	-1.54	0.008929	Down
POX02853	hypothetical protein	NA	NA	NA	1925.44	660.50	-1.54	1.74E-08	Down
POX05576	hypothetical protein	NA	NA	NA	1.03	2.93	1.51	0.042547	Up
POX02475	hypothetical protein	NA	NA	NA	13.24	37.81	1.51	0.004833	Up
POX01637	hypothetical protein	NA	NA	NA	16.90	5.82	-1.54	0.009609	Down
POX04501	hypothetical protein	NA	NA	NA	605.23	1729.26	1.51	3.58E-10	Up
POX01764	hypothetical protein	NA	NA	NA	1540.94	4418.25	1.52	0.00184	Up
POX03762	hypothetical protein	NA	AA8	NA	18.59	53.32	1.52	0.000237	Up
POX03581	hypothetical protein	NA	NA	NA	1.04	3.00	1.52	0.040687	Up
POX08406	hypothetical protein	NA	NA	NA	20.15	58.10	1.53	0.000103	Up
POX01248	hypothetical protein	NA	NA	NA	1659.69	4788.86	1.53	2.96E-20	Up
POX05861	hypothetical protein	NA	NA	NA	21.43	61.85	1.53	0.000628	Up
POX02209	hypothetical protein	NA	NA	NA	2.34	6.77	1.53	0.043753	Up
POX03958	hypothetical protein	NA	NA	NA	20.74	7.14	-1.54	0.005208	Down
POX01224	hypothetical protein	NA	NA	NA	10.92	31.71	1.54	0.001594	Up
POX04375	hypothetical protein	NA	NA	NA	98.21	33.85	-1.54	2.95E-05	Down
POX01554	hypothetical protein	NA	NA	NA	597.36	1736.39	1.54	9.06E-15	Up
POX00379	hypothetical protein	NA	NA	NA	14.88	5.14	-1.53	0.013376	Down
POX03182	hypothetical protein	NA	NA	NA	214.16	622.94	1.54	3.22E-10	Up
POX05515	hypothetical protein	NA	NA	NA	1762.40	609.58	-1.53	2.48E-10	Down
POX03871	hypothetical protein	NA	NA	NA	15.19	5.27	-1.53	0.014514	Down
POX01939	hypothetical protein	NA	NA	NA	3.31	9.65	1.54	0.032568	Up
POX06772	hypothetical protein	NA	NA	NA	78.17	228.68	1.55	3.47E-10	Up
POX02999	hypothetical protein	NA	NA	NA	13.73	40.21	1.55	0.000633	Up
POX04686	hypothetical protein	NA	GH128	NA	2018.62	701.07	-1.53	3.15E-14	Down
POX08758	hypothetical protein	NA	AA3;AA8	NA	1.52	4.46	1.55	0.040738	Up
POX05834	hypothetical protein	NA	NA	NA	281.88	829.03	1.56	1.11E-16	Up
POX07975	hypothetical protein	NA	NA	NA	392.97	1156.42	1.56	4.11E-12	Up
POX02015	hypothetical protein	NA	NA	NA	41.61	14.46	-1.52	0.00049	Down
POX05269	hypothetical protein	NA	NA	NA	607.70	1789.26	1.56	4.32E-19	Up
POX01218	putative acetyl xylan esterase	Acetyl	CBM1;CE1	NA	8.47	24.95	1.56	0.002235	Up
POX06102	hypothetical protein	NA	NA	NA	2.14	6.31	1.56	0.037441	Up
POX09339	hypothetical protein	NA	NA	NA	159.75	472.75	1.57	1.03E-09	Up
POX01675	aromatic prenyltransferase	NA	NA	NA	9.06	3.15	-1.52	0.034203	Down

POX06153	hypothetical protein	NA	NA	NA	401.98	140.03	-1.52	5.2E-13	Down
POX01319	hypothetical protein	NA	NA	NA	2.95	1.03	-1.52	0.041864	Down
POX09675	hypothetical protein	NA	NA	NA	1161.54	3439.35	1.57	2.05E-15	Up
POX02456	hypothetical protein	NA	NA	NA	103.38	307.30	1.57	2.03E-08	Up
POX07577	hypothetical protein	NA	NA	NA	68.51	204.55	1.58	2.24E-07	Up
POX02026	hypothetical protein	NA	NA	NA	14.96	5.23	-1.52	0.018881	Down
POX05587	cellobiohydrolase CBHI/Cel7A-2	Cellobiohydrolase	CBM1;GH7	NA	2.41	7.20	1.58	0.030778	Up
POX00769	hypothetical protein	NA	NA	NA	24.36	72.95	1.58	7.55E-06	Up
POX06021	hypothetical protein	NA	NA	NA	2098.32	6286.80	1.58	2.69E-16	Up
POX02798	hypothetical protein	NA	NA	NA	65.55	198.15	1.60	8.76E-11	Up
POX08410	hypothetical protein	NA	NA	NA	43.72	15.33	-1.51	0.00065	Down
POX03669	hypothetical protein	NA	NA	NA	7.85	2.75	-1.51	0.037128	Down
POX01000	hypothetical protein	NA	NA	NA	299.09	904.71	1.60	8.67E-20	Up
POX08904	hypothetical protein	NA	NA	NA	278.64	98.20	-1.50	2.92E-11	Down
POX04353	hypothetical protein	NA	NA	NA	30.98	93.73	1.60	5.12E-06	Up
POX03955	hypothetical protein	NA	NA	NA	8.74	3.10	-1.50	0.037832	Down
POX02064	hypothetical protein	NA	NA	NA	61.72	22.10	-1.48	5.03E-05	Down
POX09267	hypothetical protein	NA	NA	NA	61.02	21.89	-1.48	7.55E-05	Down
POX01501	hypothetical protein	NA	NA	NA	16.32	49.39	1.60	7.96E-05	Up
POX06453	hypothetical protein	NA	NA	NA	85.44	258.57	1.60	7.49E-09	Up
POX04779	hypothetical protein	NA	NA	NA	186.15	563.44	1.60	3.39E-10	Up
POX09222	hypothetical protein	NA	NA	NA	41.68	126.40	1.60	3.11E-06	Up
POX02008	hypothetical protein	NA	NA	NA	42194.79	15138.60	-1.48	6.97E-21	Down
POX08894	hypothetical protein	NA	NA	NA	131.43	47.16	-1.48	6.18E-07	Down
POX01390	hypothetical protein	NA	CE1	NA	18.73	57.01	1.61	0.000269	Up
POX00392	hypothetical protein	NA	NA	NA	1224.91	3742.36	1.61	1.47E-17	Up
POX01979	hypothetical protein	NA	NA	NA	5.20	15.95	1.62	0.007549	Up
POX08278	hypothetical protein	NA	NA	NA	1.43	4.46	1.63	0.02971	Up
POX05265	hypothetical protein	NA	NA	NA	124.32	386.24	1.64	1.81E-12	Up
POX05339	hypothetical protein	NA	NA	NA	407.04	1266.59	1.64	4.58E-23	Up
POX09674	hypothetical protein	NA	NA	NA	241.83	753.93	1.64	2.6E-14	Up
POX08667	hypothetical protein	NA	NA	NA	4.39	13.70	1.64	0.012464	Up

POX00397	hypothetical protein	NA	NA	NA	265.18	830.16	1.65	4.49E-13	Up
POX06074	hypothetical protein	NA	NA	NA	260.24	817.94	1.65	6.6E-15	Up
POX07099	hypothetical protein	NA	NA	IPR001005:Myb, DNA-binding//IPR009057:H	61.25	192.63	1.65	2.67E-12	Up
POX07049	hypothetical protein	NA	NA	NA	705.46	2219.49	1.65	4.61E-23	Up
POX01615	putative GDP-Man: alpha-1,3-mannosyltransferase	NA	GT69	NA	7.17	22.64	1.66	0.002427	Up
POX06830	hypothetical protein	NA	NA	NA	2.98	9.46	1.67	0.018903	Up
POX00714	hypothetical protein	NA	NA	NA	1085.10	3444.64	1.67	8.64E-10	Up
POX02711	hypothetical protein	NA	NA	NA	38.47	122.21	1.67	2.68E-07	Up
POX07364	hypothetical protein	NA	NA	NA	164.50	523.96	1.67	2.5E-15	Up
POX08685	hypothetical protein	NA	NA	NA	1.85	5.89	1.67	0.02487	Up
POX09566	hypothetical protein	NA	NA	NA	1.80	5.75	1.68	0.024539	Up
POX05321	hypothetical protein	NA	NA	NA	16.21	52.15	1.69	9.77E-05	Up
POX05077	hypothetical protein	NA	NA	NA	5.76	18.59	1.69	0.004914	Up
POX07805	hypothetical protein	NA	NA	NA	90.78	32.65	-1.48	9.18E-06	Down
POX00284	hypothetical protein	NA	NA	NA	61.38	199.03	1.70	3.45E-13	Up
POX06360	hypothetical protein	NA	NA	NA	92.05	298.87	1.70	8.14E-13	Up
POX01777	hypothetical protein	NA	NA	NA	1799.28	647.32	-1.47	4.11E-13	Down
POX04523	hypothetical protein	NA	NA	NA	49.45	17.81	-1.47	0.000205	Down
POX03598	hypothetical protein	NA	NA	NA	62.93	22.77	-1.47	0.000143	Down
POX07428	hypothetical protein	NA	NA	NA	133.58	434.11	1.70	2.5E-13	Up
POX05593	hypothetical protein	NA	NA	NA	3397.92	1230.49	-1.47	4.33E-10	Down
POX02415	hypothetical protein	NA	NA	NA	1.50	4.86	1.70	0.022711	Up
POX02700	hypothetical protein	NA	NA	NA	866.13	2816.89	1.70	3.91E-20	Up
POX08705	hypothetical protein	NA	NA	NA	48.82	17.71	-1.46	0.000407	Down
POX05987	hypothetical protein	NA	NA	NA	109.22	355.83	1.70	4.01E-10	Up
POX06435	hypothetical protein	NA	NA	NA	1778.12	5799.13	1.71	1.17E-18	Up
POX01674	hypothetical protein	NA	NA	NA	84.14	30.54	-1.46	4.66E-06	Down
POX03410	hypothetical protein	NA	NA	NA	26.68	9.69	-1.46	0.004661	Down
POX09101	hypothetical protein	NA	NA	NA	10.54	3.86	-1.45	0.038778	Down
POX06434	hypothetical protein	NA	NA	NA	2.22	7.23	1.71	0.019101	Up
POX09718	hypothetical protein	NA	NA	NA	71.91	235.44	1.71	9.06E-07	Up
POX09713	hypothetical protein	NA	NA	NA	6.22	20.51	1.72	0.006725	Up
POX08433	hypothetical protein	NA	NA	NA	1.50	4.98	1.72	0.020259	Up
POX02188	hypothetical protein	NA	NA	NA	110.54	366.05	1.73	1.21E-15	Up
POX05852	hypothetical protein	NA	AA7	NA	8.31	27.56	1.73	0.001539	Up

POX03449	hypothetical protein	NA	NA	NA	82.04	272.13	1.73	1.77E-13	Up
POX02985	hypothetical protein	NA	NA	NA	824.01	2742.77	1.73	2.81E-24	Up
POX03602	hypothetical protein	NA	NA	NA	714.06	262.59	-1.44	1.19E-16	Down
POX07123	hypothetical protein	NA	NA	NA	140.91	51.88	-1.44	2.67E-08	Down
POX08950	hypothetical protein	NA	NA	NA	369.33	1235.57	1.74	5.17E-22	Up
POX07878	hypothetical protein	NA	NA	NA	9.23	3.40	-1.44	0.041338	Down
POX03932	hypothetical protein	NA	NA	NA	415.95	153.87	-1.43	1.14E-11	Down
POX06551	putative alpha-1,6-mannosyltransferase	NA	GT32	NA	114.09	383.05	1.75	2.54E-15	Up
POX05571	endoglucanase 1	Endo-beta-1,4-glucanase	CBM1;GH7	NA	14.40	48.47	1.75	1.78E-05	Up
POX06689	putative endo-beta-1,4-galactanase	Endo-beta-1,4-galactanase	GH53	NA	16.31	54.94	1.75	8.08E-06	Up
POX04229	alpha-L-rhamnosidase	NA	GH78;CBM67	NA	7.48	25.37	1.76	0.000993	Up
POX00700	hypothetical protein	NA	NA	NA	193.94	71.89	-1.43	1.5E-08	Down
POX03925	hypothetical protein	NA	NA	IPR011991:Winged helix repressor DNA-binding	562.55	1911.11	1.76	1.47E-17	Up
POX08684	hypothetical protein	NA	NA	NA	1.53	5.22	1.77	0.01671	Up
POX08208	hypothetical protein	NA	NA	NA	7.22	24.65	1.77	0.000641	Up
POX02734	hypothetical protein	NA	NA	IPR004827:Basic-leucine zipper (bZIP) transcription factor	240.80	826.18	1.78	1.39E-16	Up
POX06451	hypothetical protein	NA	NA	NA	23.52	8.74	-1.43	0.008198	Down
POX03064	hypothetical protein	NA	NA	NA	9.90	34.01	1.78	9.08E-05	Up
POX01519	hypothetical protein	NA	NA	NA	134.63	50.01	-1.43	8.53E-08	Down
POX09474	hypothetical protein	NA	NA	NA	1.48	5.12	1.78	0.014386	Up
POX03105	hypothetical protein	NA	NA	NA	31.84	11.83	-1.43	0.002245	Down
POX08872	hypothetical protein	NA	NA	NA	164.77	61.40	-1.42	9.47E-06	Down
POX07389	hypothetical protein	NA	NA	NA	1.18	4.05	1.79	0.01466	Up
POX05188	putative chitin glucanosyltransferase	NA	GH16	NA	24.66	9.19	-1.42	0.007025	Down
POX00014	putative chitin synthase	NA	NA	NA	136.56	50.97	-1.42	3.35E-07	Down
POX09626	DNA breaking-rejoining enzyme, catalytic core	NA	NA	NA	68.34	25.57	-1.42	3.95E-05	Down
POX06809	hypothetical protein	NA	NA	NA	736.36	2540.38	1.79	1.88E-21	Up
POX04735	hypothetical protein	NA	NA	NA	239.26	89.60	-1.42	5.92E-06	Down
POX09589	hypothetical protein	NA	NA	NA	2.02	6.98	1.79	0.014905	Up
POX01601	hypothetical protein	NA	NA	NA	97.91	338.47	1.79	2.67E-11	Up

POX09588	hypothetical protein	NA	NA	NA	3.34	11.58	1.79	0.007513	Up
POX07739	hypothetical protein	NA	NA	NA	187.07	649.34	1.80	9.97E-20	Up
POX07238	hypothetical protein	NA	NA	NA	10.77	37.52	1.80	9.44E-05	Up
POX09710	hypothetical protein	NA	NA	NA	11.71	41.40	1.82	4.93E-05	Up
POX06444	hypothetical protein	NA	NA	NA	25.08	88.81	1.82	1.95E-06	Up
POX01933	hypothetical protein	NA	NA	NA	34.66	12.98	-1.42	0.002234	Down
POX01431	hypothetical protein	NA	AA1	NA	5.68	20.15	1.83	0.001165	Up
POX04513	hypothetical protein	NA	NA	IPR001138:Fungal transcriptional regulatory protein, N-terminal	13.46	5.04	-1.42	0.028171	Down
POX09301	hypothetical protein	NA	NA	NA	224.76	84.95	-1.40	9.43E-09	Down
POX02585	hypothetical protein	NA	NA	NA	72.27	256.73	1.83	2.47E-11	Up
POX04565	hypothetical protein	NA	NA	NA	1311.96	496.67	-1.40	8.33E-11	Down
POX05829	hypothetical protein	NA	NA	NA	332.43	1181.01	1.83	7.67E-15	Up
POX09720	hypothetical protein	NA	NA	NA	13.16	47.27	1.85	0.000166	Up
POX09172	hypothetical protein	NA	NA	NA	46.75	17.73	-1.40	0.000563	Down
POX00836	hypothetical protein	NA	NA	NA	28.09	101.29	1.85	1.25E-07	Up
POX03413	hypothetical protein	NA	NA	NA	3.66	13.22	1.85	0.004237	Up
POX08657	hypothetical protein	NA	NA	NA	64.50	24.56	-1.39	0.000128	Down
POX02924	hypothetical protein	NA	NA	NA	10.70	4.08	-1.39	0.043905	Down
POX01614	hypothetical protein	NA	NA	NA	224.95	85.72	-1.39	1.86E-09	Down
POX08460	hypothetical protein	NA	NA	NA	41.31	15.82	-1.38	0.001045	Down
POX05489	hypothetical protein	NA	NA	NA	50.92	19.51	-1.38	0.000762	Down
POX08806	hypothetical protein	NA	NA	NA	47.88	18.35	-1.38	0.001866	Down
POX07392	hypothetical protein	NA	NA	NA	1.27	4.60	1.85	0.0108	Up
POX06207	hypothetical protein	NA	NA	NA	2.62	9.54	1.86	0.006708	Up
POX00556	hypothetical protein	NA	NA	NA	12.49	4.79	-1.38	0.040195	Down
POX04920	putative pectin lyase	Pectate	PL1	NA	12.13	44.29	1.87	1.02E-05	Up
POX08154	hypothetical protein	NA	NA	NA	40.38	15.49	-1.38	0.001445	Down
POX06223	hypothetical protein	NA	NA	NA	107.34	391.93	1.87	2.56E-13	Up
POX05858	hypothetical protein	NA	NA	NA	3.50	12.87	1.88	0.007041	Up
POX08172	hypothetical protein	NA	NA	NA	369.24	142.13	-1.38	6.3E-09	Down
POX05260	hypothetical protein	NA	AA7	NA	283.69	1043.88	1.88	1.93E-28	Up
POX04303	hypothetical protein	NA	NA	NA	274.33	105.70	-1.38	8.76E-11	Down
POX04149	hypothetical protein	NA	NA	NA	1625.90	5998.51	1.88	4.25E-26	Up
POX09108	hypothetical protein	NA	NA	NA	63.77	24.61	-1.37	0.000196	Down

POX00246	hypothetical protein	NA	NA	NA	1635.91	6055.89	1.89	1.08E-18	Up
POX09299	hypothetical protein	NA	NA	NA	3.99	14.81	1.89	0.002288	Up
POX01826	hypothetical protein	NA	NA	NA	14.49	54.48	1.91	3.45E-06	Up
POX09714	hypothetical protein	NA	NA	NA	9.32	35.44	1.93	0.0005	Up
POX04560	hypothetical protein	NA	NA	NA	8.97	34.26	1.93	2.95E-05	Up
POX08072	hypothetical protein	NA	NA	NA	5.30	20.26	1.93	0.001016	Up
POX06033	hypothetical protein	NA	NA	NA	582.76	2226.32	1.93	1.51E-16	Up
POX04630	hypothetical protein	NA	NA	NA	302.22	1159.44	1.94	1.14E-12	Up
POX03524	hypothetical protein	NA	NA	NA	38.17	14.80	-1.37	0.001485	Down
POX03450	hypothetical protein	NA	NA	NA	105.00	403.27	1.94	3.34E-17	Up
POX03670	hypothetical protein	NA	NA	NA	164.04	63.66	-1.37	1.36E-07	Down
POX05853	hypothetical protein	NA	NA	NA	12.41	47.93	1.95	5.73E-05	Up
POX00837	hypothetical protein	NA	NA	NA	9.32	36.12	1.95	3.13E-05	Up
POX00542	hypothetical protein	NA	NA	NA	160.51	623.35	1.96	1.58E-15	Up
POX08382	hypothetical protein	NA	NA	NA	323.85	1258.62	1.96	4.2E-29	Up
POX03851	hypothetical protein	NA	NA	NA	11.22	4.36	-1.37	0.04744	Down
POX01623	hypothetical protein	NA	NA	NA	2021.06	8000.92	1.99	4.48E-26	Up
POX07273	hypothetical protein	NA	NA	NA	61.83	246.37	1.99	5.61E-17	Up
POX06803	hypothetical protein	NA	NA	NA	27.51	109.67	2.00	6.87E-09	Up
POX07643	hypothetical protein	NA	NA	NA	76.62	29.76	-1.36	0.000341	Down
POX03345	hypothetical protein	NA	NA	NA	14.07	56.10	2.00	1.36E-06	Up
POX07388	hypothetical protein	NA	NA	NA	15.14	60.75	2.00	9.29E-07	Up
POX09728	hypothetical protein	NA	NA	NA	3.11	12.50	2.01	0.002343	Up
POX09314	hypothetical protein	NA	NA	NA	60.21	23.44	-1.36	0.000259	Down
POX07425	hypothetical protein	NA	NA	NA	87.39	34.12	-1.36	1.64E-05	Down
POX03645	hypothetical protein	NA	NA	NA	4.83	19.48	2.01	0.000497	Up
POX00360	hypothetical protein	NA	NA	NA	174.57	707.24	2.02	3.73E-19	Up
POX03526	hypothetical protein	NA	NA	NA	17.34	6.78	-1.36	0.02352	Down
POX06576	hypothetical protein	NA	NA	NA	538.35	210.40	-1.36	8.24E-13	Down
POX05864	hypothetical protein	NA	NA	NA	1.26	5.11	2.02	0.004848	Up
POX05007	hypothetical protein	NA	NA	NA	608.24	2469.69	2.02	3.5E-30	Up
POX08799	hypothetical protein	NA	NA	NA	42.68	16.69	-1.35	0.00497	Down
POX07371	hypothetical protein	NA	NA	NA	220.81	900.18	2.03	8.07E-31	Up
POX08379	hypothetical protein	NA	NA	NA	38.24	157.12	2.04	5.67E-14	Up
POX09092	hypothetical protein	NA	NA	NA	23.89	9.35	-1.35	0.017353	Down
POX03640	hypothetical protein	NA	NA	NA	41.86	16.40	-1.35	0.001078	Down

POX07954	hypothetical protein	NA	NA	NA	53.09	218.60	2.04	3.5E-15	Up
POX07562	hypothetical protein	NA	NA	NA	4.16	17.16	2.05	0.001433	Up
POX08268	hypothetical protein	NA	NA	NA	83.42	32.73	-1.35	0.000189	Down
POX00972	hypothetical protein	NA	NA	IPR004827:Basic-leucine zipper (bZIP) transcription factor	134.59	52.85	-1.35	3.4E-06	Down
POX03447	putative bifunctional polygalacturonase/pectin methylesterase	Polygalacturonase	CE8;GH28	NA	14.98	62.10	2.05	4.61E-05	Up
POX04050	hypothetical protein	NA	NA	NA	85.11	352.87	2.05	1.71E-18	Up
POX02693	hypothetical protein	NA	NA	NA	41.18	16.23	-1.34	0.00193	Down
POX00289	hypothetical protein	NA	NA	NA	19.13	79.35	2.05	3.11E-09	Up
POX06604	hypothetical protein	NA	NA	NA	98.85	39.05	-1.34	7.13E-06	Down
POX05208	hypothetical protein	NA	NA	NA	1514.23	601.07	-1.33	1.29E-10	Down
POX06606	hypothetical protein	NA	NA	NA	55.52	22.07	-1.33	0.001827	Down
POX00969	hypothetical protein	NA	NA	NA	309.70	123.19	-1.33	1.29E-09	Down
POX09597	hypothetical protein	NA	NA	NA	2.40	10.01	2.06	0.002532	Up
POX00144	hypothetical protein	NA	AA7	NA	487.86	2061.65	2.08	2.07E-27	Up
POX01223	hypothetical protein	NA	NA	NA	106.34	450.91	2.08	1.7E-23	Up
POX04929	hypothetical protein	NA	NA	NA	58.55	23.33	-1.33	0.0005	Down
POX08786	hypothetical protein	NA	NA	NA	194.84	826.22	2.08	7.64E-15	Up
POX08694	hypothetical protein	NA	AA1	NA	101.59	431.55	2.09	2E-12	Up
POX06006	hypothetical protein	NA	NA	NA	78.99	338.00	2.10	3.15E-14	Up
POX00067	hypothetical protein	NA	AA2	NA	479.51	2052.99	2.10	1.31E-20	Up
POX01379	hypothetical protein	NA	NA	NA	194.04	78.02	-1.31	2.08E-06	Down
POX02580	hypothetical protein	NA	NA	NA	2.99	12.83	2.10	0.001	Up
POX05855	hypothetical protein	NA	NA	NA	2.72	11.68	2.10	0.001433	Up
POX09734	hypothetical protein	NA	NA	NA	496.53	200.78	-1.31	9.24E-10	Down
POX00666	hypothetical protein	NA	NA	NA	175.28	70.94	-1.30	2.11E-07	Down
POX01935	hypothetical protein	NA	NA	NA	27.06	10.97	-1.30	0.008591	Down
POX00270	hypothetical protein	NA	NA	NA	10.28	44.49	2.11	3.33E-06	Up
POX07757	hypothetical protein	NA	NA	NA	203.35	82.43	-1.30	4.26E-05	Down
POX09821	hypothetical protein	NA	NA	NA	90.97	395.25	2.12	3.26E-11	Up
POX00271	hypothetical protein	NA	NA	NA	117.13	511.07	2.13	3.09E-22	Up
POX01940	hypothetical protein	NA	NA	NA	8.93	38.97	2.13	5.47E-05	Up
POX02051	hypothetical protein	NA	NA	NA	56.73	23.08	-1.30	0.000442	Down
POX06651	hypothetical protein	NA	NA	NA	48.85	19.88	-1.30	0.000994	Down
POX03603	hypothetical protein	NA	NA	NA	402.66	163.92	-1.30	2.77E-07	Down

POX01263	hypothetical protein	NA	NA	NA	69.16	302.56	2.13	5.29E-22	Up
POX03073	hypothetical protein	NA	NA	NA	7.81	34.33	2.14	6.09E-06	Up
POX09563	NA	NA	NA	NA	5.05	22.22	2.14	8.02E-05	Up
POX07891	putative alpha-L-arabinofuranosidase	beta-xylosidase/alpha-L-arabinofuranosidase	CBM35;CBM36;CBM6;GH43	NA	461.97	2034.97	2.14	2.05E-24	Up
POX08280	hypothetical protein	NA	NA	NA	56.58	23.05	-1.30	0.001068	Down
POX00634	hypothetical protein	NA	NA	NA	396.08	161.48	-1.29	1.28E-09	Down
POX04905	hypothetical protein	NA	NA	NA	38.46	15.76	-1.29	0.003764	Down
POX08811	hypothetical protein	NA	NA	NA	304.70	125.56	-1.28	4.1E-07	Down
POX07552	hypothetical protein	NA	NA	NA	30.56	12.67	-1.27	0.009254	Down
POX02714	putative alpha, alpha-trehalose-phosphate synthase	NA	GT20	NA	143.33	59.72	-1.26	6.44E-05	Down
POX00866	hypothetical protein	NA	NA	NA	119.18	49.81	-1.26	1.05E-05	Down
POX06067	putative alpha-mannosidase	NA	GH92	NA	36.76	15.42	-1.25	0.004263	Down
POX01636	hypothetical protein	NA	NA	NA	70.55	29.62	-1.25	0.00061	Down
POX05330	hypothetical protein	NA	NA	NA	369.55	155.65	-1.25	6.07E-09	Down
POX03262	hypothetical protein	NA	NA	NA	432.16	182.66	-1.24	1.32E-07	Down
POX00155	hypothetical protein	NA	NA	NA	433.23	183.46	-1.24	4.56E-09	Down
POX02757	hypothetical protein	NA	NA	NA	6.60	29.12	2.14	2.59E-05	Up
POX05868	hypothetical protein	NA	NA	NA	3.43	15.22	2.15	0.001058	Up
POX00914	hypothetical protein	NA	NA	NA	193.38	82.02	-1.24	1.78E-06	Down
POX02983	putative trehalose phosphorylase	NA	GT4	NA	50.61	21.48	-1.24	0.001178	Down
POX08371	hypothetical protein	NA	NA	NA	348.70	1556.16	2.16	1.46E-26	Up
POX00605	hypothetical protein	NA	NA	NA	255.64	109.29	-1.23	3.87E-07	Down
POX04374	hypothetical protein	NA	NA	NA	10.76	48.04	2.16	1.28E-07	Up
POX07332	hypothetical protein	NA	NA	NA	73.59	31.49	-1.22	0.000843	Down
POX03133	hypothetical protein	NA	NA	IPR001138:Fungal transcriptional regulatory protein, N-terminal//IPR007087: Zinc finger, C2H2-	28.03	12.00	-1.22	0.017983	Down
POX04829	hypothetical protein	NA	NA	NA	782.41	335.41	-1.22	3.01E-07	Down
POX01001	hypothetical protein	NA	NA	NA	996.35	4456.58	2.16	4.02E-42	Up
POX09603	hypothetical protein	NA	NA	NA	1.43	6.48	2.18	0.002029	Up
POX04316	hypothetical protein	NA	NA	NA	399.51	1820.92	2.19	4.12E-24	Up
POX00142	hypothetical protein	NA	NA	NA	135.76	620.75	2.19	6.12E-33	Up

POX06528	hypothetical protein	NA	NA	NA	233.23	100.05	-1.22	6.18E-05	Down
POX04035	hypothetical protein	NA	NA	NA	7718.56	3313.17	-1.22	1.23E-13	Down
POX09719	hypothetical protein	NA	NA	NA	20.41	8.77	-1.22	0.047522	Down
POX06225	hypothetical protein	NA	NA	NA	177.00	76.19	-1.22	1.96E-05	Down
POX07573	putative beta-N-acetylhexosaminidase	NA	GH3	NA	212.56	983.26	2.21	1.89E-05	Up
POX09598	hypothetical protein	NA	NA	NA	5.41	25.19	2.22	2.6E-05	Up
POX00412	hypothetical protein	NA	NA	NA	218.69	1023.09	2.23	1.07E-22	Up
POX00597	hypothetical protein	NA	NA	NA	40.70	190.86	2.23	9.01E-15	Up
POX03596	hypothetical protein	NA	NA	NA	21.15	9.11	-1.22	0.042694	Down
POX07623	hypothetical protein	NA	NA	NA	99.09	42.69	-1.21	7.03E-05	Down
POX09366	hypothetical protein	NA	NA	NA	476.43	205.27	-1.21	7.05E-07	Down
POX02694	hypothetical protein	NA	NA	NA	49.89	21.50	-1.21	0.002381	Down
POX01610	hypothetical protein	NA	NA	NA	679.89	3195.21	2.23	1.38E-38	Up
POX01624	hypothetical protein	NA	CE10;CE1	NA	75.28	354.70	2.24	8.78E-22	Up
POX08696	hypothetical protein	NA	NA	NA	4.18	19.73	2.24	0.000105	Up
POX00050	hypothetical protein	NA	CE10	NA	312.12	1488.46	2.25	4.61E-30	Up
POX09715	hypothetical protein	NA	NA	NA	2.21	10.56	2.25	0.000903	Up
POX01564	hypothetical protein	NA	NA	NA	142.03	681.10	2.26	5.32E-26	Up
POX03255	hypothetical protein	NA	NA	NA	993.65	4842.89	2.29	2.71E-52	Up
POX07456	hypothetical protein	NA	NA	NA	1410.91	608.21	-1.21	1.77E-10	Down
POX07964	hypothetical protein	NA	NA	NA	117.91	575.61	2.29	6.93E-06	Up
POX08354	putative UDP-galactose: ceramide 1-beta-galactosyltransferase	NA	GT1	NA	6.81	33.46	2.30	9.86E-06	Up
POX09783	hypothetical protein	NA	NA	NA	212.59	91.66	-1.21	3.02E-06	Down
POX08025	hypothetical protein	NA	NA	NA	29.07	143.52	2.30	0.000116	Up
POX08954	carbohydrate binding domain-containing protein	NA	NA	NA	1075.64	5328.72	2.31	3.25E-20	Up
POX08946	hypothetical protein	NA	NA	NA	152.47	65.92	-1.21	8.73E-05	Down
POX07377	hypothetical protein	NA	NA	NA	68.28	29.63	-1.20	0.000494	Down
POX04622	hypothetical protein	NA	NA	IPR001138:Fungal transcriptional regulatory protein, N-terminal//IPR007219: Fungal specific transcription factor	89.10	38.70	-1.20	0.000133	Down
POX05796	hypothetical protein	NA	NA	NA	153.99	67.04	-1.20	2.97E-05	Down
POX02229	hypothetical protein	NA	NA	NA	44.68	19.48	-1.20	0.003108	Down
POX09776	hypothetical protein	NA	NA	NA	1031.85	450.79	-1.19	3.46E-11	Down

POX05886	hypothetical protein	NA	NA	NA	267.86	117.17	-1.19	1.84E-07	Down
POX04212	hypothetical protein	NA	NA	NA	326.88	1627.58	2.32	4.58E-31	Up
POX02412	putative glucoamylase	NA	CBM20;GH15	NA	960.67	4839.95	2.33	5.41E-16	Up
POX04569	hypothetical protein	NA	NA	NA	15.46	78.22	2.34	2.14E-08	Up
POX08683	hypothetical protein	NA	NA	NA	3.81	19.30	2.34	4.93E-05	Up
POX08610	hypothetical protein	NA	NA	NA	18.01	7.88	-1.19	0.045858	Down
POX00351	hypothetical protein	NA	NA	NA	42.14	18.46	-1.19	0.005742	Down
POX07595	hypothetical protein	NA	NA	NA	66.64	29.20	-1.19	0.001778	Down
POX08682	hypothetical protein	NA	NA	NA	13.35	67.75	2.34	3.59E-10	Up
POX03044	hypothetical protein	NA	NA	NA	269.79	118.23	-1.19	4.15E-07	Down
POX05862	hypothetical protein	NA	NA	NA	9.83	50.48	2.36	4.04E-07	Up
POX09337	hypothetical protein	NA	NA	NA	1852.57	9522.93	2.36	2.44E-24	Up
POX03679	hypothetical protein	NA	NA	NA	1088.88	478.29	-1.19	8.54E-12	Down
POX02346	hypothetical protein	NA	NA	NA	38.02	16.71	-1.19	0.007247	Down
POX06983	xyloglucan-specific endo-beta-D-1, 4-glucanase	Endo-beta-1,4-glucanase	GH12	NA	120.52	620.85	2.36	2.01E-36	Up
POX09351	hypothetical protein	NA	NA	NA	137.67	60.56	-1.18	0.002197	Down
POX05887	hypothetical protein	NA	NA	NA	15.98	83.28	2.38	4.74E-09	Up
POX05863	hypothetical protein	NA	NA	NA	9.69	50.51	2.38	1.56E-07	Up
POX00922	hypothetical protein	NA	NA	NA	6.25	32.70	2.39	1.02E-06	Up
POX05939	putative endo-beta-1,3-glucanase	NA	GH55	NA	124.70	54.88	-1.18	0.000422	Down
POX04716	hypothetical protein	NA	NA	NA	4.55	24.23	2.41	3.74E-05	Up
POX03613	hypothetical protein	NA	NA	NA	81.53	36.04	-1.18	0.000682	Down
POX09623	hypothetical protein	NA	NA	NA	7.34	39.37	2.42	5.49E-07	Up
POX05857	hypothetical protein	NA	NA	NA	2.82	15.16	2.43	0.000189	Up
POX06098	hypothetical protein	NA	NA	NA	473.76	2569.95	2.44	6.9E-38	Up
POX08784	hypothetical protein	NA	NA	NA	1.94	10.63	2.45	0.000298	Up
POX06549	hypothetical protein	NA	NA	NA	370.70	164.09	-1.18	1.63E-06	Down
POX03793	hypothetical protein	NA	NA	NA	126.02	56.05	-1.17	9.3E-05	Down
POX05832	hypothetical protein	NA	NA	NA	28.07	155.30	2.47	3.96E-17	Up
POX03084	hypothetical protein	NA	NA	NA	79.98	35.61	-1.17	0.000364	Down
POX06427	hypothetical protein	NA	NA	NA	9.21	51.38	2.48	1.79E-08	Up
POX00919	hypothetical protein	NA	NA	NA	86.95	491.06	2.50	1.1E-21	Up
POX08654	hypothetical protein	NA	NA	NA	239.96	106.87	-1.17	8.35E-06	Down
POX02897	hypothetical protein	NA	NA	NA	1355.37	605.08	-1.16	1.33E-09	Down
POX04932	hypothetical protein	NA	NA	NA	777.75	347.27	-1.16	7.69E-06	Down
POX05492	hypothetical protein	NA	NA	NA	203.38	1149.26	2.50	1.77E-40	Up
POX05898	hypothetical protein	NA	NA	NA	141.46	63.26	-1.16	0.001799	Down

POX07412	hypothetical protein	NA	NA	NA	39.21	222.39	2.50	3.19E-23	Up
POX09183	hypothetical protein	NA	NA	NA	135.56	60.63	-1.16	2.46E-05	Down
POX00975	hypothetical protein	NA	NA	IPR001138:Fungal transcriptional regulatory protein, N-terminal//IPR007219: Fungal specific transcription factor	664.84	297.48	-1.16	2.03E-08	Down
POX02151	hypothetical protein	NA	NA	NA	451.31	203.27	-1.15	1.15E-05	Down
POX03396	hypothetical protein	NA	NA	NA	33.27	188.86	2.50	3.74E-18	Up
POX01980	hypothetical protein	NA	NA	NA	67.20	382.80	2.51	2.23E-29	Up
POX00812	hypothetical protein	NA	NA	NA	28.52	163.19	2.52	9.95E-21	Up
POX03532	hypothetical protein	NA	NA	NA	43.94	257.83	2.55	1.44E-14	Up
POX00907	hypothetical protein	NA	NA	NA	14.43	84.83	2.56	2.31E-13	Up
POX06144	hypothetical protein	NA	NA	NA	64.72	381.73	2.56	1.08E-19	Up
POX03761	hypothetical protein	NA	NA	NA	4.63	27.36	2.56	2.01E-06	Up
POX09609	hypothetical protein	NA	NA	NA	7.66	45.47	2.57	6.14E-09	Up
POX08452	hypothetical protein	NA	NA	NA	8.31	49.86	2.58	2.49E-09	Up
POX05870	hypothetical protein	NA	NA	NA	7.42	45.33	2.61	1.45E-07	Up
POX00908	hypothetical protein	NA	NA	NA	9.14	56.39	2.63	1.25E-08	Up
POX05865	hypothetical protein	NA	NA	NA	1.56	9.72	2.64	9.41E-05	Up
POX06804	hypothetical protein	NA	NA	NA	776.57	4888.59	2.65	5.2E-62	Up
POX04952	hypothetical protein	NA	NA	NA	5.67	35.77	2.66	4.93E-08	Up
POX05888	hypothetical protein	NA	NA	NA	4.07	25.77	2.66	1.74E-06	Up
POX06828	hypothetical protein	NA	NA	NA	5.10	32.43	2.67	1.87E-07	Up
POX04233	hypothetical protein	NA	NA	NA	74.51	33.69	-1.15	0.000397	Down
POX03347	hypothetical protein	NA	NA	NA	528.68	240.16	-1.14	1.51E-06	Down
POX04535	hypothetical protein	NA	NA	NA	230.97	105.16	-1.14	8.53E-07	Down
POX03928	hypothetical protein	NA	NA	NA	27.76	177.68	2.68	6.04E-12	Up
POX05856	hypothetical protein	NA	NA	NA	2.27	14.94	2.72	1.64E-05	Up
POX08024	hypothetical protein	NA	NA	NA	73.79	492.81	2.74	7.5E-07	Up
POX09125	hypothetical protein	NA	NA	NA	26.58	177.76	2.74	5.71E-20	Up
POX03886	hypothetical protein	NA	NA	IPR001356:Homeobox//IPR009057:Homeodomain-like	20.39	137.00	2.75	9.25E-18	Up
POX04831	hypothetical protein	NA	NA	NA	182.34	83.04	-1.13	6E-06	Down
POX01206	putative endo-beta-1,4-glucanase	Endo-beta-1,4-glucanase	GH5	NA	167.17	76.26	-1.13	5.27E-06	Down
POX06171	hypothetical protein	NA	NA	NA	27.64	12.64	-1.13	0.030906	Down

POX02070	hypothetical protein	NA	CE10;CE1	NA	216.56	99.42	-1.12	1.46E-06	Down
POX02762	hypothetical protein	NA	NA	NA	38.51	17.69	-1.12	0.010168	Down
POX06396	hypothetical protein	NA	NA	IPR007087:Zinc finger, C2H2-type	102.83	694.08	2.75	1.03E-40	Up
POX06300	hypothetical protein	NA	NA	NA	46.28	312.79	2.76	5.16E-25	Up
POX01165	hypothetical protein	NA	NA	NA	1.67	11.29	2.76	3.72E-05	Up
POX08765	hypothetical protein	NA	NA	NA	124.84	57.39	-1.12	0.000105	Down
POX06205	hypothetical protein	NA	NA	NA	127.11	58.55	-1.12	5.03E-05	Down
POX09698	hypothetical protein	NA	NA	IPR007219:Fungal specific transcription factor	1015.16	467.87	-1.12	1.93E-08	Down
POX04568	hypothetical protein	NA	NA	NA	27.07	12.51	-1.11	0.02521	Down
POX05233	hypothetical protein	NA	NA	NA	132.61	902.17	2.77	2.41E-37	Up
POX09234	hypothetical protein	NA	NA	NA	11.73	80.19	2.77	3.22E-11	Up
POX00522	hypothetical protein	NA	NA	NA	34.77	16.09	-1.11	0.019487	Down
POX09605	hypothetical protein	NA	NA	NA	3.65	25.80	2.82	3.85E-07	Up
POX04562	hypothetical protein	NA	NA	NA	1.99	14.15	2.83	1.11E-05	Up
POX03864	hypothetical protein	NA	NA	NA	5.17	36.90	2.84	1.96E-08	Up
POX04451	hypothetical protein	NA	CE10	NA	18.48	133.63	2.85	6.85E-19	Up
POX03292	hypothetical protein	NA	NA	NA	155.80	72.15	-1.11	0.000833	Down
POX00934	hypothetical protein	NA	NA	NA	59.89	460.62	2.94	7.04E-21	Up
POX01423	hypothetical protein	NA	NA	NA	1353.24	626.81	-1.11	3E-07	Down
POX00122	hypothetical protein	NA	NA	NA	15138.74	7012.54	-1.11	4.01E-09	Down
POX03556	hypothetical protein	NA	NA	NA	48.10	22.29	-1.11	0.005686	Down
POX07203	hypothetical protein	NA	NA	NA	80.66	37.42	-1.11	0.000787	Down
POX07880	hypothetical protein	NA	NA	NA	56.38	26.20	-1.11	0.004673	Down
POX00971	hypothetical protein	NA	NA	NA	238.40	110.80	-1.11	6E-06	Down
POX06248	hypothetical protein	NA	NA	NA	63.86	29.71	-1.10	0.001559	Down
POX00702	hypothetical protein	NA	NA	NA	118.97	55.36	-1.10	0.000139	Down
POX09469	hypothetical protein	NA	NA	Winged helix repressor DNA-binding:IPR011991:Winged helix repressor DNA-binding	2.36	18.26	2.95	1.53E-06	Up
POX05959	hypothetical protein	NA	NA	NA	224.60	104.59	-1.10	1.62E-05	Down

POX01951	hypothetical protein	NA	NA	NA	2.69	20.88	2.96	6.65E-07	Up
POX09611	hypothetical protein	NA	NA	NA	6.21	48.82	2.97	1.93E-09	Up
POX08757	hypothetical protein	NA	NA	NA	3.32	26.79	3.01	4.5E-08	Up
POX05365	hypothetical protein	NA	NA	NA	66.03	542.95	3.04	7.91E-31	Up
POX02472	hypothetical protein	NA	NA	NA	82.05	38.26	-1.10	0.001061	Down
POX03800	hypothetical protein	NA	NA	NA	308.56	2541.23	3.04	7.58E-37	Up
POX01508	hypothetical protein	NA	NA	NA	508.15	236.97	-1.10	1.53E-05	Down
POX08768	putative alpha, alpha-trehalase	NA	GH65	NA	445.90	208.24	-1.10	0.001706	Down
POX07806	hypothetical protein	NA	NA	NA	305.35	142.86	-1.10	2.44E-07	Down
POX05818	hypothetical protein	NA	NA	NA	18.78	156.56	3.06	1.35E-17	Up
POX01080	hypothetical protein	NA	NA	NA	301.15	2569.64	3.09	2.85E-74	Up
POX09471	hypothetical protein	NA	NA	NA	10.71	92.35	3.11	2.63E-14	Up
POX03575	hypothetical protein	NA	NA	NA	221.57	103.68	-1.10	1.11E-05	Down
POX08698	hypothetical protein	NA	NA	NA	869.30	406.98	-1.09	4.64E-06	Down
POX02682	hypothetical protein	NA	NA	IPR001005:Myb, DNA-binding//IPR009057:H	438.94	205.58	-1.09	3.63E-07	Down
POX07419	hypothetical protein	NA	NA	NA	57.89	514.46	3.15	5.32E-42	Up
POX08018	hypothetical protein	NA	NA	NA	417.19	196.05	-1.09	4.39E-07	Down
POX09233	hypothetical protein	NA	AA3;AA8	NA	72.38	34.03	-1.09	0.002463	Down
POX03135	hypothetical protein	NA	NA	NA	874.42	412.76	-1.08	4.44E-08	Down
POX03101	hypothetical protein	NA	NA	NA	693.67	327.52	-1.08	2.92E-09	Down
POX07201	putative exo-beta-1, 3-glucanase	NA	GH17	NA	9489.82	4487.65	-1.08	1.31E-10	Down
POX05016	hypothetical protein	NA	NA	NA	3.57	31.99	3.16	7.1E-09	Up
POX06715	hypothetical protein	NA	NA	NA	87.58	41.58	-1.07	0.000449	Down
POX08759	hypothetical protein	NA	NA	NA	3.74	33.74	3.17	1.86E-09	Up
POX09397	hypothetical protein	NA	NA	NA	1.64	15.05	3.20	4.9E-07	Up
POX08691	hypothetical protein	NA	NA	NA	378.40	3512.13	3.21	4.59E-32	Up
POX06740	hypothetical protein	NA	NA	NA	96.16	45.67	-1.07	0.001905	Down
POX09473	hypothetical protein	NA	NA	NA	11.16	104.68	3.23	1.27E-07	Up
POX01842	hypothetical protein	NA	NA	NA	4.48	42.09	3.23	6.92E-11	Up
POX03854	hypothetical protein	NA	NA	NA	31.53	14.97	-1.07	0.031837	Down
POX03826	hypothetical protein	NA	NA	NA	200.92	95.51	-1.07	4.62E-05	Down
POX02354	hypothetical protein	NA	NA	NA	85.22	40.59	-1.07	0.000754	Down
POX07560	hypothetical protein	NA	NA	NA	74.87	35.69	-1.07	0.002699	Down
POX05644	hypothetical protein	NA	NA	NA	284.67	135.75	-1.07	6.64E-06	Down
POX09390	hypothetical protein	NA	NA	NA	129.25	61.67	-1.07	0.000196	Down

POX03616	hypothetical protein	NA	NA	NA	179.28	85.63	-1.07	2.78E-05	Down
POX07889	hypothetical protein	NA	NA	NA	479.36	229.25	-1.06	1.18E-07	Down
POX04807	hypothetical protein	NA	NA	NA	4.67	44.12	3.24	1.67E-09	Up
POX05789	hypothetical protein	NA	NA	NA	29.06	276.09	3.25	3.99E-38	Up
POX05575	hypothetical protein	NA	NA	NA	22.68	217.27	3.26	1.94E-19	Up
POX08760	putative alpha, alpha-trehalose-phosphate synthase	NA	GT20;GT4	NA	64.83	624.82	3.27	9.7E-56	Up
POX08579	hypothetical protein	NA	NA	NA	9.83	96.00	3.29	6.81E-18	Up
POX07624	hypothetical protein	NA	NA	NA	267.39	128.00	-1.06	2.84E-06	Down
POX01868	hypothetical protein	NA	NA	NA	84.66	40.53	-1.06	0.002752	Down
POX02392	hypothetical protein	NA	NA	NA	3448.49	1652.48	-1.06	4.65E-09	Down
POX00120	hypothetical protein	NA	NA	NA	1782.31	856.35	-1.06	9.81E-07	Down
POX06778	hypothetical protein	NA	NA	NA	86.13	41.39	-1.06	0.001096	Down
POX02211	hypothetical protein	NA	NA	NA	114.07	54.89	-1.06	0.000829	Down
POX02299	hypothetical protein	NA	NA	NA	315.50	151.97	-1.05	6.71E-06	Down
POX02809	hypothetical protein	NA	NA	NA	184.89	89.06	-1.05	2.23E-05	Down
POX04155	hypothetical protein	NA	NA	NA	355.37	171.46	-1.05	5.73E-05	Down
POX04371	hypothetical protein	NA	NA	IPR006600:Centromere protein B, DNA-binding region//IPR009057:H	34.90	16.85	-1.05	0.023633	Down
POX06037	hypothetical protein	NA	NA	NA	81.43	39.31	-1.05	0.001726	Down
POX02718	hypothetical protein	NA	NA	NA	489.95	236.83	-1.05	9.18E-06	Down
POX02783	hypothetical protein	NA	NA	NA	88.03	42.56	-1.05	0.001555	Down
POX00082	hypothetical protein	NA	NA	NA	99.36	972.18	3.29	3.08E-47	Up
POX08189	hypothetical protein	NA	NA	NA	608.57	294.51	-1.05	5.8E-08	Down
POX01929	hypothetical protein	NA	NA	NA	21.25	208.97	3.30	6.75E-23	Up
POX05223	hypothetical protein	NA	NA	NA	6.14	60.49	3.30	2E-14	Up
POX04360	hypothetical protein	NA	NA	NA	971.42	9581.96	3.30	8.31E-54	Up
POX01356	glucoamylase Amy15A	NA	CBM20;GH15	NA	471.10	4880.73	3.37	1.65E-11	Up
POX07971	putative chitinase	NA	GH18	NA	1895.67	19822.42	3.39	7.57E-52	Up
POX07797	hypothetical protein	NA	NA	NA	6.31	67.56	3.42	3.28E-15	Up
POX06213	hypothetical protein	NA	NA	NA	10.29	112.90	3.46	7.05E-15	Up
POX07380	putative lysozyme	NA	GH25	NA	1174.52	13112.80	3.48	3.41E-47	Up
POX05259	hypothetical protein	NA	NA	NA	51.69	586.15	3.50	1.54E-56	Up
POX09470	hypothetical protein	NA	NA	NA	5.04	58.82	3.54	5.7E-14	Up
POX02502	hypothetical protein	NA	NA	NA	51.38	24.87	-1.05	0.013706	Down
POX00373	hypothetical protein	NA	NA	NA	70.12	34.08	-1.04	0.002101	Down

POX05866	hypothetical protein	NA	NA	NA	3.26	39.76	3.61	1.61E-10	Up
POX05569	hypothetical protein	NA	NA	NA	15.50	189.46	3.61	1.84E-19	Up
POX04369	hypothetical protein	NA	NA	NA	45.15	553.67	3.62	4.34E-09	Up
POX05854	hypothetical protein	NA	NA	NA	2.35	29.24	3.63	9.09E-10	Up
POX07772	hypothetical protein	NA	NA	NA	52.80	666.83	3.66	8.73E-60	Up
POX08032	hypothetical protein	NA	NA	NA	33.33	437.18	3.71	5E-50	Up
POX00786	hypothetical protein	NA	NA	NA	15.97	222.93	3.80	6.69E-38	Up
POX08687	hypothetical protein	NA	NA	NA	33.75	541.29	4.00	3.07E-48	Up
POX07390	hypothetical protein	NA	NA	NA	1.99	36.61	4.20	4.71E-13	Up
POX08690	hypothetical protein	NA	NA	NA	729.05	13528.23	4.21	1.2E-66	Up
POX09284	hypothetical protein	NA	NA	NA	595.40	12160.28	4.35	2.9E-150	Up
POX08305	hypothetical protein	NA	NA	NA	221.37	4726.83	4.42	2.8E-117	Up
POX06628	hypothetical protein	NA	NA	NA	328.76	160.22	-1.04	2.19E-06	Down
POX05501	hypothetical protein	NA	AA3;AA8	NA	12.40	266.16	4.42	3.08E-47	Up
POX00925	hypothetical protein	NA	NA	NA	107.11	2434.78	4.51	5.37E-78	Up
POX08693	hypothetical protein	NA	NA	NA	173.04	3938.34	4.51	5.93E-82	Up
POX03572	hypothetical protein	NA	NA	NA	44.89	21.90	-1.04	0.017404	Down
POX07618	hypothetical protein	NA	NA	NA	41.58	20.29	-1.03	0.014058	Down
POX02471	hypothetical protein	NA	NA	NA	30.35	14.81	-1.03	0.039545	Down
POX08252	hypothetical protein	NA	NA	NA	96.86	47.30	-1.03	0.001716	Down
POX07341	hypothetical protein	NA	NA	NA	52.42	1229.49	4.55	7.04E-93	Up
POX01906	hypothetical protein	NA	NA	NA	8.33	216.48	4.70	3.58E-42	Up
POX00452	hypothetical protein	NA	NA	NA	12.50	370.51	4.89	1.77E-49	Up
POX01234	hypothetical protein	NA	NA	NA	42.76	1481.95	5.12	3.4E-108	Up
POX05092	hypothetical protein	NA	NA	NA	8.64	439.41	5.67	3.91E-66	Up
POX08686	hypothetical protein	NA	NA	NA	117.69	7306.54	5.96	5.7E-167	Up
POX03096	hypothetical protein	NA	NA	NA	1201.85	588.87	-1.03	3.2E-06	Down
POX08692	hypothetical protein	NA	NA	NA	90.76	5782.14	5.99	1.8E-132	Up
POX08783	hypothetical protein	NA	NA	NA	28.94	2177.37	6.23	2.8E-135	Up
POX04673	sphingolipid long chain base-responsive protein	NA	NA	NA	710.02	348.53	-1.03	0.000311	Down
POX00118	hypothetical protein	NA	NA	NA	156.54	76.84	-1.03	4.15E-05	Down
POX08689	hypothetical protein	NA	NA	NA	96.12	7884.20	6.36	2.2E-112	Up
POX00427	hypothetical protein	NA	NA	NA	63.76	31.38	-1.02	0.005357	Down
POX07613	hypothetical protein	NA	NA	NA	346.06	170.41	-1.02	7.88E-07	Down
POX04938	putative glycogen branching enzyme	NA	CBM48;GH13	NA	2264.89	1116.05	-1.02	9.87E-10	Down
POX02396	hypothetical protein	NA	NA	NA	319.34	157.99	-1.02	4.88E-06	Down
POX03546	hypothetical protein	NA	NA	NA	654.37	324.49	-1.01	3.27E-06	Down

POX05961	hypothetical protein	NA	NA	NA	78.61	39.05	-1.01	0.005444	Down
POX01344	hypothetical protein	NA	NA	NA	674.30	335.10	-1.01	6.29E-07	Down
POX08688	hypothetical protein	NA	NA	NA	38.42	5462.82	7.15	8E-175	Up
